# Occupational mechanical exposures as risk factor for chronic low-back pain: a systematic review and meta-analysis

**DOI:** 10.5271/sjweh.4114

**Published:** 2023-10-01

**Authors:** Alexander Jahn, Johan Hviid Andersen, David Høyrup Christiansen, Andreas Seidler, Annett Dalbøge

**Affiliations:** 1 Danish Ramazzini Centre, Department of Occupational Medicine, Aarhus University Hospital, Aarhus, Denmark.; 2 Danish Ramazzini Centre, Department of Occupational Medicine - University Research Clinic, Goedstrup Hospital, Herning, Denmark.; 3 Elective Surgery Centre, Silkeborg Regional Hospital, Silkeborg, Denmark.; 4 Research, Regional Hospital Central Jutland, Viborg, Denmark.; 5 Department of Clinical Medicine, Aarhus University, Aarhus, Denmark.; 6 Institute and Policlinic of Occupational and Social Medicine (IPAS), Faculty of Medicine, Technische Universität Dresden, Dresden, Germany.

**Keywords:** chronicity, ergonomics, musculoskeletal disorder, occupational health, physical workload, spine

## Abstract

**Objectives:**

The association between occupational mechanical exposures and low-back pain (LBP) has been studied in several systematic reviews. However, no systematic review addressing chronic LBP exists. The aim of this systematic review and meta-analysis was to examine the association between occupational mechanical exposures and chronic LBP.

**Methods:**

The study was registered in PROSPERO. We used an existing systematic review to identify articles published before January 2014. For studies published between January 2014 and September 2022, a systematic literature search was conducted in six databases. Two authors independently excluded articles, extracted data, and assessed risk of bias and level of evidence (GRADE). Meta-analyses were conducted using random-effects models comparing highest versus lowest exposure group with sensitivity analyses based on study quality (low/moderate versus high risk of bias), study design (cohort versus case–control), and outcome definition (non-specific LBP versus specific chronic LBP).

**Results:**

Twenty-six articles were included. Highest pooled odd ratios (OR) were found for combined mechanical exposures [OR 2.2, 95% confidence interval (CI) 1.4–3.6], lifting/carrying loads (OR 1.7, 95% CI 1.4–2.2), and non-neutral postures (OR 1.5, 95% CI 1.2–1.9). For the remaining mechanical exposures (ie, whole-body vibrations, standing/walking, and sitting), OR ranged between 1.0 and 1.4. In the sensitivity analyses, generally, higher pooled OR were found in low/moderate risk of bias studies, case–control studies, and studies of specific chronic LBP.

**Conclusions:**

Moderate evidence of an association was found for lifting/carrying loads, non-neutral postures, and combined mechanical exposures. Low or very low evidence was found for whole-body vibrations, standing/walking, and sitting. Studies using standardized exposure definition, metric, and technical measurements are highly warranted.

Low-back pain (LBP) is a frequent health problem in the general population and is the leading cause of years lived with disability (1–3). From a societal perspective, LBP increases the risk of sick leave and early retirement from the labor market, decreasing income-producing assets, and increasing healthcare service expenses (4, 5). Consequently, LBP is a global health problem (3, 5, 6).

In 2020, Wu and colleagues estimated the global prevalence of LBP to be 7.5%, indicating that approximately 577 million people were affected (7). Although LBP can be temporary with a fluctuating pattern of recovery, it is estimated that 4–20% of the adult population develops a chronic condition that gradually increases with age (8–11).

LBP is defined as pain or discomfort located in the lumbar region and/or gluteal region anatomically outlined from the 12^th^ thoracic vertebra to the gluteal sulcus with or without radiating pain. The disorder is considered a complex condition, as the structural causes of pain are difficult to identify and characterize (4). Accordingly, the majority of LBP is categorized as non-specific LBP, while a specific pathoanatomical diagnosis only can be confirmed in a minority of cases (eg, radiculopathy or severe pathology affecting the lumbar spine) (4, 5).

Occupational mechanical exposures, eg, heavy lifting, repetitive movements, and non-neutral postures have been identified as risk factors for LBP in several systematic reviews (12–16) and even an overview (17). In 2014, the Swedish Council on Health Technology Assessment (SBU) published a report conducted as a systematic review and meta-analysis of the association between occupational mechanical and psychosocial exposures and back problems defined as “back trouble, sciatica, degenerative disc change, and back disease” (excluding the cervical part of the spine) (12). The SBU report identified almost 8000 potentially relevant articles, performed nearly 1000 full paper readings, and included a total of 109 moderate or high quality-rated cohort or case–control studies. Moderate evidence of an association was found for manual handling including lifting loads [odds ratio (OR) 1.32, 95% confidence interval (CI) 1.22–1.42], non-neutral work positions including spine flexion (OR 1.61, 95% CI 1.42–1.83), and whole-body vibrations (OR 1.20, 95% CI 1.04–1.38). However, in 2020, an overview of systematic reviews found conflicting evidence for spine curvatures, prolonged or occupational standing, non-neutral postures, bending and twisting movements, components of heavy physical work, and whole-body vibrations, while no association was found for prolonged or occupational sitting (17).

To our knowledge, no systematic review has specifically explored the association between occupational mechanical exposures and chronic LBP defined as pain persisting for >3 months. Therefore, the objective of this systematic review and meta-analysis was to study the association between occupational mechanical exposures and chronic LBP.

## Methods

This systematic review and meta-analysis was conducted in accordance with guidelines stated by the PRISMA-P 2015 checklist (Preferred Reporting Items for Systematic Reviews and Meta-Analyses). The study was registered in PROSPERO (the International Prospective Register of Systematic Reviews) with registration number CRD42021281996. A study protocol was prepared before the review (18). No ethical approval was needed since the systematic review and meta-analysis is based on published data.

### Search strategy

Due to the expected comprehensive literature, our literature search was separated into two parts. In the first part, articles were retrieved from the SBU report, which included articles published from 1980 to 10 January 2014 (12). In the second part, articles published after 10 January 2014, were retrieved using a systematic literature search. The search string was conducted in collaboration with a librarian and tested, so it was identical to the SBU literature search. The literature search was performed between 2–21 September 2021, and updated until 28 September 2022, using the following international electronic databases: PubMed, EMBASE, PsycInfo, CINAHL, Cochrane Library, and Web of Science (supplementary material, www.sjweh.fi/article/4114, supplementary appendix A). We used the reference management tool EndNote 20 (19) to remove article duplicates, before the remaining articles were transferred to the review management software Covidence (20). In addition, the first 100 articles on the association between occupational mechanical exposures and chronic LBP from Google Scholar was screened.

### Study selection

The selection of relevant articles was based on predefined criteria using PECOS [Population, Exposure, Comparison, Outcome, Study design (supplementary appendix B)]. Study criteria included a population in or above working age with no limitations regarding sex, demographics, and ethnicity. The outcome was defined as LBP persisting >3 months to be consistent with the “Classification of chronic pain” (21), including both specific (ie, sciatica, lumbar disc herniation, or lumbosacral damage) and non-specific LBP. We excluded articles with outcome based on pain caused by other diseases or proxy measures, ie, cancer, fractures, inflammation, or sick absenteeism. The exposure was defined as occupational mechanical exposures (eg, lifting/carrying loads, non-neutral postures, whole-body vibrations, or combined mechanical exposures) excluding exposures based on job titles or accidents/injuries only. The study design was restricted to case–control and cohort studies. Cross-sectional studies were excluded due to the lack of temporality between exposure and disease.

For articles published before 10 January 2014, 192 articles were eligible for full-text reading in the SBU report. Two authors performed full-text reading, and discrepancies were solved by a third author. For articles published after 10 January 2014, two authors independently identified eligible articles using title/abstract screening followed by full-text reading, and discrepancies were solved through discussion among the authors until consensus. Finally, reference lists in all included articles were screened for other potential articles.

### Data extraction and risk of bias assessment

Information on author, study design, population, outcome, outcome assessment, exposure, exposure assessment, and confounders was extracted from each included article. Furthermore, information on measure of association [relative risks (RR), OR, hazard ratios (HR), and prevalence ratios (PR)) with corresponding 95% CI] was extracted according to the occupational mechanical exposures divided into seven groups (ie, lifting/carrying loads, non-neutral postures, whole-body vibrations, standing/walking, sitting, combined mechanical exposures, and “other mechanical exposures”). One author extracted data and a second author checked for quality.

To critically appraise the methodological quality of each included study, we used a risk of bias tool (supplementary appendix C) developed for chronic diseases and used in several systematic reviews (16, 22–25). Risk of bias was divided into five major domains (study design, exposure, outcome, enrolment, and analysis method) and three minor domains (funding, chronology, and conflict of interests). For a study to be considered as having “low” risk of bias, all major domains and ≥1 minor domain should be rated as low risk of bias. To be considered as “moderate” risk, 4 out of 5 major domains and ≥1 minor domain should be rated as “low” risk. All other combinations were considered as high risk of bias. Two authors independently performed the risk of bias assessment. Discrepancies were solved through discussion with all authors until consensus was reached.

### Statistical analysis

The meta-analysis was conducted only to visualize whether an association between occupational mechanical exposures and chronic LBP across studies could be indicated. We excluded studies that were based on identical source populations to avoid double-counting data. If studies were based on the same population, we chose the study with the highest quality rating, and if both studies had the same quality rating, the size of the study population would determine the exclusion. In the meta-analysis, measures of association using sex-combined estimates were included when available, but if only sex-specific estimates were provided, associations for each sex were selected. We only included the measure of association between the highest exposure category compared to the lowest exposure category. A measure of association with risk estimates other than OR was considered approximately equivalent to OR if the incidence proportion of the disease was <10% (26). Articles providing estimates other than OR were tested for either <10% of an outcome or the OR was calculated if the number of participants was provided.

Pooled estimates (OR with a 95% CI) were calculated in the meta-analysis using random-effects models based on the assumption that studies cannot be assumed to provide estimates of one common, true effect (27). To estimate the proportion of the observed variance that reflects real differences among studies, I-squared was calculated, describing the percentage of variability due to heterogeneity, and was quantified using the restricted maximum likelihood method (REML) (28). We used Cochrane’s thresholds for interpretation of the I-squared statistic (29). Forest plots of studies included in the meta-analysis were constructed to visualize the overall association between occupational mechanical exposures and chronic LBP. Publication bias was evaluated using funnel plots, and we tested the asymmetry of the funnel plots by Egger’s test. If a study provided more than one measure of association (eg, men and women), the group containing the highest number of participants was included to avoid double counting. Finally, exposure–response relations were examined by extracting results from statistical tests, eg, trend tests, provided in studies. If an exposure–response relation was not statistically examined, we graphically visualized potential exposure–response relations, including risk estimates and 95% CI for each level when studies provided >3 exposure groups regarding lifting/carrying, non-neutral postures, and combined exposures.

Sensitivity analyses were conducted by stratifying studies according to our risk of bias assessment (moderate/low versus high risk of bias), study design (cohort versus case–control studies), and outcome definition (non-specific versus specific chronic LBP) to assess the influence on the pooled effect size. All statistical analyses were performed using STATA 17.0 (Stata Corp, College Station, TX, USA).

### Level of evidence of an association

Level of evidence of an association was assessed using the Grading of Recommendations Assessment, Development, and Evaluation (GRADE). By complying with guidelines proposed by The Navigation Guide, level of evidence from observational studies was started at “moderate” evidence (30). Two authors independently rated level of evidence and a third author was consulted when discrepancies occurred between ratings. The overall level of evidence was rated as “high”, “moderate”, “low”, and “very low” (supplementary appendix D) (31).

## Results

**Figure 1:**
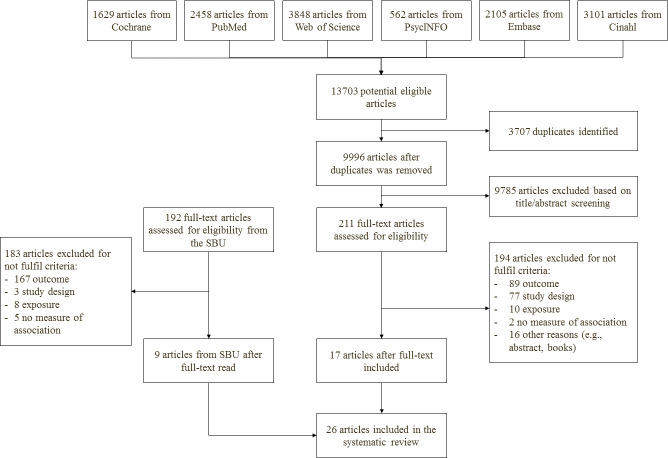
Flowchart of the literature search and exclusion of articles.

Figure 1 shows the literature search and exclusion of studies. From the SBU, 192 articles were identified for full-text reading, and nine articles were deemed eligible for inclusion. The literature searches for articles published after 10 January 2014, yielded 13 703 articles, including 3707 duplicates. After screening of 9996 articles, 9785 articles were excluded providing 211 articles eligible for full-text reading. After full-text reading, a total of 194 articles were further excluded and 17 were deemed eligible for inclusion. Supplementary appendix E presents the excluded studies and the explanation for the exclusion.

A total of 26 studies were included in the systematic review comprising 18 cohort (32–49) and eight case–control (50–57) studies. Supplementary appendix F presents a summary of study characteristics of the 26 included articles. The outcome was assessed using self-reports in 14 articles (ie, questionnaires and interviews), imaging modalities (ie, CT, MRI, and X-ray) in seven articles, and register information in five articles. Therefore, a total of 14 articles included non-specific LBP compared to 12 on specific LBP. Exposure was assessed using questionnaires in 17 articles, interview in 7 articles, observation and questionnaire in one article, and a job-exposure matrix in one article. A total of 92% of the studies were therefore based on self-reports. Studies were conducted in Denmark (39, 47), Sweden (35, 49), Norway (37), Finland (34), Germany (41, 52, 54–56), Netherlands (38, 45), France (33, 36, 48), United States (40), Mexico (53), Brazil (57), Iran (32, 46), Syria (51), Bangladesh (50), and Japan (42–44). The articles were published between 2002 and 2019.

Based on the risk of bias assessment, five studies were rated as having low risk of bias, 11 with moderate risk of bias, and 10 with high risk of bias (supplementary appendix C). The most frequent major domain receiving a high risk of bias assessment was “study design & follow-up” for cohort studies and “study design & selection” for case–control studies. The second most frequent major domain receiving a high risk of bias assessment was “exposure”. Supplementary appendix G presents measure of associations between occupational mechanical exposures and chronic LBP reported in the 26 included studies. Seven studies provided a measure of association other than OR. Of these, three had an incidence proportion of the outcome of <10% (34, 39, 49). One study reported a crude RR of 1.30 (95% CI 1.00–1.71) with an equivalent OR calculated to 1.38 (95% CI 0.98–1.94) (37), and four studies did not provide sufficient numbers of participants to be used for transformation into OR (35, 38, 41). The latter were included in the meta-analysis treated as OR, as we expected an incidence proportion similar to the included studies. Meta-analysis was not conducted for exposure groups defined as “other mechanical exposures” due to large exposure heterogeneity.

**Figure 2:**
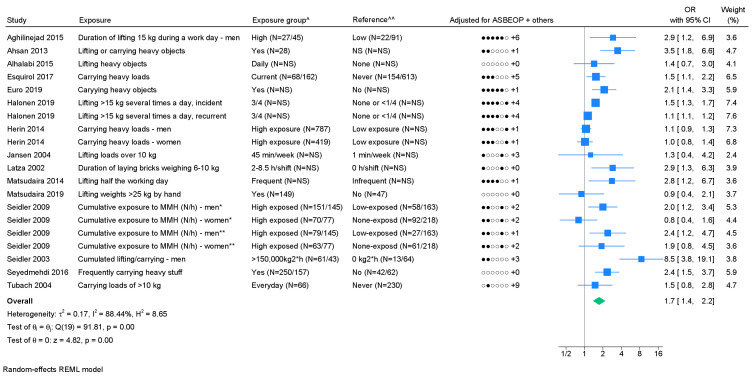
Forest plot of the association between lifting/carrying loads and chronic lower back pain (LBP). [ASBEOP=Age, Sex, BMI, Education, Other mechanical exposures, and Physical activity in leisure time. + Others = adjustments of other confounding variables besides ASBEOP; NS=not stated; kg=kilograms; MMH=manual materials handling; N/h=newton hour; H=hour.] *Cases with lumbar disc herniation. **Cases with lumbar disc narrowing. ^ In brackets, numbers of exposed persons with chronic LBP and numbers of exposed references is provided.

*Lifting/carrying loads.* A total of 17 studies included lifting/carrying loads, however four studies were based on two identical study populations. This led to the exclusion of two studies from the meta-analysis (43, 52). We found a pooled OR of 1.7 (95% CI 1.4–2.2), showing a considerable degree of heterogeneity with I^2^=88.4% (figure 2). The funnel plot (supplementary appendix H) indicated publication bias, and Egger’s test showed a significant P-value (0.02). Three studies tested and found positive exposure–response relations (38, 41, 54). Five studies presented a measure of association for ≥3 exposure groups, of which three studies indicated an exposure–response relation (supplementary appendix I). Using GRADE, moderate evidence of an association was found (table 1, supplementary appendix D).

**Table 1:** Results on the association between occupational mechanical exposures and chronic low-back pain (LBP). [OR=odds ratio; CI=confidence interval; I^2^=heterogeneity; CoE=certainty of evidence +++=moderate, ++=low, +=very low certainty.]

Exposure (N of studies)	Pooled OR		I^2^		Publication bias		Sensitivity analysis based on risk of bias						Sensitivity analysis based on study design						Sensitivity analysis based on outcome definition					CoE ^a^
					Y/N (Egger's test score)		Low / moderate			High			Cohort			Case–control			Non-specific chronic LBP			Specific chronic LBP		
	OR (95% CI)		%		%		OR (95% CI)	N		OR (95% CI)	N		OR (95% CI)	N		OR (95% CI)	N		OR (95% CI)	N		OR (95% CI)	N	
Lifting / carrying loads (15)	1.7 (1.4–2.2)		88.4		Y (0.02)		1.9 (1.4–2.5)	9		1.4 (1.0–1.9)	6		1.5 (1.2–1.8)	11		2.2 (1.3–3.8)	4		1.5 (1.2–1.8)	10		2.2 (1.4–3.4)	5	+++
Non-neutral postures (12)	1.5 (1.2–1.9)		87.2		Y (0.06) ^b^		1.7 (1.2–2.3)	7		1.3 (1.1–1.4)	5		1.3 (1.1–1.5)	8		2.1 (1.5–3.0)	4		1.3 (1.2–1.5)	8		1.7 (1.0–2.8)	4	+++
Whole-body vibrations (7)	1.4 (1.1–1.7)		46.7		Y(0.03) ^b^		1.4 (1.2–1.7)	5		1.3 (0.8–1.9)	2		1.3 (1.0–1.7)	4		1.7 (1.0–2.8)	3		1.3 (0.9–2.0)	2		1.4 (1.2–1.7)	5	++
Standing / walking (6)	1.0 (0.8–1.3)		43.4		N (0.49) ^b^		1.0 (0.8–1.2)	3		0.9 (0.4–2.2)	3		1.0 (0.9–1.2)	4		0.8 (0.2–3.6)	2		1.1 (0.9–1.5)	4		0.7 (0.3–1.7)	2	+
Sitting (6)	1.2 (1.0–1.5)		2.7		N (0.50) ^b^		1.1 (0.8–1.4)	4		1.5 (0.9–2.5)	2		1.1 (0.9–1.4)	4		1.6 (0.8–3.0)	2		1.2 (0.8-1.8)	4		1.1 (0.8–1.6)	2	++
Combined exposures (5)	2.2 (1.4–3.6)		89.9		(0.41) ^c^		2.2 (1.4–3.6)	5			0		1.2 (1.0–1.4)	3		4.2 (1.4–12.9)	2		1.2 (1.0–1.4)	1		2.8 (1.1–7.1)	4	+++

^a^ See Appendix p12-13 for clarification. ^b^ Based on few studies. ^c^ Not possible to assess due to too few studies.

**Figure 3:**
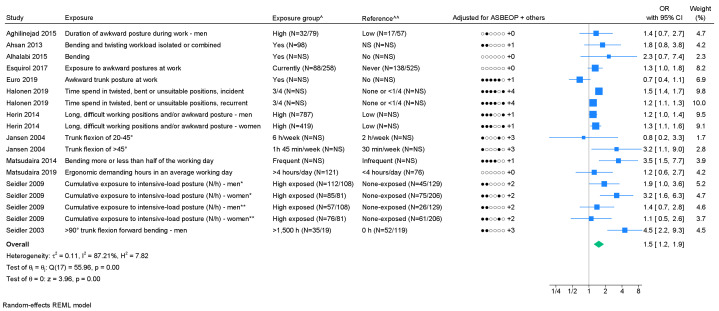
Forest plot of the association between non-neutral postures and chronic LBP. [ASBEOP=Age, Sex, BMI, Education, Other mechanical exposures, and Physical activity in leisure time. + Others = adjustments of other confounding variables besides ASBEOP; NS=not stated; kg=kilograms.] *Cases with lumbar disc herniation. **Cases with lumbar disc narrowing. ^ In brackets, numbers of exposed persons with chronic LBP and numbers of exposed references is provided. ^^ In brackets, numbers of unexposed persons with chronic LBP and numbers of unexposed references is provided. If only on number is provided, it was not possible to distinguish between, e.g., exposed persons with chronic LBP and exposed references. ^^ In brackets, numbers of unexposed persons with chronic LBP and numbers of unexposed references is provided. If only on number is provided, it was not possible to distinguish between, e.g., exposed persons with chronic LBP and exposed references.

*Non-neutral postures*. Fourteen studies on non-neutral postures were identified, however four studies were based on two identical study populations; two studies were excluded (43, 52). We found a pooled OR of 1.5 (95% CI 1.2–1.9) with I^2^=87.2% (figure 3). The funnel plot (supplementary appendix H) indicated publication bias and Egger’s test showed a close to significant P-value (0.06). Two studies tested and found positive exposure–response relations (38, 54). Two studies presented a measure of association for ≥3 exposure groups, of which both studies indicated an exposure–response relation (supplementary appendix I). Using GRADE, moderate evidence of an association was found (table 1, supplementary appendix D).

**Figure 4:**
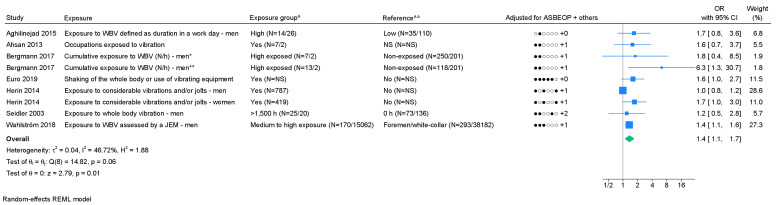
Forest plot of the association between whole-body vibrations and chronic LBP. [ASBEOP=Age, Sex, BMI, Education, Other mechanical exposures, and Physical activity in leisure time. + Others = adjustments of other confounding variables besides ASBEOP; NS=not stated; N/h=newton hours; JEM=job-exposure matrix; WBV=whole-body vibration.] *Cases with lumbar disc herniation. **Cases with lumbar disc narrowing. ^ In brackets, numbers of exposed persons with chronic LBP and numbers of exposed references is provided. ^^ In brackets, numbers of unexposed persons with chronic LBP and numbers of unexposed references is provided. If only on number is provided, it was not possible to distinguish between, e.g., exposed persons with chronic LBP and exposed references.

*Whole-body vibrations:* Seven studies on whole-body vibration were included in the meta-analysis. We found a pooled OR of 1.4 (95% CI 1.1–1.7) with I^2^=46.7% (figure 4). Based on few studies, an indication of publication bias was found (supplementary appendix H) and Egger's test showed significant P-value (0.03). Using GRADE, low level of evidence of an association was found (table 1, supplementary appendix D).

**Figure 5:**
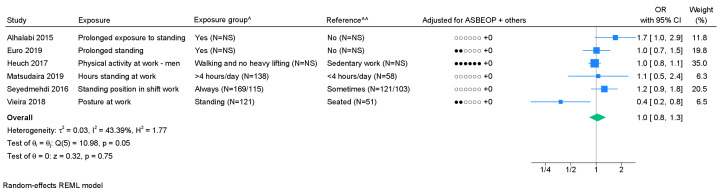
Forest plot of the association between standing or walking and chronic LBP. [ASBEOP=Age, Sex, BMI, Education, Other mechanical exposures, and Physical activity in leisure time. + Others = adjustments of other confounding variables besides ASBEOP; NS=not stated.] *Cases with lumbar disc herniation. **Cases with lumbar disc narrowing. ^ In brackets, numbers of exposed persons with chronic LBP and numbers of exposed references is provided. ^^ In brackets, numbers of unexposed persons with chronic LBP and numbers of unexposed references is provided. If only on number is provided, it was not possible to distinguish between, e.g., exposed persons with chronic LBP and exposed references.

*Standing/walking.* Six studies on standing/walking were included in the meta-analysis. We found a pooled OR of 1.0 (95% CI 0.8–1.3) with I^2^=43.4% (figure 5). Based on few studies, no indication of publication bias was found (supplementary appendix H) and Egger's test showed no significant P-value (0.49). Using GRADE, very low level of evidence of an association was found (table 1, supplementary appendix D).

**Figure 6:**
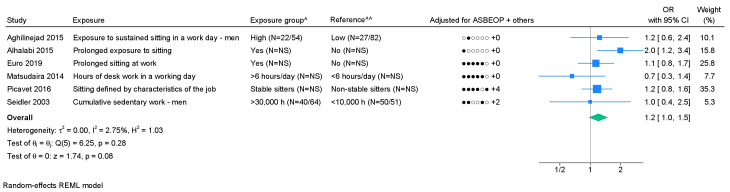
Forest plot of the association between sitting and chronic LBP. [ASBEOP=Age, Sex, BMI, Education, Other mechanical exposures, and Physical activity in leisure time. + Others = adjustments of other confounding variables besides ASBEOP; NS=not stated; H=hours.] *Cases with lumbar disc herniation. **Cases with lumbar disc narrowing. ^ In brackets, numbers of exposed persons with chronic LBP and numbers of exposed references is provided. ^^ In brackets, numbers of unexposed persons with chronic LBP and numbers of unexposed references is provided. If only on number is provided, it was not possible to distinguish between, e.g., exposed persons with chronic LBP and exposed references.

**Figure 7:**
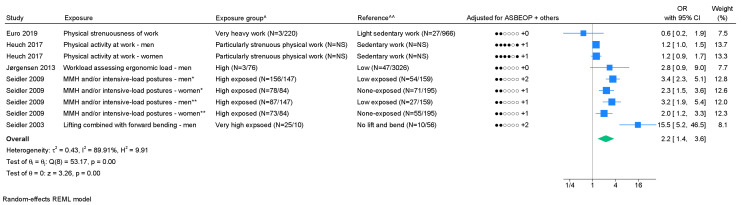
Association between combined mechanical exposures and chronic LBP. [ASBEOP=Age, Sex, BMI, Education, Other mechanical exposures, and Physical activity in leisure time. + Others = adjustments of other confounding variables besides ASBEOP; NS=not stated; MMH=manual materials handling.] *Cases with lumbar disc herniation. **Cases with lumbar disc narrowing. ^ In brackets, numbers of exposed persons with chronic LBP and numbers of exposed references is provided. ^^ In brackets, numbers of unexposed persons with chronic LBP and numbers of unexposed references is provided. If only on number is provided, it was not possible to distinguish between, e.g., exposed persons with chronic LBP and exposed references.

*Sitting.* Among eight studies on sitting, two were based on an identical study population, and one study failed to provide 95% CI; two studies were excluded from the meta-analysis (43, 50). We found a pooled OR of 1.2 (95% CI 1.0–1.5) with I^2^=2.7% (figure 6). Based on few studies, no indication of publication bias was found (supplementary appendix H) and Egger's test showed no significant P-value (0.50). Low level of evidence of an association was found (table 1, supplementary appendix D).

*Combined mechanical exposures*. Of the nine studies on combined mechanical exposures, four were based on identical study populations, and one study failed to provide a 95% CI; four studies were excluded from the meta-analysis (47, 50, 52, 56). We found a pooled OR of 2.2 (95% CI 1.4–3.6) with I^2^=89.9% (figure 7). Based on few studies, publication bias could not be evaluated. One study tested and found a positive exposure–response relation (55). Three studies presented a measure of association for ≥3 exposure groups, of which two studies indicated exposure–response relation (supplementary appendix I). Moderate level of evidence of an association was found (table 1, supplementary appendix D).

### Sensitivity analysis

In the sensitivity analyses, we generally found higher OR for all mechanical exposures in low/moderate risk of bias studies, case–control studies, and studies of specific chronic LBP except for standing/walking showing a pooled OR of 1.0 (95% CI 0.8–1.3) (table 1). We were not able to perform sensitivity analysis based on the exposure assessment, as 93% of all included studies were based on self-reports.

## Discussion

Based on the 26 included articles, we found moderate evidence of an association in regards to lifting/carrying loads, non-neutral postures, and combined mechanical exposures with pooled OR ranging between 1.5 and 2.2. Conversely, low or very low evidence was found for whole-body vibrations, standing/walking, and sitting with pooled OR of 1.0–1.4.

The strengths of this study were the thorough systematic literature search performed in several databases in collaboration with a librarian, and the exclusion of studies, risk of bias assessment, data extraction, and the evaluation of level of evidence independently conducted by two authors. We included all potentially relevant articles with few restrictions to the methodological qualities to provide a thorough overview of the existing scientific literature. Conversely, the literature search was limited to English and the Nordic languages, potentially excluding relevant articles. Funnel plots, Egger's tests, and sensitivity analyses were performed with few studies, and the results should be interpreted with caution.

To our knowledge, this is the first systematic review and meta-analysis on the association between occupational mechanical exposures and chronic LBP. When comparing our results with former systematic reviews on LBP, we found some degree of consistency (12–16). However, according to the overview conducted by Swain et al (17), only weak or conflicting evidence of an association was found for occupational mechanical exposures (17). Low quality-rated systematic reviews generally ruled in favor of associations, while systematic reviews only including cohort studies identified inconsistent as well as null results (17). We also found lower risks in cohort studies compared to case–control studies. In cohort studies, baseline exposure assessments reported independently of the outcome at follow-up increase the risk of non-differential misclassification, which often provide attenuation bias (58). Conversely, in case–control studies, participants were aware of the outcome status when assessing exposure, which might increase the risk of overestimation among cases as a consequence of differential misclassification.

The case–control studies typically included specific compared to non-specific chronic LBP. In the sensitivity analysis, higher risks were found for specific compared to non-specific chronic LBP except for standing/walking and sitting with pooled OR of 1.0 and 1.2. We chose to combine non-specific and specific chronic LBP, knowing that it can encompass chronic pain independent of physiological conditions causing chronic LBP. Moreover, trajectory patterns of pain tend to contain high variability meaning that an episode of LBP could be a temporary condition in an ongoing chronic condition, which can cause uncertainty in the categorization of chronic LBP cases depending on the time of measurement.

We excluded studies with no precise definition of the location of back pain, eg, if a study used the term “back problems” instead of “low back pain”. Furthermore, chronicity was defined as >3 months with LBP. If a period was not provided in a study, or if a study combined groups of cases with, eg, 30–90 days and >90 days with LBP, they were excluded. The result of our definition of chronic LBP might have reduced the number of included studies, but increased the comparability between studies concerning the outcome.

We found indication of higher risks in low-to-moderate risk of bias studies compared to high risk of bias rated studies independent of study design. One of the most frequent major domains receiving a high risk of bias assessment was “exposure”. Exposure assessments in the included studies were all based on questionnaires or interview, except for two studies which were based on a JEM and observations. It is generally considered that self-reports are less reliable compared to objective measurements (59, 60). To account for potential bias derived from self-reported exposure assessments, only exposure measurements using validated questionnaires or interviews were rated without major risk of bias. We did not include a series of studies (the Dutch SMASH studies) where mechanical exposures were assessed by video recordings and technical measurements, as LBP was defined as regular or prolonged pain in the previous 12 months (61, 62). Coenen and colleagues (61) found an adjusted OR of 2.03 (95% CI 1.23–3.36) for lifting >25 kg >15 times/working day and an adjusted OR of 1.45 (95% CI 0.77–2.73) spending >5% of work time in >60 degree trunk flexion. Hamberg-van Reenen (62)and colleagues measured employee’s physical capacity and exposure to occupational physical factors to define balance and imbalance groups. They found an adjusted relative risk of 1.35 (95% CI 1.08–1.68) for the imbalanced group measuring static endurance and trunk flexion of >30 degrees. When comparing the results of the Dutch SMASH studies with our systematic review, no major differences were found despite distinct variations in exposure assessments.

The measure of associations for the specific occupational mechanical exposure were fairly consistent between studies, pointing in favor of a positive association. However, large heterogeneity exists in exposure definitions, metrics, and scales, supported by generally high I^2^ values. The exposure heterogeneity made it difficult to compare results between studies, even when the same exposure domain was evaluated (eg, lifting). The differences further excluded identification of exposure thresholds and time windows in our systematic review.

In the meta-analyses and forest plots, we used the highest exposure group versus. the lowest exposure group. Since the highest exposure groups often contain fewer participants, it affects the standard error of a given estimate resulting in broader confidence intervals with an increased risk of type 2 error.

The inclusion of confounding variables varied considerably between studies with some of the studies adjusting for no or a few confounders (32, 46, 51, 57). Several of the studies did not control for essential potential confounders such as educational level, leisure time activity, or other occupational mechanical or psychosocial exposures, possibly leading to bias in the results. Controlling for leisure time activity might especially be important for standing, walking, and sitting compared to, eg, whole body vibration, as these three occupational mechanical exposures also occur during leisure time. Conversely, the literature has shown that workers who have a high physical activity at work tend to be fairly inactive during leisure time (63). However, we expect that educational level, leisure time activity, and occupational exposures to some extents are correlated, and therefore adjustment might underestimate the measure of associations. Despite the variations in number of included potential confounders, we chose to extract adjusted measure of associations when available to increase the internal validity of each estimate and our conclusion.

### Perspectives

The level of evidence of an association found in our systematic review has practical implications. In the clinical context, it must be taken into account when communicating with patients about the nature of their illness and forming recommendations on sick leave or job change. In a political/administrative context, it is important for decisions on preventive strategies, compensation of illnesses as occupational disorders, and the prioritization of further research.

In future studies, we highly recommend assessment of LBP using validated tools to distinguish between minor pain episodes and chronic LBP. Furthermore, we suggest distinguishing between pain that is limited to the lower back and LBP, which occurs in association with pain at other anatomical locations. Studies using technical measurements are indeed warranted to enhance to obtain valid exposure information. We also suggest that exposure–response relations should be studied across increasing levels of exposure rather than few exposure groups, or even dichotomizing. In continuation, exposure intensity, frequency, and duration should be given for the whole work time. Finally, we furthermore suggest seeing LBP as a much broader concept. Results from the CUPID study (64, 65) have shown that illnesses such as non-specific musculoskeletal pain appear to be a lot more complex and of multifactorial nature, eg, depending on culturally determined influences. This means to include contextual factors in the understanding of LBP (66).

### Concluding remarks

In this systematic review, we found moderate evidence of an association for exposure to lifting/carrying loads, non-neutral postures, and combined mechanical exposures. Some indications of exposure–response relations were found, but the current scientific literature did not allow identification of safe exposure thresholds. Conversely, low or very low evidence was found for whole-body vibrations, standing/walking, and sitting.

## Supplementary material

Supplementary material
