# Supplementary material for: Occupational mechanical exposures as risk factor for chronic low-back pain: a systematic review and meta-analysis
Source: Scand J Work Environ Health. 2023 Sep 29;49(7):453–65. doi: 10.5271/sjweh.4114 (PMC10838064; doi:10.5271/sjweh.4114)

Occupational mechanical exposures as risk factor for chronic low back pain: a systematic review and meta-analysis

Alexander Jahn, Johan H. Andersen, David H. Christiansen, Andreas Seidler, and Annett Dalbøge.

Supplementary Appendices

Appendix A. Literature search 31
Table S2. Literature search 31
Appendix B. PECOS 36
Table S3. Inclusion and exclusion criteria for the systematic review. 36
Appendix C. Risk of bias assessment tool. 37
Table S4. Risk of bias assessment tool, case-control studies. 37
Table S5. Risk of bias assessment tool, cohort studies. 38
Table S6. Risk of bias assessment of all 26 included articles. 39
Appendix D. GRADE. 40
Table S7. Quality level. 40
Table S8. Downgrading and upgrading criteria. 40
Table S9. GRADE assessment based on the 26 included studies. 41
Appendix E. Excluded articles. 42
Table S10. Excluded articles based on full-text read and reason for exclusion. 42
Appendix F. Summary of characteristics. 61
Table S12. Summary of characteristics of the 26 included articles. 61
Appendix G. Measure of association. 68
Table S13. Measure of association between occupational mechanical exposures and chronic LBP.68
Appendix H. Funnel plots 84
Figure 8. Funnel plots for each occupational mechanical exposure. 84
Appendix I. Exposure-response relations. 85
Lifting/carrying loads 85
Non-neutral postures 86
Combined mechanical exposures 87

Appendix A. Literature search

Table S2. Literature search
Our literature search consisted of a block search containing four blocks combining them with the Boolean operator "AND" between blocks and "OR" within blocks.

Block 1. Work.

	**Free text search in title and abstract**
**Mesh**	
"Occupations"[Mesh]	occupation*, employ*, job*, working condition*, work-related work-load*, work-place*, work environment*
"Occupational Health"[Mesh]	
"Occupational Diseases"[Mesh]	
"Occupational Exposure"[Mesh]	
"Occupational Groups"[Mesh]	
"Work"[Mesh]	
"Workplace"[Mesh]	
"Workload"[Mesh]	
"Women, Working"[Mesh]	
"Employment"[Mesh]	

Block 2. Exposure.

	**Free text search in title and abstract**
**Mesh**	
**Mechanical stress**	
"Stress, Mechanical"[Mesh]	
**Organisational and psychosocial factors**	
"Stress, Psychological"[Mesh]	
**Development of force**	lift*, carry*, hold*, pull*, drag*, push*, manual handling, force*, biomechanic*, physical demand*, physically demand*.
"Lifting"[Mesh]	
"Weight-Bearing"[Mesh]	
"Biomechanics"[Mesh]	
"Moving and Lifting Patients"[Mesh]	
"Physical Exertion"[Mesh]	
**Working postures**	flexion*, extension*, turning*, sitting*, kneeling*, twisting*, bending, sedentary, walking*, reaching, squatting, standing, postural balance, static AND posture, awkward AND posture.
"Torsion, Mechanical"[Mesh]	
"Postural Balance"[Mesh]	
"Walking"[Mesh]	
**Working movement**	repetitive movement*, monotonous work, dynamic AND posture, relaxation, recovery of function, static work, dynamic load.
"Recovery of Function"[Mesh]	

”Relaxation”[Mesh]	
**Influence and demand**	decision latitude, work demand*, job demand*, high demand*, low control, work control, job control, work influence*, demand resource*, lack of control, job strain, work strain.
**Effort and reward**	effort reward*, time pressure*, work overload*, recuperation*, recovery.
**Social support and relations in the workplace**	support system*, social network*, emotional support, justice*, injustice*, interaction*, interpersonal relation*.
”Social Support”[Mesh]	
”Employee Performance "Appraisal”[Mesh]	
”Organizational Culture”[Mesh]	
"Justice/psychology”[Mesh]	
”Communication/psychology”[Mesh]	
”Interpersonal Relations”[Mesh]	
**Job satisfaction**	Boredom, job satisfaction, work satisfaction, coping, work ability.
”Job Satisfaction”[Mesh]	
”Employee Grievances”[Mesh]	
**Education and learning:**	skill discretion*, staff development.
”Staff Development”[Mesh]	
**Conflict, violence or harassment**	harass*, workplace conflict*, workplace violen*, silent workplace*, victimization*, bullying, role ambiguity, role conflict*, work role*, discrimination.
”Bullying”[Mesh]	
”Prejudice”[Mesh]	
”Social Discrimination”[Mesh]	
**Working time**	working hour*, working time, shift work*, work shift*, day-time, night-time, temporary work, full-time, part-time, flexible work*, lean production.
”Work Schedule Tolerance”[Mesh]	
**Job insecurity**	organizational change, job security, job insecurity.
”Personnel Downsizing”[Mesh]	
**Chemical and biological substances**	pollut*, indoor air*, airborne, passive smok*, solvent*, smok* AND pollute*.
”Air Pollution”[Mesh]	
”Air Pollutants”[Mesh]	
”Solvents”[Mesh]	
”Fluids and Secretions”[Mesh]	
**Contact with chemicals**	hazardous chemical*, hazardous material*, hazardous substance*, toxic action*, pesticide*, poison*.
”Toxic Actions”[Mesh]	
**Noise**	noise*

”Noise”[Mesh]	
**Vibrations**	vibrat*, driving, vehicle*, truck*, lorry/lorries, automobile*, car/cars, buses, hand tool*, hand-held tool*, power tool*.
”Vibration”[Mesh]	
”Automobile Driving”[Mesh]	
”Motor Vehicles”[Mesh]	
**Radiation**	radiation*
”Radiation”[Mesh]	
”Air Pollution, Radioactive”[Mesh]	
**Temperature**	climate*, cold temperature*, hot temperature*.
”Hot Temperature”[Mesh]	
”Cold Temperature”[Mesh]	
”Climate”[Mesh]	
**Infected materials**	contagious* communicable disease*
”Communicable Diseases”[Mesh]	

Block 3. Back problems

	**Free text search in title and abstract**
**Mesh**	
**Back problems**	
"Back" [Mesh]	back, spine*, spinal*, trunk*, lumbar*, pelvis*, sacrum, lumbo-sacral*, lumbosacral*, intervertebral disk*, intervertebral disc*, thoracic vertebrae, thoracic vertebra.
"Spine” [Mesh]	
"Pelvis" [Mesh]	
"Pain" [Mesh]	pain, ache*, musculoskeletal disease*, musculoskeletal disorder*, cumulative trauma disorder*, nerve entrapment.
"Pain Measurement" [Mesh]	
"Cumulative Trauma Disorders" [Mesh]	
"Musculoskeletal Diseases" [Mesh]	
"Back Pain" [Mesh]	back pain, backache*, back injur*, spinal disease*, spine disease*, spinal injur* OR spine injur*, intervertebral disk degeneration, intervertebral disc degeneration, spinal osteochondros*, spine osteochondros*, Scheuermann*, spinal stenos*, spondylitis, spondylarthritis, spondylosis, lumbago, sciatica, pelvic pain.
"Back Injuries" [Mesh]	
"Spinal Diseases" [Mesh]	
"Pelvic Pain" [Mesh]	
"Sciatica" [Mesh]	

Block 4 Language restrictions and publication date.

**Language restrictions**	
English, Danish, Norwegian, or Swedish.	

Publication date	
From 2014/01/01 to 2021/09/21.<br><br>The updated search was done including articles from 2021 until September 28, 2022, and adding the following to, e.g., Medline: (2021/9/2:2022/10/1[pdat])	

Aggregated search string from Medline:
((("Back"[MeSH Terms] OR "spine"[MeSH Terms] OR "Pelvis"[MeSH Terms] OR ("Back"[Title/Abstract] OR "spine*"[Title/Abstract] OR "spinal*"[Title/Abstract] OR "trunk*"[Title/Abstract] OR "lumbar*"[Title/Abstract] OR "pelvis*"[Title/Abstract] OR "sacrum"[Title/Abstract] OR "lumbo sacral*"[Title/Abstract] OR "lumbosacral*"[Title/Abstract] OR "intervertebral disk*"[Title/Abstract] OR "intervertebral disc*"[Title/Abstract] OR "thoracic vertebrae"[Title/Abstract] OR "thoracic vertebra"[Title/Abstract])) AND ("Pain"[MeSH Terms] OR "Pain Measurement"[MeSH Terms] OR "Cumulative Trauma Disorders"[MeSH Terms] OR "Musculoskeletal Diseases"[MeSH Terms] OR ("Pain"[Title/Abstract] OR "ache*"[Title/Abstract] OR "musculoskeletal disease*"[Title/Abstract] OR "musculoskeletal disorder*"[Title/Abstract] OR "cumulative trauma disorder*"[Title/Abstract] OR "nerve entrapment"[Title/Abstract]))) OR ("Back Pain"[MeSH Terms] OR "Back Injuries"[MeSH Terms] OR "Spinal Diseases"[MeSH Terms] OR "Pelvic Pain"[MeSH Terms] OR "Sciatica"[MeSH Terms] OR ("Back Pain"[Title/Abstract] OR "backache*"[Title/Abstract] OR "back injur*"[Title/Abstract] OR "spinal disease*"[Title/Abstract] OR "spine disease*"[Title/Abstract] OR "spinal injur*"[Title/Abstract] OR "spine injur*"[Title/Abstract] OR "intervertebral disk degeneration"[Title/Abstract] OR "intervertebral disc degeneration"[Title/Abstract] OR "spinal osteochondros*"[Title/Abstract] OR "spine osteochondros*"[Title/Abstract] OR "scheuermann*"[Title/Abstract] OR "spinal stenos*"[Title/Abstract] OR "spondylitis"[Title/Abstract] OR "spondylarthritis"[Title/Abstract] OR "spondylosis"[Title/Abstract] OR "lumbago"[Title/Abstract] OR "Sciatica"[Title/Abstract] OR "Pelvic Pain"[Title/Abstract]))) AND ("Work"[MeSH Terms] OR "Workload"[MeSH Terms] OR "Workplace"[MeSH Terms] OR "Occupations"[MeSH Terms] OR "Occupational Health"[MeSH Terms] OR "Occupational Diseases"[MeSH Terms] OR "Occupational Groups"[MeSH Terms] OR "Occupational Exposure"[MeSH Terms] OR "women, working"[MeSH Terms] OR "Employment"[MeSH Terms:noexp] OR (("work-related"[Title/Abstract] OR "work load*"[Title/Abstract] OR "workload*"[Title/Abstract] OR "workplace*"[Title/Abstract] OR "workplace*"[Title/Abstract] OR "work environment*"[Title/Abstract] OR "working condition*"[Title/Abstract] OR "occupation*"[Title/Abstract] OR "job"[Title/Abstract] OR "employ*"[Title/Abstract]) NOT ("medline"[Filter] OR "oldmedline"[Filter]))) AND ("stress, mechanical"[MeSH Terms] OR "Lifting"[MeSH Terms] OR "Moving and Lifting Patients"[MeSH Terms] OR "Weight-Bearing"[MeSH Terms] OR "Physical Exertion"[MeSH Terms] OR "torsion, mechanical"[MeSH Terms] OR "Postural Balance"[MeSH Terms] OR "Walking"[MeSH Terms] OR "recovery of function"[MeSH Terms] OR "Relaxation"[MeSH Terms] OR ("static"[Title/Abstract] AND ("postural"[All Fields] OR "posturally"[All Fields] OR "posture"[MeSH Terms] OR "posture"[All Fields] OR "postures"[All Fields] OR "postured"[All Fields] OR "posturing"[All Fields])) OR ("awkward"[Title/Abstract] AND ("postural"[All Fields] OR "posturally"[All Fields] OR "posture"[MeSH Terms] OR "posture"[All Fields] OR "postures"[All Fields] OR "postured"[All Fields] OR "posturing"[All Fields])) OR ("dynamic"[Title/Abstract] AND ("postural"[All Fields] OR "posturally"[All Fields] OR "posture"[MeSH Terms] OR "posture"[All Fields] OR "postures"[All Fields] OR "postured"[All Fields] OR "posturing"[All Fields])) OR "static work"[Title/Abstract] OR "dynamic load*"[Title/Abstract] OR "lift*"[Title/Abstract] OR "carry*"[Title/Abstract] OR "hold*"[Title/Abstract] OR "pull*"[Title/Abstract] OR "drag*"[Title/Abstract] OR "push*"[Title/Abstract] OR "manual handling"[Title/Abstract] OR "force*"[Title/Abstract] OR "biomechanic*"[Title/Abstract] OR "walking*"[Title/Abstract] OR "Postural Balance"[Title/Abstract] OR "flexion*"[Title/Abstract] OR "extension*"[Title/Abstract] OR "turning"[Title/Abstract] OR "sitting"[Title/Abstract] OR "kneeling"[Title/Abstract] OR "squatting"[Title/Abstract] OR "twisting"[Title/Abstract] OR "bending"[Title/Abstract] OR "reaching"[Title/Abstract] OR "standing"[Title/Abstract] OR "sedentary"[Title/Abstract] OR "repetitive movement*"[Title/Abstract] OR "monotonous work"[Title/Abstract] OR "Relaxation"[Title/Abstract] OR "recovery of function"[Title/Abstract] OR "physical demand*"[Title/Abstract] OR "physically demand*"[Title/Abstract] OR ("stress, psychological"[MeSH Terms] OR "Social Support"[MeSH Terms] OR "Job Satisfaction"[MeSH Terms] OR "Work Schedule Tolerance"[MeSH Terms] OR "Employee Performance Appraisal"[MeSH Terms] OR "Employee Grievances"[MeSH Terms] OR "social justice/psychology"[MeSH Terms] OR "Personnel Downsizing"[MeSH Terms] OR "Staff Development"[MeSH Terms] OR "Organizational Culture"[MeSH Terms] OR "Bullying"[MeSH Terms] OR "Prejudice"[MeSH Terms] OR "Social Discrimination"[MeSH Terms] OR "Interpersonal Relations"[MeSH Terms] OR "communication/psychology"[MeSH Terms] OR "psychosocial"[Title/Abstract] OR "job strain"[Title/Abstract] OR "work strain"[Title/Abstract] OR "work demand*"[Title/Abstract] OR "job demand*"[Title/Abstract] OR "high demand*"[Title/Abstract] OR "low control"[Title/Abstract] OR "lack of control"[Title/Abstract] OR "work control"[Title/Abstract] OR "job control"[Title/Abstract] OR "decision latitude"[Title/Abstract] OR "work influence*"[Title/Abstract] OR "demand resource*"[Title/Abstract] OR "effort reward*"[Title/Abstract] OR "time pressure*"[Title/Abstract] OR "recuperation*"[Title/Abstract] OR "work overload*"[Title/Abstract] OR "work over load*"[Title/Abstract] OR "recovery"[Title/Abstract] OR "coping"[Title/Abstract] OR "work ability"[Title/Abstract] OR "Social Support"[Title/Abstract] OR "support system*"[Title/Abstract] OR "social network*"[Title/Abstract] OR "emotional support"[Title/Abstract] OR "interpersonal relation*"[Title/Abstract] OR "interaction*"[Title/Abstract] OR "justice*"[Title/Abstract] OR "injustice*"[Title/Abstract] OR "Job Satisfaction"[Title/Abstract] OR "work satisfaction"[Title/Abstract] OR "boredom"[Title/Abstract] OR "skill discretion*"[Title/Abstract] OR "Staff Development"[Title/Abstract] OR "discrimination"[Title/Abstract] OR "harass*"[Title/Abstract] OR (("workplace"[MeSH Terms] OR "workplace"[All Fields] OR ("work"[All Fields] AND "place"[All Fields]) OR "work-place"[All Fields]) AND "conflict*"[Title/Abstract]) OR "workplace violen*"[Title/Abstract] OR "work place violen*"[Title/Abstract] OR "Bullying"[Title/Abstract] OR "victimization*"[Title/Abstract] OR (("silent"[All Fields] OR "silently"[All Fields] OR "silents"[All Fields]) AND "workplace*"[Title/Abstract]) OR "role ambiguity"[Title/Abstract] OR "roleconflict*"[Title/Abstract] OR "work role*"[Title/Abstract] OR "working hour*"[Title/Abstract] OR "working time"[Title/Abstract] OR "daytime"[Title/Abstract] OR "night-time"[Title/Abstract] OR "shift work*"[Title/Abstract] OR "work shift*"[Title/Abstract] OR "temporary work*"[Title/Abstract] OR "full-time"[Title/Abstract] OR "part-time"[Title/Abstract] OR "flexible work*"[Title/Abstract] OR "organizational change"[Title/Abstract] OR "organisational change"[Title/Abstract] OR "lean production"[Title/Abstract] OR "job security"[Title/Abstract] OR "job insecurity"[Title/Abstract]) OR ("Air Pollutants"[MeSH Terms] OR "Air Pollution"[MeSH Terms] OR "Fluids and Secretions"[MeSH Terms] OR "Toxic Actions"[MeSH Terms] OR "Solvents"[MeSH Terms] OR "pollut*"[Title/Abstract] OR "indoor air*"[Title/Abstract] OR "airborne"[Title/Abstract] OR ("smok*"[Title/Abstract] AND "pollut*"[Title/Abstract]) OR "passive smok*"[Title/Abstract] OR "hazardous chemical*"[Title/Abstract] OR "hazardous material*"[Title/Abstract] OR "hazardous substance*"[Title/Abstract] OR "toxic action*"[Title/Abstract] OR "pesticide*"[Title/Abstract] OR "poison*"[Title/Abstract] OR "solvent*"[Title/Abstract]) OR ("Radiation"[MeSH Terms] OR "air pollution, radioactive"[MeSH Terms] OR "Hot Temperature"[MeSH Terms] OR "Cold Temperature"[MeSH Terms] OR "Climate"[MeSH Terms] OR "radiation*"[Title/Abstract] OR "climate*"[Title/Abstract] OR "cold

temperature*"[Title/Abstract] OR "hot temperature*"[Title/Abstract]) OR ("Communicable Diseases"[MeSH Terms] OR "communicable disease*"[Title/Abstract] OR "contagious*"[Title/Abstract]) OR ("Noise"[MeSH Terms] OR "noise*"[Title/Abstract]) OR ("Vibration"[MeSH Terms] OR "Motor Vehicles"[MeSH Terms] OR "Automobile Driving"[MeSH Terms] OR "driving"[Title/Abstract] OR "automobile*"[Title/Abstract] OR "car"[Title/Abstract] OR "cars"[Title/Abstract] OR "vibrat*"[Title/Abstract] OR "vehicle*"[Title/Abstract] OR "truck*"[Title/Abstract] OR "lorry"[Title/Abstract] OR "lorries"[Title/Abstract] OR "buses"[Title/Abstract] OR "hand held tool*"[Title/Abstract] OR "hand tool*"[Title/Abstract] OR "power tool*"[Title/Abstract])) AND ("english"[Language] OR "danish"[Language] OR "norwegian"[Language] OR "swedish"[Language])

Appendix B. PECOS

Table S3. Inclusion and exclusion criteria for the systematic review.

	Inclusion criteria (PECOS)	Exclusion criteria
**Population**	Adults in or above working age.	Adults never in work or students.
**Exposure**	Occupational mechanical exposures, e.g., working postures, lifting, carrying, whole-body vibration, driving, walking, or kneeling etc.<br><br>Quantified exposure measure through self-report, interview, observation, technical measure or job-exposure-matrix.	Chemical or biological substances, radiation, heat or cold, accidents/injuries, job titles, or non-related occupational exposures.
**Comparison**	A measure of association between occupational exposures and chronic LBP expressed in an appropriate risk estimate, e.g., odds ratio, relative risk, hazard ratio, or prevalence ratio with corresponding 95% confidence interval.	If no appropriate risk estimate is provided or not possible to calculate based on information eligible in the study.
**Outcome**	Non-specific chronic low back pain lasting $\geq$3 months.<br><br>Specific chronic low back pain including lumbago, sciatica, lumbar herniated disc and lumbosacral degenerative changes with or without radiculopathy.<br><br>Outcome measured with self-report, interview, clinical diagnosis (e.g., ICD code), imaging modalities (e.g., computed tomography, x-ray, or magnetic resonance imaging), or compensation/insurance claim.	Injuries based on accidents, inherent pain, pain caused by other diseases or conditions such as cancer, fractures, or inflammation.<br><br>Proxy measures to chronic LBP, e.g., sickness absenteeism.<br><br>Studies not reporting on chronic LBP according to our definition as pain lasting $\geq$3 months.
**Study design**	Randomised control trials; non-randomised control trials; prospective cohort; retrospective cohort; case-control; case-cohort; nested case-control.<br><br>Original study in full text and peer-reviewed.<br><br>In English, Danish, Swedish or Norwegian.<br><br>Published from January the 10th 2014.	Cross-sectional studies, systematic reviews, in vitro studies, studies on health economics, studies not addressing any risk factors related to work, studies with less than 30 participants, and Studies in other languages than in the inclusion criteria.<br><br>Animal trials.<br><br>Conference notes, books, letters to editor, editorial pages, protocols, reports and abstracts.

LBP=low back pain; ICD=International Classification of Diseases.

Appendix C. Risk of bias assessment tool.

Table S4. Risk of bias assessment tool, case-control studies.

Case-Control Study	Yes	No	Unclear
**Major domain 1 – study design and selection**			
**Were the cases recruited in an accepTable Sway?** Consider the following:<br>● Are the cases representative of a population, clearly defined and differentiated from controls?<br>● Was there an established reliable system for selecting all the cases?<br>● Were inclusion and exclusion criteria explicit and applied similarly to all eligible cases?			
**Were the controls selected in an accepTable Sway?** Consider the following:<br>● Are the controls representative of a population and clearly defined?<br>● Are the same inclusion and exclusion criteria for cases used to select controls (equally applied) and matched appropriately?<br>● Is it clearly established that controls are non-cases?			
**Is the participation rate satisfactory?** Consider the following:<br>● Are there large differences between the two groups?<br>● Is the participation rate low?			
**Major domain 2 – Exposure**			
**Was the exposure accurately measured to minimise bias?** Consider the following:<br>● Is the exposure clearly defined?<br>● Do measurements truly reflect what it is supposed to measure (have they been validated?).<br>● Is the method of assessment reliable?			
**Major domain 3 – Outcome**			
**Was the outcome accurately measured to minimise bias?** Consider the following:<br>● Is the outcome clearly defined?<br>● Do measurements truly reflect what it is supposed to measure (have they been validated?).<br>● Is the method of assessment reliable?			
**Major domain 4 – Non-participants**			
**Is comparison made between participants and non-participants?**<br>Consider the following:<br>● Is similarities or differences established?			
**Major domain 5 – Analysis method**			
**Was the analysis method adequate?** Consider the following:<br>● Are the main potential confounders identified and taken into account in the analysis?<br>● Were adequate statistical models used to reduce bias?			
**Minor domain 1 – Funding**			
**Was the source of funding provided?** Consider the following:<br>● Was the study affected by sponsors?<br>● Did sponsoring organisation participate in the analysis?			
**Minor domain 2 – Chronology**			
**Could chronology be established?** Consider the following:<br>● Was the timeframe sufficient to see an association between the exposure and outcome?			
**Minor domain 3 – Conflict of interest**			
**Was the study without any conflict of interest?** Consider the following:<br>● Was the study affected by the authors affiliations or interests?			

Table S5. Risk of bias assessment tool, cohort studies.

Cohort Study	Yes	No	Unclear
**Major domain 1 – study design and selection**			
**Was the cohort recruited in an accepTable Sway?** Consider the following:<br>● Is it representative of a defined population and clearly specified?<br>● Are groups comparable in all respects other than the factor under investigation?<br>● Was everybody included who should have been?			
**Was the follow-up of subjects accepTable S?** Consider the following:<br>● Conventionally, a 20% drop out rate is accepTable S, but observational studies conducted over longer periods, a higher drop-out rate is to be expected.<br>● Were losses to follow-up taken into account in the analysis (sensitivity analysis, described etc.)?			
**Major domain 2 – Exposure**			
**Was the exposure accurately measured to minimise bias?** Consider the following:<br>● Is the exposure clearly defined?<br>● Do measurements truly reflect what it is supposed to measure (have they been validated?).<br>● Is the method of assessment reliable?<br>● Were all the subjects classified into exposure groups using the same procedure?			
**Major domain 3 – Outcome**			
**Was the outcome accurately measured to minimise bias?** Consider the following:<br>● Is the outcome clearly defined?<br>● Do measurements truly reflect what it is supposed to measure (have they been validated?).<br>● Is the method of assessment reliable?<br>● Were the measurement methods similar in the different groups?<br>● If blinding is not possible, is there some recognition that knowledge of exposure status could influence the assessment of the outcome?			
**Major domain 4 – Enrolment**			
**Was the outcome taken into account at enrolment?** Consider the following:<br>● Some participants might have the outcome at the time of enrolment. Is it assessed at baseline in the analysis?			
**Major domain 5 – Analysis method**			
**Was the analysis method adequate?** Consider the following:<br>● Are the main potential confounders identified and taken into account in the analysis?<br>● Were adequate statistical models used to reduce bias?			
**Minor domain 1 – Funding**			
**Was the source of funding provided?** Consider the following:<br>● Was the study affected by sponsors?<br>● Did sponsoring organization participate in the analysis?			
**Minor domain 2 – Chronology**			
**Could chronology be established?** Consider the following:<br>● Was the timeframe sufficient to see an association between the exposure and outcome?<br>● Was the follow-up long enough for the outcome to occur?			
**Minor domain 3 – Conflict of interest**			
**Was the study without any conflict of interest?** Consider the following:<br>● Was the study affected by the authors affiliations or interests?			

Table S6. Risk of bias assessment of all 26 included articles.

		Domains							
		**Major**					**Minor**		
**References**	**Quality score**	**1**	**2**	**3**	**4**	**5**	**6**	**7**	**8**
**Aghilinejad 2015**	Low risk	(+)	(+)	(+)	(+)	(+)	(-)	(+)	(?)
**Ahsan 2013**	High risk	(-)	(?)	(+)	(-)	(-)	(-)	(?)	(?)
**Alhalabi 2015**	High risk	(-)	(?)	(?)	(-)	(-)	(-)	(?)	(+)
**Bergmann 2017**	Moderate risk	(-)	(+)	(+)	(+)	(+)	(+)	(+)	(?)
**Esquirol 2017**	High risk	(-)	(-)	(+)	(+)	(+)	(+)	(+)	(+)
**Euro 2019**	Moderate risk	(+)	(-)	(+)	(+)	(+)	(+)	(+)	(+)
**Halonen 2019**	Moderate risk	(-)	(+)	(+)	(+)	(+)	(+)	(+)	(?)
**Herin 2014**	High risk	(-)	(?)	(+)	(+)	(+)	(+)	(+)	(+)
**Heuch 2017**	Low risk	(+)	(+)	(+)	(+)	(+)	(+)	(+)	(+)
**Jansen 2004**	Low risk	(+)	(+)	(+)	(+)	(+)	(-)	(+)	(?)
**Jørgensen 2013**	Low risk	(+)	(+)	(+)	(+)	(+)	(-)	(+)	(?)
**Krause 2004**	Low risk	(+)	(+)	(+)	(+)	(+)	(+)	(+)	(?)
**Latza 2002**	High risk	(-)	(-)	(+)	(+)	(+)	(+)	(+)	(-)
**Matsudaira 2019**	High risk	(-)	(-)	(+)	(+)	(-)	(+)	(+)	(?)
**Matsudaira 2015**	High risk	(-)	(+)	(+)	(+)	(-)	(-)	(+)	(?)
**Matsudaira 2014**	Moderate risk	(-)	(+)	(+)	(+)	(+)	(+)	(+)	(+)
**Picavet 2016**	High risk	(-)	(-)	(+)	(?)	(+)	(+)	(+)	(+)
**Prado-Leon 2014**	High risk	(+)	(+)	(+)	(+)	(+)	(-)	(?)	(-)
**Seidler 2011**	Moderate risk	(-)	(+)	(+)	(+)	(+)	(+)	(+)	(-)
**Seidler 2009**	Moderate risk	(-)	(+)	(+)	(+)	(+)	(-)	(-)	(+)
**Seidler 2003**	Moderate risk	(+)	(+)	(+)	(-)	(+)	(+)	(-)	(?)
**Seyedmehdi 2016**	Moderate risk	(+)	(-)	(+)	(+)	(+)	(-)	(+)	(?)
**Sørensen 2001**	Moderate risk	(+)	(-)	(+)	(+)	(+)	(+)	(+)	(+)
**Tubach 2004**	Moderate risk	(+)	(-)	(+)	(+)	(+)	(-)	(+)	(+)
**Vieira 2018**	High risk	(-)	(-)	(+)	(-)	(+)	(+)	(-)	(+)
**Wahlström 2018**	Moderate risk	(+)	(+)	(+)	(?)	(+)	(+)	(+)	(+)

Appendix D. GRADE.

The quality of evidence was evaluated in accordance with the GRADE guidelines. Therefore, the evidence starts at low quality when evaluating observational studies.

Table S7. Quality level.

	**Four levels of evidence**
**Quality level**	**Definition**
High ++++	We are confident that the true effect lies close to that of the estimate of the effect.
Moderate +++	We are moderately confident in the effect estimate: The true effect is likely to be close to the estimate of the effect, but there is a possibility that it is substantially different.
Low ++	Our confidence in the effect estimate is limited: The true effect may be substantially different from the estimate of the effect.
Very low +	We have very little confidence in the effect estimate: The true effect is likely to be substantially different from the estimate of effect.

Table S8. Downgrading and upgrading criteria.

	**Evaluating the quality of evidence**
**Downgrading factors**	**Criteria for downgrading**
Risk of bias	If ≥50% of the included studies were assessed as high risk of bias, this domain was downgraded. In addition, if <50% of the included studies were assessed as high risk of bias, we compared the pooled estimate with the estimate from the sensitivity analysis based on low/moderate risk of bias ratings.
Indirectness	If differences are observed in populations, outcome, or exposure assessments.
Inconsistency	Widely different estimates of effect among studies included in our meta-analysis.
Imprecision	Few studies or few participants with wide 95% confidence intervals of the pooled estimates.
Publication bias	Evidence of funnel plot asymmetry and/or p-value of Egger's test.
**Upgrading factors**	**Criteria for upgrading**
Magnitude of effect	If a large magnitude of effect exists.
Dose response	Assessed either from scatterplots (visualisation) or if included studies provided a test of exposure-response relation.
Residual confounding	Consideration of all plausible confounders increases confidence in the estimated effect. If another domain was evaluated as "serious" plausible confounding was rated as no.

Reasons for our evaluations

Lifting/carrying loads: Was upgraded due to evidence of a possible exposure-response effect but downgraded due to suspected publication bias assessed from funnel plots and Egger's test.

Non-neutral postures: Was upgraded due evidence of a possible exposure-response effect but downgraded due to suspected publication bias assessed from funnel plots and Egger's test.

Whole-body vibrations: Was downgraded due to suspected publication bias assessed from funnel plots and Egger's test.

Standing/walking: Was downgraded based on the risk of bias evaluation since 50 % of the included studies were assessed as high risk of bias. Furthermore, it was downgraded due to inconsistency based on the inconclusive direction of the effect estimates. Two studies showed increased risk, one showed reduced risk, and three showed no effect.

Sitting: Was downgraded due to inconsistency based on the inconclusive direction of the effect estimates. Overall, four studies showed close to no effect, one study showed increased risk, and one study showed a reduced risk.

Combined exposures: Was upgraded due to a large magnitude of effect (OR >2.0) and evidence of a possible exposure-response effect but downgraded due to large confidence intervals of the pooled estimate (imprecision) and differences in populations, exposure, and outcome assessments (indirectness).

Table S9. GRADE assessment based on the 26 included studies.

	Certainty assessment							
**No of studies**	**Study design**	**Risk of Bias**	**Inconsistency**	**Indirectness**	**Imprecision**	**Other considerations**	**Effect estimate (95% CI)**	**Certainty**
**Lifting/carrying loads**								
15	Observational studies	Not serious (0)	Not serious (0)	Not serious (0)	Not serious (0)	- Indication of publication bias (-1)<br>- Plausible confounding (0)<br>- Dose-response gradient (+1)<br>- Magnitude of effect (0)	OR 1.7 (1.4 to 2.2)	⊕⊕⊕◯ Moderate
**Non-neutral postures**								
12	Observational studies	Not serious (0)	Not serious (0)	Not serious (0)	Not serious (0)	- Indication of publication bias (-1)<br>- Plausible confounding (0)<br>- Dose-response gradient (+1)<br>- Magnitude of effect (0)	OR 1.5 (1.2 to 1.9)	⊕⊕⊕◯ Moderate
**Whole-body vibrations**								
7	Observational studies	Not serious (0)	Not serious (0)	Not serious (0)	Not serious (0)	- Indication of publication bias (-1)<br>- Plausible confounding (0)<br>- Dose-response gradient (0)<br>- Magnitude of effect (0)	OR 1.4 (1.1 to 1.7)	⊕⊕◯◯ Low
**Standing/walking**								
6	Observational studies	Serious (-1)	Serious (-1)	Not serious (0)	Not serious (0)	- No indication off publication bias (0)<br>- Plausible confounding (0)<br>- Dose-response gradient (0)<br>- Magnitude of effect (0)	OR 1.0 (0.8 to 1.3)	⊕◯◯◯ Very low
**Sitting**								
6	Observational studies	Not serious (0)	Serious (-1)	Not serious (0)	Not serious (0)	- No indication off publication bias (0)<br>- Plausible confounding (0)<br>- Dose-response gradient (0)<br>- Magnitude of effect (0)	OR 1.2 (1.0 to 1.5)	⊕⊕◯◯ Low
**Combined exposure**								
5	Observational studies	Not serious (0)	Not serious (0)	Serious (-1)	Serious (-1)	- Difficult to evaluate (0)<br>- Plausible confounding (0)<br>- Dose-response gradient (+1)<br>- Magnitude of effect (+1)	OR 2.2 (1.4 to 3.6)	⊕⊕⊕◯ Moderate

Appendix E. Excluded articles.

Table SS 10. Excluded articles based on full-text read and reason for exclusion.

References: Studies published before January 10, 2014		Explanation for exclusion
1	Albert HB, Godskesen M, Korsholm L, Westergaard JG. Risk factors in developing pregnancy-related pelvic girdle pain. Acta Obstet Gynecol Scand. 2006;85(5):539-44.	Outcome criteria not fulfilled.
2	Andersen JH, Haahr JP, Frost P. Risk factors for more severe regional muscul-oskeletal symptoms: a two-year pro-spective study of a general working population. Arthritis Rheum 2007;56:1355-64.	Outcome criteria not fulfilled.
3	Battie MC, Videman T, Gibbons LE, Fisher LD, Manninen H, Gill K. 1995 Volvo Award in clinical sciences. Deter-minants of lumbar disc degeneration. A study relating lifetime exposures and magnetic resonance imaging findings in identical twins. Spine (Phila Pa 1976) 1995;20:2601-12.	Outcome criteria not fulfilled.
4	Battie MC, Videman T, Gibbons LE, Manninen H, Gill K, Pope M, et al. Occupational driving and lumbar disc degeneration: a case-control study. Lancet 2002;360:1369-74.	Outcome criteria not fulfilled.
5	Bergenudd H, Johnell O. Somatic versus nonsomatic shoulder and back pain experience in middle age in relation to body build, physical fitness, bone mineral content, gammaglutamyltransferase, occupational workload, and psychosocial factors. Spine (Phila Pa 1976). 1991;16(9):1051-5.	Outcome criteria not fulfilled.
6	Biering-Sorensen F. A prospective study of low back pain in a general population. I. Occurrence, recurrence and aetiology. Scand J Rehabil Med. 1983;15(2):71-9.	Outcome criteria not fulfilled.
7	Biering-Sorensen F, Thomsen C. Medical, social and occupational history as risk indicators for low-back trouble in a general population. Spine (Phila Pa 1976). 1986;11(7):720-5.	Outcome criteria not fulfilled.
8	Bigos SJ, Battie MC, Spengler DM, Fisher LD, Fordyce WE, Hansson T, et al. A longitudinal, prospective study of industrial back injury reporting. Clinical orthopaedics and related research. 1992(279):21-34.	Outcome criteria not fulfilled.
9	Bigos SJ, Battie MC, Spengler DM, Fisher LD, Fordyce WE, Hansson TH, et al. A prospective study of work perceptions and psychosocial factors affecting the report of back injury. Spine (Phila Pa 1976). 1991;16(1):1-6.	Outcome criteria not fulfilled.
10	Bildt C, Alfredsson L, Michélsen H, Punnett L, Vingård E, Torgén M, et al. Occupational and nonoccupational risk indicators for incident and chronic low back pain in a sample of the Swedish general population during a 4-year period: an influence of depression? International Journal of Behavioral Medicine 2000;7:372-92.	Outcome criteria not fulfilled.
11	Bildt C, Alfredsson L, Punnett L, Theobald H, Torgen M, Wikman A. Effects of drop out in a longitudinal study of musculoskeletal disorders. Occup Environ Med. 2001;58(3):194-9.	Outcome criteria not fulfilled.
12	Bjorksten MG, Talback M. A follow-up study of psychosocial factors and musculoskeletal problems among unskilled female workers with monotonous work. Eur J Public Health. 2001;11(1):102-8.	Outcome criteria not fulfilled.
13	Boos N, Rieder R, Schade V, Spratt KF, Semmer N, Aebi M. 1995 Volvo Award in clinical sciences. The diagnostic accuracy of magnetic resonance imaging, work perception, and psychosocial factors in identifying symptomatic disc herniations. Spine (Phila Pa 1976). 1995;20(24):2613-25.	Outcome criteria not fulfilled.
14	Boos N, Semmer N, Elfering A, Schade V, Gal I, Zanetti M, et al. Natural history of individuals with asymptomatic disc abnormalities in magnetic resonance imaging: predictors of low back pain-related medical consultation and work incapacity. Spine (Phila Pa 1976). 2000;25(12):1484-92.	Outcome criteria not fulfilled.
15	Boshuizen HC, Bongers PM, Hulshof CT. Self-reported back pain in tractor drivers exposed to whole-body vibration. Int Arch Occup Environ Health 1990;62:109-15.	Outcome criteria not fulfilled.
16	Bovenzi M. Metrics of whole-body vibration and exposure-response relationship for low back pain in professional drivers: a prospective cohort study. Int Arch Occup Environ Health 2009;82:893-917.	Outcome criteria not fulfilled.

17	Bovenzi M. A longitudinal study of low back pain and daily vibration exposure in professional drivers. Ind Health 2010;48:584-95.	Outcome criteria not fulfilled.
18	Bridger RS, Brasher K, Bennett A. Sustaining person-environment fit with a changing workforce. Ergonomics. 2012.	Outcome criteria not fulfilled.
19	Brynhildsen J, Hansson A, Persson A, Hammar M. Follow-up of patients with low back pain during pregnancy. Obstet Gynecol. 1998;91(2):182-6.	Outcome criteria not fulfilled.
20	Bugajska J, Zolnierczyk-Zreda D, Jedryka-Goral A, Gasik R, Hildt-Ciupinska K, Malinska M, et al. Psychological factors at work and musculoskeletal disorders: a one year prospective study. Rheumatol Int 2013;33:2975-83.	Outcome criteria not fulfilled.
21	Burdorf A, Jansen JP. Predicting the long term course of low back pain and its consequences for sickness absence and associated work disability. Occup Environ Med 2006;63:522-9.	Outcome criteria not fulfilled.
22	Canivet C, Ostergren PO, Choi B, Nilsson P, af Sillen U, Moghadassi M, et al. Sleeping problems as a risk factor for subsequent musculoskeletal pain and the role of job strain: results from a one-year follow-up of the Malmo Shoulder Neck Study Cohort. International journal of behavioral medicine. 2008;15(4):254-62.	Outcome criteria not fulfilled.
23	Christensen JO, Knardahl S. Work and back pain: a prospective study of psychological, social and mechanical predictors of back pain severity. Eur J Pain 2012;16:921-33.	Outcome criteria not fulfilled.
24	Clausen T, Andersen LL, Holtermann A, Jorgensen AF, Aust B, Rugulies R. Do self-reported psychosocial working conditions predict low back pain after adjustment for both physical work load and depressive symptoms? A prospective study among female eldercare workers. Occup Environ Med 2013; 70:538-44.	Outcome criteria not fulfilled.
25	Clays E, De Bacquer D, Leynen F, Kornitzer M, Kittel F, De Backer G. The impact of psychosocial factors on low back pain: longitudinal results from the Belstress study. Spine (Phila Pa 1976). 2007;32(2):262-8.	Outcome criteria not fulfilled.
26	Coenen P, Kingma I, Boot CR, Twisk JW, Bongers PM, van Dieen JH. Cumulative low back load at work as a risk factor of low back pain: a prospective cohort study. J Occup Rehabil 2013;23:11-8.	Outcome criteria not fulfilled.
27	Coupland CA, Grainge MJ, Cliffe SJ, Hosking DJ, Chilvers CE. Occupational activity and bone mineral density in postmenopausal women in England. Osteoporos Int. 2000;11(4):310-5.	Outcome criteria not fulfilled.
28	Daltroy LH, Larson MG, Wright EA, Malspeis S, Fossel AH, Ryan J, et al. A casecontrol study of risk factors for industrial low back injury: implications for primary and secondary prevention programs. Am J Ind Med. 1991;20(4):505-15.	Outcome criteria not fulfilled.
29	de Zwart BC, Broersen JP, van der Beek AJ, Frings-Dresen MH, Van Dijk FJ. Selection related to musculoskeletal complaints among employees. Occup Environ Med. 1997;54(11):800-6.	Outcome criteria not fulfilled.
30	de Zwart BC, Broersen JP, Frings-Dresen MH, van Dijk FJ. Repeated survey on changes in musculoskeletal complaints relative to age and work demands. Occup Environ Med. 1997;54(11):793-9.	Outcome criteria not fulfilled.
31	Elders LA, Burdorf A. Prevalence, incidence, and recurrence of low back pain in scaffolders during a 3-year follow-up study. Spine (Phila Pa 1976) 2004;29:E101-6.	Outcome criteria not fulfilled.
32	Elfering A, Grebner S, Semmer NK, Gerber H. Time control, catecholamines and back pain among young nurses. Scand J Work Environ Health 2002;28:386-93.	Outcome criteria not fulfilled.

33	Elfering A, Mannion AF, Jacobshagen N, Tamcan O, Muller U. Beliefs about back pain predict the recovery rate over 52 consecutive weeks. Scand J Work Environ Health. 2009;35(6):437-45.	Outcome criteria not fulfilled.
34	Elfering A, Semmer N, Birkhofer D, Zanetti M, Hodler J, Boos N. Risk factors for lumbar disc degeneration: a 5-year prospective MRI study in asymptomatic individuals. Spine (Phila Pa 1976). 2002;27(2):125-34.	No measure of an association.
35	Elfering A, Semmer NK, Schade V, Grund S, Boos N. Supportive colleague, unsupportive supervisor: the role of provider-specific constellations of social support at work in the development of low back pain. Journal of occupational health psychology. 2002;7(2):130-40.	Outcome criteria not fulfilled.
36	Engkvist IL. Back injuries among nurses - A comparison of the accident processes after a 10-year follow-up. Safety Science. 2008;46(2):291-301.	Outcome criteria not fulfilled.
37	Engkvist IL, Hagberg M, Hjelm EW, Menckel E, Ekenvall L. The accident process preceding overexertion back injuries in nursing personnel. PROSA study group. Scand J Work Environ Health. 1998;24(5):367-75.	Outcome criteria not fulfilled.
38	Engkvist IL, Hjelm EW, Hagberg M, Menckel E, Ekenvall L. Risk indicators for reported over-exertion back injuries among female nursing personnel. Epidemiology (Cambridge, Mass). 2000;11(5):519-22.	Outcome criteria not fulfilled.
39	Eriksen W, Natvig B, Bruusgaard D. Smoking, heavy physical work and low back pain: a four-year prospective study. Occup Med (Lond). 1999;49(3):155-60.	Outcome criteria not fulfilled.
40	Eriksen W, Bruusgaard D, Knardahl S. Work factors as predictors of intense or disabling low back pain; a prospective study of nurses’ aides. Occup Environ Med 2004;61:398-404.	Outcome criteria not fulfilled.
41	Ferguson SA, Allread WG, Burr DL, Heaney C, Marras WS. Biomechanical, psychosocial and individual risk factors predicting low back functional impairment among furniture distribution employees. Clinical Biomechanics. 2012;27(2):117-23.	Outcome criteria not fulfilled.
42	Ferguson SA, Marras WS, Burr DL. The influence of individual low back health status on workplace trunk kinematics and risk of low back disorder. Ergonomics. 2004;47(11):1226-37.	Outcome criteria not fulfilled.
43	Feyer AM, Herbison P, Williamson AM, de Silva I, Mandryk J, Hendrie L, et al. The role of physical and psycho-logical factors in occupational low back pain: a prospective cohort study. Occup Environ Med 2000;57:116-20.	Outcome criteria not fulfilled.
44	Gatchel RJ, Polatin PB, Mayer TG. The dominant role of psychosocial risk factors in the development of chronic low back pain disability. Spine (Phila Pa 1976). 1995;20(24):2702-9.	Outcome criteria not fulfilled.
45	Gheldof EL, Vinck J, Vlaeyen JW, Hidding A, Crombez G. Development of and recovery from short- and long-term low back pain in occupational settings: a prospective cohort study. Eur J Pain 2007;11:841-54.	Outcome criteria not fulfilled.
46	Gnudi S, Sitta E, Gnudi F, Pignotti E. Relationship of a lifelong physical workload with physical function and low back pain in retired women. Aging Clinical & Experimental Research. 2009;21(1):55-61.	Outcome criteria not fulfilled.
47	Gonge H, Jensen LD, Bonde JP. Do psychosocial strain and physical exertion predict onset of low-back pain among nursing aides? Scand J Work Environ Health 2001;27:388-94.	Outcome criteria not fulfilled.
48	Gonge H, Jensen LD, Bonde JP. Are psychosocial factors associated with low-back pain among nursing personnel? Work & Stress 2002;16:79-87.	Outcome criteria not fulfilled.

49	Hagberg M, Vilhemsson R, Tornqvist EW, Toomingas A. Incidence of self-reported reduced productivity owing to musculoskeletal symptoms: association with workplace and individual factors among computer users. Ergonomics 2007;50:1820-34.	Outcome criteria not fulfilled.
50	Hakkanen M, Viikari-Juntura E, Martikainen R. Incidence of musculo-skeletal disorders among newly em-ployed manufacturing workers. Scand J Work Environ Health 2001;27:381-7.	Outcome criteria not fulfilled.
51	Hamberg-van Reenen HH, Ariens GA, Blatter BM, van der Beek AJ, Twisk JW, van Mechelen W, et al. Is an imbalance between physical capacity and exposure to work-related physical factors associated with low-back, neck or shoulder pain? Scand J Work Environ Health 2006;32:190-7.	Outcome criteria not fulfilled.
52	Harkness EF, Macfarlane GJ, Nahit ES, Silman AJ, McBeth J. Risk factors for new-onset low back pain amongst cohorts of newly employed workers. Rheumatology (Oxford) 2003;42: 959-68.	Outcome criteria not fulfilled.
53	Hartvigsen J, Bakketeig LS, Leboeuf- Yde C, Engberg M, Lauritzen T. The association between physical workload and low back pain clouded by the ”healthy worker” effect: population-based cross-sectional and 5-year pro-spective questionnaire study. Spine (Phila Pa 1976) 2001;26:1788-92; discussion 1792-3.	Outcome criteria not fulfilled.
54	Hartvigsen J, Bakketeig LS, Leboeuf-Yde C, Engberg M, Lauritzen T. [The association between physical workload and low back pain clouded by the "healthy worker" effect]. Ugeskr Laeger. 2002;164(21):2765-8.	Outcome criteria not fulfilled.
55	Hellsing A, Bryngelsson I. Predictors of musculoskeletal pain in men: a twenty-year follow-up from examination at enlistment. Spine (Phila Pa 1976). 2000;25(23):3080-6.	Outcome criteria not fulfilled.
56	Holmberg S, Thelin A, Stiernstrom EL, Svardsudd K. The impact of physical work exposure on musculoskeletal symptoms among farmers and rural non-farmers. A population-based study. Annals of Agricultural and Environmental Medicine. 2003;10(2):179-84.	Outcome criteria not fulfilled.
57	Holmberg S, Thelin A, Stiernstrom EL, Svardsudd K. Low back pain comorbidity among male farmers and rural referents: a population-based study. Ann Agric Environ Med. 2005;12(2):261-8.	Outcome criteria not fulfilled.
58	Holtermann A, Blangsted AK, Hansen K, Christensen H, Sogaard K. What characterizes cleaners sustaining good musculoskeletal health after years with physically heavy work? International Archives of Occupational and Environmental Health. 2009;82(8):1015-22.	Outcome criteria not fulfilled.
59	Holtermann A, Clausen T, Aust B, Mortensen OS, Andersen LL. Risk for low back pain from different frequencies, load mass and trunk postures of lifting and carrying among female healthcare workers. Int Arch Occup Environ Health. 2012.	Outcome criteria not fulfilled.
60	Holtermann A, Clausen T, Aust B, Mortensen OS, Andersen LL. Does occupational lifting and carrying among female health care workers contribute to an escalation of pain-day frequency? Eur J Pain. 2013;17(2):290-6.	Outcome criteria not fulfilled.
61	Holtermann A, Clausen T, Jørgensen MB, Burdorf A, Andersen LL. Patient handling and risk for developing persistent low-back pain among female healthcare workers. Scand J Work Environ Health 2013;39:164-9.	Outcome criteria not fulfilled.
62	Hooftman WE, van der Beek AJ, Bongers PM, van Mechelen W. Is there a gender difference in the effect of work-related physical and psycho-social risk factors on musculoskeletal symptoms and related sickness absence? Scand J Work Environ Health 2009;35:85-95.	Outcome criteria not fulfilled.
63	Hoogendoorn WE, Bongers PM, de Vet HC, Douwes M, Koes BW, Miedema MC, et al. Flexion and rotation of the trunk and lifting at work are risk factors for low back pain: results of a prospective cohort study. Spine (Phila Pa 1976) 2000;25:3087-92.	Outcome criteria not fulfilled.
64	Hoogendoorn WE, Bongers PM, de Vet HC, Houtman IL, Ariens GA, van Mechelen W, et al. Psychosocial work characteristics and psychological strain in relation to low-back pain. Scand J Work Environ Health 2001;27:258-67.	Outcome criteria not fulfilled.
65	Hoogendoorn WE, Bongers PM, de Vet HC, Twisk JW, van Mechelen W, Bouter LM. Comparison of two different approaches for the analysis of data from a prospective cohort study: an application to work related risk factors for low back pain. Occup Environ Med 2002;59:459-65.	Outcome criteria not fulfilled.

66	Hoozemans MJ, van der Beek AJ, Fring-Dresen MH, van der Woude LH, van Dijk FJ. Low-back and shoulder complaints among workers with pushing and pulling tasks. Scand J Work Environ Health 2002;28:293-303.	Outcome criteria not fulfilled.
67	Hultman G, Nordin M, Saraste H. Physical and psychological workload in men with and without low back pain. Scand J Rehabil Med 1995;27:11-7.	Study design criteria not fulfilled.
68	Hägg O, Fritzell P, Nordwall A. Characteristics of patients with chronic low back pain selected for surgery: a comparison with the general population reported from the Swedish Lumbar Spine Study including commentary by Fanuele J. Spine (Phila Pa 1976). 2002;27(11):1223-31.	Exposure criteria not fulfilled.
69	Jensen A, Kaerlev L, Tuchsen F, Hannerz H, Dahl S, Nielsen PS, et al. Locomotor diseases among male long-haul truck drivers and other professional drivers. Int Arch Occup Environ Health. 2008;81(7):821-7.	Outcome criteria not fulfilled.
70	Jensen JC, Haahr JP, Frost P, Andersen JH. Do work-related factors affect care-seeking in general practice for back pain or upper extremity pain? Int Arch Occup Environ Health. 2012.	Outcome criteria not fulfilled.
71	Jensen JN, Holtermann A, Clausen T, Mortensen OS, Carneiro IG, Andersen LL. The greatest risk for low-back pain among newly educated female health care workers; body weight or physical work load? BMC Musculoskelet Disord 2012;13:87.	Outcome criteria not fulfilled.
72	Johnston JM, Landsittel DP, Nelson NA, Gardner LI, Wassell JT. Stressful psychosocial work environment in-creases risk for back pain among retail material handlers. Am J Ind Med 2003;43:179-87.	Outcome criteria not fulfilled.
73	Jorgensen MB, Nabe-Nielsen K, Clausen T, Holtermann A. Independent effect of physical workload and childhood socioeconomic status on low back pain among health care workers in Denmark. Spine (Phila Pa 1976) 2013;38:E359-66.	Outcome criteria not fulfilled.
74	Josephson M, Ahlberg G, Harenstam A, Svensson H, Theorell T, Wiktorin C, et al. Paid and unpaid work, and its relation to low back and neck/shoulder disorders among women. Women & health. 2003;37(2):17-30.	Outcome criteria not fulfilled.
75	Josephson M, Hagberg M, Hjelm EW. Self-reported physical exertion in geriatric care. A risk indicator for low back symptoms? Spine (Phila Pa 1976) 1996;21:2781-5.	Outcome criteria not fulfilled.
76	Josephson M, Lagerstrom M, Hagberg M, Wigaeus Hjelm E. Musculoskeletal symptoms and job strain among nursing personnel: a study over a three year period. Occup Environ Med. 1997;54(9):681-5.	Outcome criteria not fulfilled.
77	Josephson M, Vingard E. Workplace factors and care seeking for low-back pain among female nursing personnel. MUSIC-Norrtalje Study Group. Scand J Work Environ Health 1998;24: 465-72.	Outcome criteria not fulfilled.
78	Juhl M, Andersen PK, Olsen J, Andersen AM. Psychosocial and physical work environment, and risk of pelvic pain in pregnancy. A study within the Danish national birth cohort. J Epidemiol Community Health. 2005;59(7):580-5.	Outcome criteria not fulfilled.
79	Juul-Kristensen B, Sogaard K, Stroyer J, Jensen C. Computer users' risk factors for developing shoulder, elbow and back symptoms. Scand J Work Environ Health 2004;30:390-8.	Outcome criteria not fulfilled.
80	Kaaria SM, Malkia EA, Luukkonen RA, Leino-Arjas PI. Pain and clinical findings in the low back: a study of industrial employees with 5-, 10-, and 28-year follow-ups. Eur J Pain. 2010;14(7):759-63.	Outcome criteria not fulfilled.
81	Kaaria S, Leino-Arjas P, Rahkonen O, Lahti J, Lahelma E, Laaksonen M. Risk factors of sciatic pain: a prospective study among middle-aged employees. Eur J Pain 2011;15:584-90.	Study design criteria not fulfilled.

82	Kaila-Kangas L, Kivimaki M, Riihimaki H, Luukkonen R, Kirjonen J, Leino-Arjas P. Psychosocial factors at work as predictors of hospitalization for back disorders: a 28-year follow-up of industrial employees. Spine (Phila Pa 1976) 2004;29:1823-30.	Outcome criteria not fulfilled.
83	Keeney BJ, Turner JA, Fulton-Kehoe D, Wickizer TM, Chan KC, Franklin GM. Early predictors of occupational back reinjury: results from a prospective study of workers in Washington State. Spine (Phila Pa 1976). 2013;38(2):178-87.	Outcome criteria not fulfilled.
84	Kelsey JL, Githens PB, White AA, 3rd, Holford TR, Walter SD, O'Connor T, et al. An epidemiologic study of lifting and twisting on the job and risk for acute prolapsed lumbar intervertebral disc. Journal of orthopaedic research : official publication of the Orthopaedic Research Society. 1984;2(1):61-6.	Outcome criteria not fulfilled.
85	Kerr MS, Frank JW, Shannon HS, Norman RW, Wells RP, Neumann WP, et al. Biomechanical and psycho-social risk factors for low back pain at work. Am J Public Health 2001;91: 1069-75.	Outcome criteria not fulfilled.
86	Kim IH, Geiger-Brown J, Trinkoff A, Muntaner C. Physically demanding workloads and the risks of musculo-skeletal disorders in homecare workers in the USA. Health Soc Care Community 2010;18:445-55.	Outcome criteria not fulfilled.
87	Kishi R, Doi R, Fukuchi Y, Satoh H, Satoh T, Ono A, et al. Subjective symptoms and neurobehavioral performances of ex-mercury miners at an average of 18 years after the cessation of chronic exposure to mercury vapor. Mercury Workers Study Group. Environmental research. 1993;62(2):289-302.	Outcome criteria not fulfilled.
88	Knox JB, Orchowski JR, Scher DL, Owens BD, Burks R, Belmont PJ, Jr. Occupational driving as a risk factor for low back pain in active-duty military service members. Spine J. 2013.	Outcome criteria not fulfilled.
89	Kopec JA, Sayre EC. Work-related psychosocial factors and chronic pain: a prospective cohort study in Canadian workers. J Occup Environ Med 2004; 46:1263-71.	Outcome criteria not fulfilled.
90	Kopec JA, Sayre EC, Esdaile JM. Predictors of back pain in a general population cohort. Spine (Phila Pa 1976) 2003;29:70-7; discussion 77-8.	Outcome criteria not fulfilled.
91	Kraus JF, Schaffer KB, McArthur DL, Peek-Asa C. Epidemiology of acute low back injury in employees of a large home improvement retail company. Am J Epidemiol 1997;146:637-45.	Outcome criteria not fulfilled.
92	Krause N, Ragland DR, Fisher JM, Syme SL. Psychosocial job factors, physical workload, and incidence of work-related spinal injury: a 5-year prospective study of urban transit operators. Spine (Phila Pa 1976) 1998;23:2507-16.	Outcome criteria not fulfilled.
93	Kucera KL, Loomis D, Lipscomb HJ, Marshall SW, Mirka GA, Daniels JL. Ergonomic risk factors for low back pain in North Carolina crab pot and gill net commercial fishermen. Am J Ind Med 2009;52:311-21.	Outcome criteria not fulfilled.
94	Kuh DJ, Coggan D, Mann S, Cooper C, Yusuf E. Height, occupation and back pain in a national prospective study. Br J Rheumatol. 1993;32(10):911-6.	No measure of an association.
95	Kuijer PP, van der Beek AJ, van Dieen JH, Visser B, Frings-Dresen MH. Effect of job rotation on need for recovery, musculoskeletal complaints, and sick leave due to musculoskeletal complaints: a prospective study among refuse collectors. Am J Ind Med. 2005;47(5):394-402.	Outcome criteria not fulfilled.
96	Kujala UM, Taimela S, Viljanen T, Jutila H, Viitasalo JT, Videman T, et al. Physical loading and performance as predictors of back pain in healthy adults. A 5-year prospective study. Eur J Appl Physiol Occup Physiol 1996;73:452-8.	Outcome criteria not fulfilled.
97	Lapointe J, Dionne CE, Brisson C, Montreuil S. Interaction between postural risk factors and job strain on self-reported musculoskeletal symptoms among users of video display units: a three-year prospective study. Scand J Work Environ Health 2009;35:134-44.	Outcome criteria not fulfilled.

98	Larsman P, Johansson Hanse J. The impact of decision latitude, psychological load and social support at work on the development of neck, shoulder and low back symptoms among female human service organization workers. International Journal of Industrial Ergonomics 2009;39:442-6.	Outcome criteria not fulfilled.
99	Latza U, Karmaus W, Sturmer T, Steiner M, Neth A, Rehder U. Cohort study of occupational risk factors of low back pain in construction workers. Occup Environ Med 2000;57:28-34.	Outcome criteria not fulfilled.
100	Lau B, Knardahl S. Perceived job insecurity, job predictability, personality, and health. J Occup Environ Med. 2008;50(2):172-81.	Outcome criteria not fulfilled.
101	Leclerc A, Tubach F, Landre MF, Ozguler A. Personal and occupational predictors of sciatica in the GAZEL cohort. Occup Med (Lond) 2003;53:384-91.	Outcome criteria not fulfilled.
102	Leino PI, Hanninen V. Psychosocial factors at work in relation to back and limb disorders. Scand J Work Environ Health 1995;21:134-42.	Outcome criteria not fulfilled.
103	Lin MY, Ahern JE, Gershon RR, Grimes M. The use of total quality improvement techniques to determine risk factors for back injuries in hospital workers. Clinical performance and quality health care. 1998;6(1):23-7.	Outcome criteria not fulfilled.
104	Linton SJ. Do psychological factors increase the risk for back pain in the general population in both a cross-sectional and prospective analysis? Eur J Pain 2005;9:355-61.	Outcome criteria not fulfilled.
105	Lipscomb HJ, Loomis D, McDonald MA, Kucera K, Marshall S, Li L. Musculoskeletal symptoms among commercial fishers in North Carolina. Appl Ergon. 2004;35(5):417- 26.	Outcome criteria not fulfilled.
106	Lonnberg F, Pedersen PA, Siersma V. Early predictors of the long-term outcome of low back pain--results of a 22-year prospective cohort study. Family practice. 2010;27(6):609-14.	Outcome criteria not fulfilled.
107	Lotters F, Burdorf A, Kuiper J, Miedema H. Model for the work-relatedness of lowback pain. Scand J Work Environ Health. 2003;29(6):431-40.	Study design criteria not fulfilled.
108	Macfarlane GJ, Thomas E, Papageorgiou AC, Croft PR, Jayson MI, Silman AJ. Employment and physical work activities as predictors of future low back pain. Spine (Phila Pa 1976) 1997;22:1143-9.	Outcome criteria not fulfilled.
109	Manninen P, Heliovaara M, Riihimakl H, Makela P. Does psychological distress predict disability? Int J Epidemiol. 1997;26(5):1063-70.	Outcome criteria not fulfilled.
110	Manninen P, Riihimak H, Heliovaara M. Incidence and risk factors of low-back pain in middle-aged farmers. Occup Med (Lond) 1995;45:141-6.	Outcome criteria not fulfilled.
111	Marras WS, Lavender SA, Ferguson SA, Splittstoesser RE, Yang G. Quantitative dynamic measures of physical exposure predict low back functional impairment. Spine (Phila Pa 1976). 2010;35(8):914-23.	Outcome criteria not fulfilled.
112	Massaccesi M, Pagnotta A, Soccetti A, Masali M, Masiero C, Greco F. Investigation of work-related disorders in truck drivers using RULA method. Appl Ergon. 2003;34(4):303-7.	Outcome criteria not fulfilled.
113	Masset DF, Piette AG, Malchaire JB. Relation between functional characteristics of the trunk and the occurrence of low back pain. Associated risk factors. Spine (Phila Pa 1976). 1998;23(3):359-65.	Exposure criteria not fulfilled.

114	Matsudaira K, Kawaguchi M, Isomura T, Arisaka M, Fujii T, Takeshita K, et al. Identification of risk factors for new-onset sciatica in Japanese workers: findings from the Japan epidemiological research of occupation-related back pain study. Spine (Phila Pa 1976) 2013;38:E1691-700.	Outcome criteria not fulfilled.
115	Matsudaira K, Konishi H, Miyoshi K, Isomura T, Takeshita K, Hara N, et al. Potential risk factors for new onset of back pain disability in Japanese workers: findings from the Japan epidemi-ological research of occupation-related back pain study. Spine (Phila Pa 1976) 2012;37:1324-33.	Outcome criteria not fulfilled.
116	Melloh M, Elfering A, Chapple CM, Kaser A, Rolli Salathe C, Barz T, et al. Prognostic occupational factors for persistent low back pain in primary care. Int Arch Occup Environ Health 2013;86:261-9. Epub 2012 Mar 21.	Exposure criteria not fulfilled.
117	Melloh M, Elfering A, Stanton TR, Kaser A, Salathe CR, Barz T, et al. Who is likely to develop persistent low back pain? A longitudinal analysis of prognostic occupational factors. Work. 2013;46(3):297-311.	Exposure criteria not fulfilled.
118	Melloh M, Salathe CR, Elfering A, Kaser A, Barz T, Aghayev E, et al. Occupational, personal and psychosocial resources for preventing persistent low back pain. Int J Occup Saf Ergon. 2013;19(1):29-40.	Exposure criteria not fulfilled.
119	Messing K, Stock SR, Tissot F. Should studies of risk factors for musculoskeletal disorders be stratified by gender? Lessons from the 1998 Quebec Health and Social Survey. Scand J Work Environ Health. 2009;35(2):96-112.	Study design criteria not fulfilled.
120	Miedema HS, Chorus AM, Wevers CW, van der Linden S. Chronicity of back problems during working life. Spine (Phila Pa 1976). 1998;23(18):2021-8; discussion 8-9.	Outcome criteria not fulfilled.
121	Mikkonen P, Viikari-Juntura E, Remes J, Pienimäki T, Solovieva S, Taimela S, et al. Physical workload and risk of low back pain in adolescence. Occup Environ Med 2012;69:284-90.	Outcome criteria not fulfilled.
122	Milosavljevic S, Bagheri N, Vasiljev RM, McBride DI, Rehn B. Does daily exposure to whole-body vibration and mechanical shock relate to the prevalence of low back and neck pain in a rural workforce? Ann Occup Hyg. 2012;56(1):10-7.	Outcome criteria not fulfilled.
123	Miranda H, Viikari-Juntura E, Martikainen R, Takala EP, Riihimaki H. Individual factors, occupational loading, and physical exercise as pre-dictors of sciatic pain. Spine (Phila Pa 1976) 2002;27:1102-9.	Outcome criteria not fulfilled.
124	Miranda H, Viikari-Juntura E, Punnett L, Riihimaki H. Occupational loading, health behavior and sleep disturbance as predictors of low-back pain. Scand J Work Environ Health 2008;34:411-9.	Outcome criteria not fulfilled.
125	Miwa S, Yokogawa A, Kobayashi T, Nishimura T, Igarashi K, Inatani H, et al. Risk factors of recurrent lumbar disc herniation: A single center study and review of the literature. Journal of Spinal Disorders and Techniques. 2012;14.	Outcome criteria not fulfilled.
126	Myers AH, Baker SP, Li G, Smith GS, Wiker S, Liang KY, et al. Back injury in municipal workers: a case-control study. Am J Public Health 1999;89: 1036-41.	Outcome criteria not fulfilled.
127	Nahit ES, Hunt IM, Lunt M, Dunn G, Silman AJ, Macfarlane GJ. Effects of psychosocial and individual psychological factors on the onset of musculoskeletal pain: common and site-specific effects. Ann Rheum Dis 2003;62:755-60.	Outcome criteria not fulfilled.
128	Neumann WP, Wells RP, Norman RW, Frank J, Shannon H, Kerr MS. A posture and load sampling approach to determining low-back pain risk in occupational settings. International Journal of Industrial Ergonomics 2001;27:65-77.	Outcome criteria not fulfilled.
129	Nuwayhid IA, Stewart W, Johnson JV. Work activities and the onset of first-time low back pain among New York City fire fighters. Am J Epidemiol. 1993;137(5):539-48.	Outcome criteria not fulfilled.

130	Nyman T, Mulder M, Iliadou A, Svartengren M, Wiktorin C. Physical workload, low back pain and neck-shoulder pain: a Swedish twin study. Occupational and Environmental Medicine [Internet]. 2009; (6):[395-401 pp.].	No measure of an association.
131	Oleske DM, Lavender SA, Andersson GB, Morrissey MJ, Zold-Kilbourn P, Allen C, et al. Risk factors for recurrent episodes of work-related low back dis-orders in an industrial population. Spine (Phila Pa 1976) 2006;31:789-98.	Outcome criteria not fulfilled.
132	Palmer KT, Griffin M, Ntani G, Shambrook J, McNee P, Sampson M, et al. Professional driving and prolapsed lumbar intervertebral disc diagnosed by magnetic resonance imaging: a case-control study. Scand J Work Environ Health. 2012;38(6):577-81.	Outcome criteria not fulfilled.
133	Palmer KT, Harris CE, Griffin MJ, Bennett J, Reading I, Sampson M, et al. Casecontrol study of low-back pain referred for magnetic resonance imaging, with special focus on whole-body vibration. Scand J Work Environ Health. 2008;34(5):364-73.	Outcome criteria not fulfilled.
134	Papageorgiou AC, Croft PR, Thomas E, Silman AJ, Macfarlane GJ. Psychosocial risks for low back pain: are these related to work? Ann Rheum Dis. 1998;57(8):500-2.	Outcome criteria not fulfilled.
135	Papageorgiou AC, Macfarlane GJ, Thomas E, Croft PR, Jayson MI, Silman AJ. Psychosocial factors in the workplace – do they predict new episodes of low back pain? Evidence from the South Manchester Back Pain Study. Spine (Phila Pa 1976) 1997;22:1137-42.	Outcome criteria not fulfilled.
136	Pietri F, Leclerc A, Boitel L, Chastang JF, Morcet JF, Blondet M. Low-back pain in commercial travelers. Scand J Work Environ Health 1992;18:52-8.	Outcome criteria not fulfilled.
137	Piterman L, Dunt D. Occupational lower-back injuries in a primary medical care setting: a five-year follow-up study. Med J Aust. 1987;147(6):276-9.	Outcome criteria not fulfilled.
138	Plouvier S, Leclerc A, Chastang JF, Bonenfant S, Goldberg M. Socioeconomic position and low-back pain--the role of biomechanical strains and psychosocial work factors in the GAZEL cohort. Scand J Work Environ Health. 2009;35(6):429-36.	Outcome criteria not fulfilled.
139	Plouvier S, Renahy E, Chastang JF, Bonenfant S, Leclerc A. Biomechanical strains and low back disorders: quantifying the effects of the number of years of exposure on various types of pain. Occup Environ Med. 2008;65(4):268-74.	Outcome criteria not fulfilled.
140	Power C, Frank J, Hertzman C, Schierhout G, Li L. Predictors of low back pain onset in a prospective British study. Am J Public Health. 2001;91(10):1671-8.	Outcome criteria not fulfilled.
141	Punnett L, Fine LJ, Keyserling WM, Herrin GD, Chaffin DB. Back dis-orders and nonneutral trunk postures of automobile assembly workers. Scand J Work Environ Health 1991;17:337-46.	Outcome criteria not fulfilled.
142	Ramond-Roquin A, Bodin J, Serazin C, Parot-Schinkel E, Ha C, Richard I, et al. Biomechanical constraints remain major risk factors for low back pain. Results from a prospective cohort study in French male employees. Spine J 2013 Jul 12 [Epub ahead of print].	Outcome criteria not fulfilled.
143	Reigo T, Tropp H, Timpka T. Absence of back disorders in adults and work-related predictive factors in a 5-year perspective. Eur Spine J. 2001;10(3):215-20; discussion 21.	Outcome criteria not fulfilled.
144	Reme SE, Shaw WS, Steenstra IA, Woiszwillo MJ, Pransky G, Linton SJ. Distressed, immobilized, or lacking employer support? A sub-classification of acute work-related low back pain. J Occup Rehabil 2012;22:541-52.	Outcome criteria not fulfilled.
145	Riihimaki H, Viikari-Juntura E, Moneta G, Kuha J, Videman T, Tola S. Incidence of sciatic pain among men in machine operating, dynamic physical work, and sedentary work. A three-year follow-up. Spine (Phila Pa 1976). 1994;19(2):138-42.	Exposure criteria not fulfilled.
146	Riihimaki H, Wickstrom G, Hanninen K, Luopajarvi T. Predictors of sciatic pain among concrete reinforcement workers and house painters--a five-year follow-up. Scand J Work Environ Health. 1989;15(6):415-23.	Outcome criteria not fulfilled.

147	Riyazi N, Rosendaal FR, Slagboom E, Kroon HM, Breedveld FC, Kloppenburg M. Risk factors in familial osteoarthritis: the GARP sibling study. Osteoarthritis Cartilage. 2008;16(6):654-9.	Outcome criteria not fulfilled.
148	Rohrer MH, Santos-Eggimann B, Paccaud F, Haller-Maslov E. Epidemiologic study of low back pain in 1398 Swiss conscripts between 1985 and 1992. Eur Spine J. 1994;3(1):2-7.	No measure of an association.
149	Roy TC, Lopez HP, Piva SR. Loads Worn by Soldiers Predict Episodes of Low Back Pain during Deployment to Afghanistan. Spine (Phila Pa 1976). 2013.	Outcome criteria not fulfilled.
150	Rugulies R, Krause N. Effort-reward imbalance and incidence of low back and neck injuries in San Francisco transit operators. Occup Environ Med. 2008;65(8):525-33.	Exposure criteria not fulfilled.
151	Rugulies R, Krause N. Job strain, iso-strain, and the incidence of low back and neck injuries. A 7.5-year prospective study of San Francisco transit operators. Soc Sci Med 2005;61:27-39.	Outcome criteria not fulfilled.
152	Shannon HS, Woodward CA, Cunningham CE, McIntosh J, Lendrum B, Brown J, et al. Changes in general health and musculoskeletal outcomes in the workforce of a hospital undergoing rapid change: a longitudinal study. J Occup Health Psychol 2001;6:3-14.	Outcome criteria not fulfilled.
153	Shaw WS, Pransky G, Winters T. The Back Disability Risk Questionnaire for work-related, acute back pain: prediction of unresolved problems at 3-month follow-up. J Occup Environ Med 2009;51:185-94.	Outcome criteria not fulfilled.
154	Smedley J, Egger P, Cooper C, Coggon D. Prospective cohort study of predictors of incident low back pain in nurses. BMJ 1997;314:1225-8.	Outcome criteria not fulfilled.
155	Sterud T, Tynes T. Work-related psychosocial and mechanical risk factors for low back pain: a 3-year follow-up study of the general working population in Norway. Occup Environ Med 2013;70:296-302.	Outcome criteria not fulfilled.
156	Stevenson JM, Weber CL, Smith JT, Dumas GA, Albert WJ. A longitudinal study of the development of low back pain in an industrial population. Spine (Phila Pa 1976). 2001;26(12):1370-7.	Exposure criteria not fulfilled.
157	Stobbe TJ, Plummer RW, Jensen RC, Attfield MD. Incidence of low back injuries among nursing personnel as a function of patient lifting frequency. Journal of Safety Research 1988;19:21-8.	Study design criteria not fulfilled.
158	Stomp-van den Berg SG, Hendriksen IJ, Bruinvels DJ, Twisk JW, van Mechelen W, van Poppel MN. Predictors for post-partum pelvic girdle pain in working women: the Mom@Work cohort study. Pain 2012;153:2370-9.	Outcome criteria not fulfilled.
159	Studnek JR, Crawford JM. Factors associated with back problems among emergency medical technicians. Am J Ind Med 2007;50:464-9.	Outcome criteria not fulfilled.
160	Thorbjornsson CB, Alfredsson L, Fredriksson K, Michelsen H, Punnett L, Vingard E, et al. Physical and psycho-social factors related to low back pain during a 24-year period. A nested case-control analysis. Spine (Phila Pa 1976) 2000;25:369-74; discussion 375.	Outcome criteria not fulfilled.
161	Tiemessen IJ, Hulshof CT, Frings-Dresen MH. Low back pain in drivers exposed to whole body vibration: ana-lysis of a dose-response pattern. Occup Environ Med 2008;65:667-75.	Outcome criteria not fulfilled.
162	Torp S, Riise T, Moen BE. The impact of psychosocial work factors on musculo-skeletal pain: a prospective study. J Occup Environ Med 2001;43:120-6.	Outcome criteria not fulfilled.
163	Trinkoff AM, Le R, Geiger-Brown J, Lipscomb J, Lang G. Longitudinal relationship of work hours, mandatory overtime, and on-call to musculoskeletal problems in nurses. Am J Ind Med 2006;49:964-71.	Outcome criteria not fulfilled.

164	Tubach F, Leclerc A, Landre MF, Pietri- Taleb F. Risk factors for sick leave due to low back pain: a prospective study. J Occup Environ Med 2002;44:451-8.	Outcome criteria not fulfilled.
165	van den Heuvel SG, Ariens GA, Boshuizen HC, Hoogendoorn WE, Bongers PM. Prognostic factors related to recurrent low-back pain and sickness absence. Scand J Work Environ Health 2004;30:459-67.	Outcome criteria not fulfilled.
166	Van Nieuwenhuyse A, Somville PR, Crombez G, Burdorf A, Verbeke G, Johannik K, et al. The role of physical workload and pain related fear in the development of low back pain in young workers: evidence from the BelCoBack Study; results after one year of follow up. Occup Environ Med 2006;63:45-52.	Outcome criteria not fulfilled.
167	van Poppel MN, Koes BW, Deville W, Smid T, Bouter LM. Risk factors for back pain incidence in industry: a prospective study. Pain 1998;77:81-6.	Outcome criteria not fulfilled.
168	Vandergrift JL, Gold JE, Hanlon A, Punnett L. Physical and psychosocial ergonomic risk factors for low back pain in automobile manufacturing workers. Occup Environ Med 2012; 69:29-34. Epub 2011 May 17.	Outcome criteria not fulfilled.
169	Ward MM, Reveille JD, Learch TJ, Davis JC, Jr., Weisman MH. Occupational physical activities and long-term functional and radiographic outcomes in patients with ankylosing spondylitis. Arthritis Rheum 2008;59:822-32.	Outcome criteria not fulfilled.
170	Venning PJ, Walter SD, Stitt LW. Personal and job-related factors as determinants of incidence of back injuries among nursing personnel. J Occup Med 1987;29:820-5.	Outcome criteria not fulfilled.
171	Verbeek JH, van der Beek AJ. Psychosocial factors at work and back pain: a prospective study in office workers. Int J Occup Med Environ Health 1999;12:29-39.	Outcome criteria not fulfilled.
172	Wergeland EL, Veiersted B, Ingre M, Olsson B, Akerstedt T, Bjornskau T, et al. A shorter workday as a means of reducing the occurrence of musculo-skeletal disorders. Scand J Work Environ Health 2003;29:27-34.	Outcome criteria not fulfilled.
173	Wickstrom GJ, Pentti J. Occupational factors affecting sick leave attributed to low-back pain. Scand J Work Environ Health 1998;24:145-52.	Outcome criteria not fulfilled.
174	Videman T, Battie MC, Parent E, Gibbons LE, Vainio P, Kaprio J. Progression and determinants of quantitative magnetic resonance imaging measures of lumbar disc degeneration: a five-year follow-up of adult male monozygotic twins. Spine (Phila Pa 1976) 2008;33:1484-90.	Outcome criteria not fulfilled.
175	Videman T, Ojajarvi A, Riihimaki H, Troup JD. Low back pain among nurses: a follow-up beginning at entry to the nursing school. Spine (Phila Pa 1976) 2005;30:2334-41.	Outcome criteria not fulfilled.
176	Wiktorin C, Vingard E, Mortimer M, Pernold G, Wigaeus-Hjelm E, Kilbom A, et al. Interview versus questionnaire for assessing physical loads in the population-based MUSIC-Norrtalje Study. Am J Ind Med 1999;35:441-55.	Outcome criteria not fulfilled.
177	Williams RA, Pruitt SD, Doctor JN, Epping-Jordan JE, Wahlgren DR, Grant I, et al. The contribution of job satisfaction to the transition from acute to chronic low back pain. Arch Phys Med Rehabil 1998;79:366-74.	Outcome criteria not fulfilled.
178	Vingard E, Alfredsson L, Hagberg M, Kilbom A, Theorell T, Waldenstrom M, et al. To what extent do current and past physical and psychosocial occupational factors explain care-seeking for low back pain in a working population? Results from the Musculo-skeletal Intervention Center-Norrtalje Study. Spine (Phila Pa 1976) 2000;25:493-500.	Outcome criteria not fulfilled.
179	Yang LQ, Spector PE, Chang CH, Gallant-Roman M, Powell J. Psychosocial precursors and physical consequences of workplace violence towards nurses: a longitudinal examination with naturally occurring groups in hospital settings. Int J Nurs Stud 2012;49:1091-102.	Outcome criteria not fulfilled.

180	Yassi A, Khokhar J, Tate R, Cooper J, Snow C, Vallentyne S. The epidemiology of back injuries in nurses at a large Canadian tertiary care hospital: implications for prevention. Occup Med (Lond). 1995;45(4):215-20.	Outcome criteria not fulfilled.
181	Zhao I, Bogossian F, Turner C. The effects of shift work and interaction between shift work and overweight/obesity on low back pain in nurses: results from a longitudinal study. J Occup Environ Med 2012;54:820-5.	Outcome criteria not fulfilled.
182	Zochling J, Bohl-Buhler MH, Baraliakos X, Feldtkeller E, Braun J. Infection and work stress are potential triggers of ankylosing spondylitis. Clin Rheumatol 2006;25:660-6.	Outcome criteria not fulfilled.
183	Zwerling C, Ryan J, Schootman M. A case-control study of risk factors for industrial low back injury. The utility of preplacement screening in defining high-risk groups. Spine (Phila Pa 1976). 1993;18(9):1242-7.	Outcome criteria not fulfilled.

Table S11. Excluded articles based on full-text read and reason for exclusion.

Reference: studies published after January 10, 2014		Reason for exclusion
1	M. Abid, H. U. Khan, M. H. Abid, A. Ijaz, M. Ahmad, M. T. Naeem, (2021). "Association of occupational risk factors with the level of lumbar disc nucleus pulposus herniation." Pakistan Journal of Medical and Health Sciences **15**(10): 2863-2864.	Study design criteria not fulfilled.
2	Ahlholm, V. H., Ronkko, V., Ala-Mursula, L., Karppinen, J., & Oura, P. (2021). Modeling the Multidimensional Predictors of Multisite Musculoskeletal Pain Across Adulthood-A Generalized Estimating Equations Approach. *Front Public Health*, *9*, 709778.	Outcome criteria not fulfilled.
3	Alghadir, A., Zafar, H., & Iqbal, Z. A. (2015). Work-related musculoskeletal disorders among dental professionals in Saudi Arabia. *J Phys Ther Sci*, *27*(4), 1107-1112.	Study design criteria not fulfilled.
4	Alghadir, A., Zafar, H., Iqbal, Z. A., & Al-Eisa, E. (2017). Work-Related Low Back Pain Among Physical Therapists in Riyadh, Saudi Arabia. *Workplace Health Saf*, *65*(8), 337-345.	Study design criteria not fulfilled.
5	Alrowayeh, H. N., Alnaser, M. Z., Alshatti, T. A., Saeed, R. S. (2021). Prevalence and Risk Factors of Work-Related Lower Back Pain among Radiographers in the State of Kuwait. Radiol Res Pract 2021 Vol. 2021 Pages 5365260.	Study design criteria not fulfilled.
6	Alziyadi, R. H., Elgezery, M. H., & Alziyadi, R. H. (2021). Prevalence of Low Back Pain and Its Associated Risk Factors among Female Nurses Working in a tertiary hospital in Dhahran, Eastern Province, Saudi Arabia. *World Family Medicine*, *19*(1), 173-182.	Study design criteria not fulfilled.
7	Amin, R., Safdar, B., & Masood, M. H. (2019). PSYCHOSOCIAL DETERMINANTS OF BACKACHE IN FEMALES. *Indo American Journal of Pharmaceutical Sciences*, *6*(6), 12915-12921.	Outcome criteria not fulfilled.
8	Andersen, L. L., Vinstrup, J., Sundstrup, E., Skovlund, S. V., Villadsen, E., & Thorsen, S. V. (2021). Combined ergonomic exposures and development of musculoskeletal pain in the general working population: A prospective cohort study. *Scand J Work Environ Health*, *47*(4), 287-295.	Outcome criteria not fulfilled.
9	Andersen, L. L., Vinstrup, J., Villadsen, E., Jay, K., & Jakobsen, M. D. (2019). Physical and Psychosocial Work Environmental Risk Factors for Back Injury among Healthcare Workers: Prospective Cohort Study. *Int J Environ Res Public Health*, *16*(22).	Outcome criteria not fulfilled.
10	Arcury, T. A., Chen, H., Mora, D. C., Walker, F. O., Cartwright, M. S., & Quandt, S. A. (2016). The effects of work organization on the health of immigrant manual workers: A longitudinal analysis. *Arch Environ Occup Health*, *71*(2), 66-73.	Outcome criteria not fulfilled.
11	Arvidsson, I., Gremark Simonsen, J., Lindegard-Andersson, A., Bjork, J., & Nordander, C. (2020). The impact of occupational and personal factors on musculoskeletal pain - a cohort study of female nurses, sonographers and teachers. *BMC Musculoskelet Disord*, *21*(1), 621.	Outcome criteria not fulfilled.
12	Assadi, S. (2022). "Carrying load and related health disorders and disability." Indian Journal of Occupational and Environmental Medicine **26**(2): 129-132.	Study design criteria not fulfilled.
13	Badarin, K., Hemmingsson, T., Hillert, L., & Kjellberg, K. (2021). Physical workload and increased frequency of musculoskeletal pain: a cohort study of employed men and women with baseline occasional pain. *Occup Environ Med*, *78*(8), 558-566.	Outcome criteria not fulfilled.
14	Bazazan, A., Dianat, I., Bahrampour, S., Talebian, A., Zandi, H., Sharafkhaneh, A., & Maleki-Ghahfarokhi, A. (2019). Association of musculoskeletal disorders and workload with work schedule and job satisfaction among emergency nurses. *Int Emerg Nurs*, *44*, 8-13.	Study design criteria not fulfilled.
15	Bontrup, C., Taylor, W. R., Fliesser, M., Visscher, R., Green, T., Wippert, P. M., & Zemp, R. (2019). Low back pain and its relationship with sitting behaviour among sedentary office workers. *Appl Ergon*, *81*, 102894.	Study design criteria not fulfilled.
16	Bonzini, M., Bertù, L., Conti, M., D'Amato, A., Veronesi, G., Coggon, D. N., & Ferrario, M. M. (2014). 0168 Somatising tendency, occupational strain and musculoskeletal symptoms: results from a longitudinal study among Italian nurses. *Occupational and Environmental Medicine*, *71*(Suppl 1), A21.22-A21.	Other reasons (e.g., abstract, books).

17	Bonzini, M., Bertu, L., Veronesi, G., Conti, M., Coggon, D., & Ferrario, M. M. (2015). Is musculoskeletal pain a consequence or a cause of occupational stress? A longitudinal study. *Int Arch Occup Environ Health*, *88*(5), 607-612.	Outcome criteria not fulfilled.
18	A. Boutellier, C. Nuesch, P. Suter, G. Perrot and A. Mundermann (2022). Trunk muscle function and its association with functional limitations in sedentary occupation workers with and without chronic nonspecific low back pain. Journal of Back and Musculoskeletal Rehabilitation 2022 Vol. 35 Issue 4 Pages 783-791	Outcome criteria not fulfilled.
19	Bovenzi, M., & Schust, M. (2021). A prospective cohort study of low-back outcomes and alternative measures of cumulative external and internal vibration load on the lumbar spine of professional drivers. *Scand J Work Environ Health*, *47*(4), 277-286.	Outcome criteria not fulfilled.
20	Bovenzi, M., Schust, M., Menzel, G., Hofmann, J., & Hinz, B. (2015). A cohort study of sciatic pain and measures of internal spinal load in professional drivers. *Ergonomics*, *58*(7), 1088-1102.	Outcome criteria not fulfilled.
21	Bovenzi, M., Schust, M., Menzel, G., Prodi, A., & Mauro, M. (2015). Relationships of low back outcomes to internal spinal load: a prospective cohort study of professional drivers. *Int Arch Occup Environ Health*, *88*(4), 487-499.	Outcome criteria not fulfilled.
22	Brauer, C., Mikkelsen, S., Pedersen, E. B., Moller, K. L., Simonsen, E. B., Koblauch, H., Alkjaer, T., Helweg-Larsen, K., & Thygesen, L. C. (2020). Occupational lifting predicts hospital admission due to low back pain in a cohort of airport baggage handlers. *Int Arch Occup Environ Health*, *93*(1), 111-122.	Exposure criteria not fulfilled.
23	Bugajska, J., Zolnierczyk-Zreda, D., Jedryka-Goral, A., Gasik, R., Hildt-Ciupinska, K., Malinska, M., & Bedynska, S. (2013). Psychological factors at work and musculoskeletal disorders: a one year prospective study. *Rheumatol Int*, *33*(12), 2975-2983.	Outcome criteria not fulfilled.
24	Caputo E.L., M. R. Domingues, A. D. Bertoldi, P. H. Ferreira, M. L. Ferreira, D. Shirley, et al. (2021). Are leisure-time and work-related activities associated with low back pain during pregnancy? BMC Musculoskeletal Disorders 2021 Vol. 22 Issue 1 Pages 1-8.	Outcome criteria not fulfilled.
25	Chaiklieng, S., & Suggaravetsiri, P. (2020). Low Back Pain (LBP) Incidence, Ergonomics Risk and Workers' Characteristics in Relations to LBP in Electronics Assembly Manufacturing. *Indian J Occup Environ Med*, *24*(3), 183-187.	Outcome criteria not fulfilled.
26	Chakrabarty, S., Sarkar, K., Dev, S., Das, T., Mitra, K., Sahu, S., & Gangopadhyay, S. (2016). Impact of rest breaks on musculoskeletal discomfort of Chikan embroiderers of West Bengal, India: a follow up field study. *J Occup Health*, *58*(4), 365-372.	Outcome criteria not fulfilled.
27	Chan, E. W. M., Hamid, M. S. A., Din, F. H. M., Ahmad, R., Nadzalan, A. M., & Hafiz, E. (2019). Prevalence and factors associated with low back pain among Malaysian army personnel stationed in Klang Valley. *Biomedical Human Kinetics*, *11*(1), 9-18.	Study design criteria not fulfilled.
28	Chand, R. K., Roomi, M. A., Begum, S., & Mudassar, A. (2020). Prevalence of musculoskeletal disorders, associated risk factors and coping strategies among secondary school teachers in fiji. *Rawal Medical Journal*, *45*(2), 377-381.	Study design criteria not fulfilled.
29	Chang, K. C., Lee, H. C., Yen, C. L., Liao, Y. H., Hung, J. W., & Wu, C. Y. (2021). Low back pain-associated factors in female hospital-based personal care attendants. *Work*, *69*(1), 315-322.	Study design criteria not fulfilled.
30	Chaudhary, D. K., Palei, S. K., Kumar, V., Karmakar, N. C. (2022). Whole-body vibration exposure of heavy earthmoving machinery operators in surface coal mines: a comparative assessment of transport and non-transport earthmoving equipment operators. Int J Occup Saf Ergon 2022 Vol. 28 Issue 1 Pages 174-183.	Study design criteria not fulfilled.
31	Choochouy N, Saita S, Sirithian D. Prevalence of and factors associated with occupational health problems among hill farmers in Thailand. Southeast Asian J Trop Med Public Health. 2022;53(4):368-86.	Study design criteria not fulfilled.
32	Christensen, J. O., Johansen, S., & Knardahl, S. (2017). Psychological predictors of change in the number of musculoskeletal pain sites among Norwegian employees: a prospective study. *BMC Musculoskelet Disord*, *18*(1), 140.	Outcome criteria not fulfilled.
33	Christensen, J. O., Nielsen, M. B., Finne, L. B., & Knardahl, S. (2018). Comprehensive profiles of psychological and social work factors as predictors of site-specific and multi-site pain. *Scand J Work Environ Health*, *44*(3), 291-302.	Outcome criteria not fulfilled.
34	Clausen, T., Andersen, L. L., Holtermann, A., Jorgensen, A. F., Aust, B., & Rugulies, R. (2013). Do self-reported psychosocial working conditions predict low back pain after adjustment for both physical work load and depressive symptoms? A prospective study among female eldercare workers. *Occup Environ Med*, *70*(8), 538-544.	Outcome criteria not fulfilled.
35	Clays, E., Ketels, M., & Oakman, J. (2021). Low back and neck-shoulder pain: What is the role of objective and subjective measures in determining physical and psychosocial workplace hazards in non-sedentary jobs? *International Journal of Behavioral Medicine*, *28*(SUPPL 1), S134-S135.	Study design criteria not fulfilled.
36	Coenen, P., Douwes, M., van den Heuvel, S., & Bosch, T. (2016). Towards exposure limits for working postures and musculoskeletal symptoms - a prospective cohort study. *Ergonomics*, *59*(9), 1182-1192.	Outcome criteria not fulfilled.
37	Coenen, P., Kingma, I., Boot, C. R., Bongers, P. M., & van Dieen, J. H. (2014). Cumulative mechanical low-back load at work is a determinant of low-back pain. *Occup Environ Med*, *71*(5), 332-337.	Outcome criteria not fulfilled.
38	Coenen, P., Kingma, I., Boot, C. R., Twisk, J. W., Bongers, P. M., & van Dieen, J. H. (2013). Cumulative low back load at work as a risk factor of low back pain: a prospective cohort study. *J Occup Rehabil*, *23*(1), 11-18.	Other reasons (e.g., abstract, books).
39	Coenen, P., Mathiassen, S. E., Kingma, I., Boot, C. R., Bongers, P. M., & van Dieen, J. H. (2015). The effect of the presence and characteristics of an outlying group on exposure-outcome associations. *Scand J Work Environ Health*, *41*(1), 65-74.	Outcome criteria not fulfilled.
40	Coggon, D., Ntani, G., Palmer, K. T., Felli, V. E., Harari, F., Quintana, L. A., Felknor, S. A., Rojas, M., Cattrell, A., Vargas-Prada, S., Bonzini, M., Solidaki, E., Merisalu, E., Habib, R. R., Sadeghian, F., Kadir, M. M., Warnakulasuriya, S. S. P., Matsudaira, K., Nyantumbu-Mkhize, B., Harcombe, H. (2019). Drivers of international variation in prevalence of disabling low back pain: Findings from the Cultural and Psychosocial Influences on Disability study. *Eur J Pain*, *23*(1), 35-45.	Study design criteria not fulfilled.
41	Damrongsak, M., Prapanjaroensin, A., & Brown, K. C. (2018). Predictors of Back Pain in Firefighters. *Workplace Health Saf*, *66*(2), 61-69.	Study design criteria not fulfilled.
42	Das, B., & Gangopadhyay, S. (2015). Prevalence of musculoskeletal disorders and physiological stress among adult, male potato cultivators of West Bengal, India. *Asia Pac J Public Health*, *27*(2), NP1669-1682.	Study design criteria not fulfilled.
43	Das, D., Kumar, A., & Sharma, M. (2021). Risk factors associated with musculoskeletal disorders among gemstone polishers in Jaipur, India. *Int J Occup Saf Ergon*, *27*(1), 95-105.	Study design criteria not fulfilled.

44	Das B. Ergonomic and psychosocial risk factors for low back pain among rice farmers in West Bengal, India. Work. 2022;72(3):967-77.	Study design criteria not fulfilled.
45	de Alwis, M. P., & Garme, K. (2020). Effect of occupational exposure to shock and vibration on health in high-performance marine craft occupants. *Proceedings of the Institution of Mechanical Engineers, Part M: Journal of Engineering for the Maritime Environment*, *235*(2), 394-409.	Other reasons (e.g., abstract, books).
46	de Alwis, M. P., LoMartire, R., Äng, B. O., & Garme, K. (2020). Exposure aboard high-performance marine craft increases musculoskeletal pain and lowers contemporary work capacity of the occupants. *Proceedings of the Institution of Mechanical Engineers, Part M: Journal of Engineering for the Maritime Environment*, *235*(3), 750-762.	Other reasons (e.g., abstract, books).
47	Dengler K, Hiesinger K, Tisch A. Digital transformation: The role of computer use in employee health. Econ Hum Biol. 2022;46:101137.	Outcome criteria not fulfilled.
48	Dick, R. B., Lowe, B. D., Lu, M. L., & Krieg, E. F. (2020). Trends in Work-Related Musculoskeletal Disorders From the 2002 to 2014 General Social Survey, Quality of Work Life Supplement. *J Occup Environ Med*, *62*(8), 595-610.	Study design criteria not fulfilled.
49	Dragioti, E., Gerdle, B., & Larsson, B. (2019). Longitudinal Associations between Anatomical Regions of Pain and Work Conditions: A Study from The SwePain Cohort. *Int J Environ Res Public Health*, *16*(12), 16.	Outcome criteria not fulfilled.
50	Duenas, M., Moral-Munoz, J. A., Palomo-Osuna, J., Salazar, A., De Sola, H., & Failde, I. (2020). Differences in physical and psychological health in patients with chronic low back pain: a national survey in general Spanish population. *Qual Life Res*, *29*(11), 2935-2947.	Other reasons (e.g., abstract, books).
51	Ekblom-Bak, E., Stenling, A., Salier Eriksson, J., Hemmingsson, E., Kallings, L. V., Andersson, G., Wallin, P., Ekblom, O., Ekblom, B., & Lindwall, M. (2020). Latent profile analysis patterns of exercise, sitting and fitness in adults - Associations with metabolic risk factors, perceived health, and perceived symptoms. *PLoS One*, *15*(4), e0232210.	Study design criteria not fulfilled.
52	Ekholm O, Diasso PDK, Davidsen M, Kurita GP, Sjogren P. Increasing prevalence of chronic non-cancer pain in Denmark from 2000 to 2017: A population-based survey. Eur J Pain. 2022;26(3):624-33.	Outcome criteria not fulfilled.
53	Elvis C, France N, Patience E. Risk factors for work-related musculoskeletal disorders among welders in the informal sector under resource constrained settings. Work. 2022;72(1):239-52.	Study design criteria not fulfilled.
54	Ervasti, J., Pietilainen, O., Rahkonen, O., Lahelma, E., Kouvonen, A., Lallukka, T., & Manty, M. (2019). Joint contribution of rotation of the back and repetitive movements to disability pension using job exposure matrix data. *Eur J Public Health*, *29*(6), 1079-1084.	Outcome criteria not fulfilled.
55	Essien, S. K., Bath, B., Koehncke, N., Trask, C., & Saskatchewan Farm Injury Cohort Study, T. (2016). Association Between Farm Machinery Operation and Low Back Disorder in Farmers: A Retrospective Cohort Study. *J Occup Environ Med*, *58*(6), e212-217.	Outcome criteria not fulfilled.
56	Estrada-Munoz C, Madrid-Casaca H, Salazar-Sepulveda G, Contreras-Barraza N, Iturra-Gonzalez J, Vega-Munoz A. Musculoskeletal Symptoms and Assessment of Ergonomic Risk Factors on a Coffee Farm. Appl Sci-Basel. 2022;12(15):17.	Outcome criteria not fulfilled.
57	Euro, U., Knekt, P., Rissanen, H., Aromaa, A., Karppinen, J., & Heliovaara, M. (2018). Risk factors for sciatica leading to hospitalization. *Eur Spine J*, *27*(7), 1501-1508.	Exposure criteria not fulfilled.
58	Farioli, A., Mattioli, S., Quaglieri, A., Curti, S., Violante, F. S., Coggon, D., & Andersen, B. (2014). Musculoskeletal pain in Europe: the role of personal, occupational, and social risk factors. *Scand J Work Environ Health*, *40*(1), 36-46.	Study design criteria not fulfilled.
59	Feijo FR, Pearce N, Faria NMX, Carvalho MP, Szortyka ALSC, Oliveira PAB, et al. The Role of Workplace Bullying in Low Back Pain: A Study With Civil Servants From a Middle-Income Country. J Pain. 2022;23(3):459-71.	Study design criteria not fulfilled.
60	Fliesser, M., De Witt Huberts, J., & Wippert, P. M. (2018). Education, job position, income or multidimensional indices? Associations between different socioeconomic status indicators and chronic low back pain in a German sample: a longitudinal field study. *BMJ Open*, *8*(4), e020207.	Exposure criteria not fulfilled.
61	Friel, C. P., Pascual, C. B., Duran, A. T., Goldsmith, J., & Diaz, K. M. (2020). Joint associations of occupational standing and occupational exertion with musculoskeletal symptoms in a US national sample. *Occup Environ Med*.	Study design criteria not fulfilled.
62	Gaowgzeh, R. A. M. (2019). Low back pain among nursing professionals in Jeddah, Saudi Arabia: Prevalence and risk factors. *J Back Musculoskelet Rehabil*, *32*(4), 555-560.	Study design criteria not fulfilled.
63	Garg, A., Boda, S., Hegmann, K. T., Moore, J. S., Kapellusch, J. M., Bhoyar, P., Thiese, M. S., Merryweather, A., Deckow-Schaefer, G., Bloswick, D., & Malloy, E. J. (2014). The NIOSH lifting equation and low-back pain, Part 1: Association with low-back pain in the backworks prospective cohort study. *Hum Factors*, *56*(1), 6-28.	Outcome criteria not fulfilled.
64	Garg, A., Kapellusch, J. M., Hegmann, K. T., Moore, J. S., Boda, S., Bhoyar, P., Thiese, M. S., Merryweather, A., Deckow-Schaefer, G., Bloswick, D., & Malloy, E. J. (2014). The NIOSH lifting equation and low-back pain, Part 2: Association with seeking care in the backworks prospective cohort study. *Hum Factors*, *56*(1), 44-57.	Outcome criteria not fulfilled.
65	Glambek, M., Nielsen, M. B., Gjerstad, J., Einarsen, S., & Croft. (2018). Gender differences in the relationship between workplace bullying and subjective back and neck pain: A two-wave study in a Norwegian probability sample. *J Psychosom Res*, *106*, 73-75.	Outcome criteria not fulfilled.
66	Gold JE, Punnett L, Gore RJ, ProCare Research T. Predictors of low back pain in nursing home workers after implementation of a safe resident handling programme. Occup Environ Med 2017; 74(6): 389-95.	Exposure criteria not fulfilled.
67	Gunal, A., & Demirturk, F. (2021). Occupational hazards, sleep quality and musculoskeletal problems of pregnant workers. *J Obstet Gynaecol*, 1-5.	Study design criteria not fulfilled.
68	Gustafsson, K., Marklund, S., Aronsson, G., & Leineweber, C. (2019). Physical work environment factors affecting risk for disability pension due to mental or musculoskeletal diagnoses among nursing professionals, care assistants and other occupations: a prospective, population-based cohort study. *BMJ Open*, *9*(10), e026491.	Outcome criteria not fulfilled.
69	Habib, M. M. (2015). Ergonomic risk factor identification for sewing machine operators through supervised occupational therapy fieldwork in Bangladesh: A case study. *Work*, *50*(3), 357-362.	Study design criteria not fulfilled.
70	Halonen, J. I., Shiri, R., Manty, M., Sumanen, H., Solovieva, S., Viikari-Juntura, E., Kahonen, M., Lehtimaki, T., Raitakari, O. T., & Lallukka, T. (2019). Exposure to heavy physical work from early to later adulthood and primary healthcare visits due to musculoskeletal diseases in midlife: a register linked study. *BMJ Open*, *9*(8), e031564.	Outcome criteria not fulfilled.

71	Halonen JI, Virtanen M, Leineweber C, Rod NH, Westerlund H, Magnusson Hanson LL. Associations between onset of effort-reward imbalance at work and onset of musculoskeletal pain: analyzing observational longitudinal data as pseudo-trials. Pain 2018; 159(8): 1477-83.	Exposure criteria not fulfilled.
72	Hansen, B. B., Kirkeskov, L., Begtrup, L. M., Boesen, M., Bliddal, H., Christensen, R., Andreasen, D. L., Kristensen, L. E., Flachs, E. M., & Kryger, A. I. (2019). Early occupational intervention for people with low back pain in physically demanding jobs: A randomized clinical trial. *PLoS Med*, *16*(8), e1002898.	Outcome criteria not fulfilled.
73	Hassani M, Hesampour R, Bartnicka J, Monjezi N, Ezbarami SM. Evaluation of working conditions, work postures, musculoskeletal disorders and low back pain among sugar production workers. Work. 2022.	Outcome criteria not fulfilled.
74	Hemati, K., Darbandi, Z., Kabir-Mokamelkhah, E., Poursadeghiyan, M., Ghasemi, M. S., Mohseni-Ezhiye, M., Abdolahian, Y., Aghilinejad, M., Ali Salehi, M., & Dehghan, N. (2020). Ergonomic intervention to reduce musculoskeletal disorders among flour factory workers. *Work*, *67*(3), 611-618.	Other reasons (e.g., abstract, books).
75	Henriques, M., Sacadura-Leite, E. M., & Serranheira, F. (2019). Low back pain among hospital nursing assistants. *Rev Bras Med Trab*, *17*(3), 370-377.	Study design criteria not fulfilled.
76	Hong, C., Lee, C. G., & Song, H. (2021). Characteristics of lumbar disc degeneration and risk factors for collapsed lumbar disc in Korean farmers and fishers. *Annals of Occupational and Environmental Medicine*, *33*(1), e16.	Outcome criteria not fulfilled.
77	Hung, Y. J., Shih, T. T., Chen, B. B., Hwang, Y. H., Ma, L. P., Huang, W. C., Liou, S. H., Ho, I. K., & Guo, Y. L. (2014). The dose-response relationship between cumulative lifting load and lumbar disk degeneration based on magnetic resonance imaging findings. *Phys Ther*, *94*(11), 1582-1593.	Study design criteria not fulfilled.
78	Hviid Andersen, J., Frost, P., Frølund Thomsen, J., Donbaek Jensen, L., & Wulff Svendsen, S. (2014). Back Surgery in relation to occupational lifting. A cohort study based on the Musculoskeletal Research Database at the Danish Ramazzini Centre. *Occupational and Environmental Medicine*, *71*(Suppl 1), A33.31-A33.	Other reasons (e.g., abstract, books).
79	Ishimoto, Y., C. Cooper, G. Ntani, H. Yamada, H. Hashizume, K. Nagata, et al. (2019). Is radiographic lumbar spondylolisthesis associated with occupational exposures? Findings from a nested case control study within the Wakayama spine study. BMC Musculoskelet Disord 2019 Vol. 20 Issue 1 Pages 618.	Outcome criteria not fulfilled.
80	Ishimoto, Y., C. Cooper, G. Ntani, H. Yamada, H. Hashizume, K. Nagata, et al. (2019). Factory and construction work is associated with an increased risk of severe lumbar spinal stenosis on MRI: A case control analysis within the wakayama spine study. Am J Ind Med 2019 Vol. 62 Issue 5 Pages 430-438.	Outcome criteria not fulfilled.
81	Jackson JA, Liv P, Sayed-Noor AS, Punnett L, Wahlstrom J. Risk factors for surgically treated cervical spondylosis in male construction workers: a 20-year prospective study. Spine Journal. 2022.	Outcome criteria not fulfilled.
82	Jo, H., Lim, O. B., Ahn, Y. S., Chang, S. J., & Koh, S. B. (2021). Negative Impacts of Prolonged Standing at Work on Musculoskeletal Symptoms and Physical Fatigue: The Fifth Korean Working Conditions Survey. *Yonsei Med J*, *62*(6), 510-519.	Study design criteria not fulfilled.
83	Jorgensen, M. B., Nabe-Nielsen, K., Clausen, T., & Holtermann, A. (2013). Independent effect of physical workload and childhood socioeconomic status on low back pain among health care workers in Denmark. *Spine (Phila Pa 1976)*, *38*(6), E359-366.	Outcome criteria not fulfilled.
84	Kapellusch, J. M., Garg, A., Boda, S., Hegmann, K. T., Moore, J. S., Thiese, M. S., Merryweather, A., Tomich, S., Foster, J. C., Bloswick, D., & Malloy, E. J. (2014). Association between lifting and use of medication for low back pain: results from the Backworks Prospective Cohort Study. *J Occup Environ Med*, *56*(8), 867-877.	Outcome criteria not fulfilled.
85	Kawaguchi, M., Matsudaira, K., Sawada, T., Koga, T., Ishizuka, A., Isomura, T., & Coggon, D. (2017). Assessment of potential risk factors for new onset disabling low back pain in Japanese workers: findings from the CUPID (cultural and psychosocial influences on disability) study. *BMC Musculoskelet Disord*, *18*(1), 334.	Outcome criteria not fulfilled.
86	Khansa, I., Khansa, L., Westvik, T. S., Ahmad, J., Lista, F., & Janis, J. E. (2018). Work-Related Musculoskeletal Injuries in Plastic Surgeons in the United States, Canada, and Norway. *Plast Reconstr Surg*, *141*(1), 165e-175e.	Study design criteria not fulfilled.
87	Kim HR. Associations Between Workplace Violence, Mental Health, and Physical Health among Korean Workers: The Fifth Korean Working Conditions Survey. Workplace Health & Safety. 2022;70(3):161-72.	Study design criteria not fulfilled.
88	Kim, Y. H., & Jung, M. H. (2016). Effect of occupational health nursing practice on musculoskeletal pains among hospital nursing staff in South Korea. *Int J Occup Saf Ergon*, *22*(2), 199-206.	Study design criteria not fulfilled.
89	Korshoj, M., Jorgensen, M. B., Hallman, D. M., Lagersted-Olsen, J., Holtermann, A., & Gupta, N. (2018). Prolonged sitting at work is associated with a favorable time course of low-back pain among blue-collar workers: a prospective study in the DPhacto cohort. *Scand J Work Environ Health*, *44*(5), 530-538.	Outcome criteria not fulfilled.
90	Kumar V, Palei SK, Karmakar NC, Chaudhary DK. Whole-Body Vibration Exposure vis-a-vis Musculoskeletal Health Risk of Dumper Operators Compared to a Control Group in Coal Mines. Saf Health Work. 2022;13(1):73-7.	Outcome criteria not fulfilled.
91	Lagersted-Olsen, J., Thomsen, B. L., Holtermann, A., Sogaard, K., & Jorgensen, M. B. (2016). Does objectively measured daily duration of forward bending predict development and aggravation of low-back pain? A prospective study. *Scand J Work Environ Health*, *42*(6), 528-537.	Outcome criteria not fulfilled.
92	Lallukka, T., Viikari-Juntura, E., Viikari, J., Kahonen, M., Lehtimaki, T., Raitakari, O. T., & Solovieva, S. (2017). Early work-related physical exposures and low back pain in midlife: the Cardiovascular Risk in Young Finns Study. *Occup Environ Med*, *74*(3), 163-168.	Outcome criteria not fulfilled.
93	Larson RE, Johnson AW, Bruening D, Ridge ST, Mitchell UH. Low back pain and lumbar multifidus cross-sectional area, multifidus activation, and low back force in healthcare workers. Int J Ind Ergon. 2022;88:5.	Study design criteria not fulfilled.
94	Ling CF, Radin Umar RZ, Ahmad N. Development of a predictive model for work-relatedness of MSDs among semiconductor back-end workers. Int J Occup Saf Ergon. 2022;28(2):872-82.	Outcome criteria not fulfilled.
95	Liu, H.-C., Cheng, Y., & Ho, J.-J. (2020). Associations of ergonomic and psychosocial work hazards with musculoskeletal disorders of specific body parts: A study of general employees in Taiwan. *International Journal of Industrial Ergonomics*, *76*, 8.	Study design criteria not fulfilled.
96	Liu, L., Chen, S. G., Tang, S. C., Wang, S., He, L. H., Guo, Z. H., Li, J. Y., Yu, S. F., & Wang, Z. X. (2015). How Work Organization Affects the Prevalence of WMSDs: A Case-control Study. *Biomed Environ Sci*, *28*(9), 627-633.	Study design criteria not fulfilled.
97	Lovgren, M., Gustavsson, P., Melin, B., & Rudman, A. (2014). Neck/shoulder and back pain in new graduate nurses: A growth mixture modeling analysis. *Int J Nurs Stud*, *51*(4), 625-639.	Outcome criteria not fulfilled.
98	Lu, M. L., Waters, T. R., Krieg, E., & Werren, D. (2014). Efficacy of the revised NIOSH lifting equation to predict risk of low-back pain associated with manual lifting: a one-year prospective study. *Hum Factors*, *56*(1), 73-85.	Outcome criteria not fulfilled.

99	Lundberg, U. (2015). Work conditions and back pain problems. *Stress Health*, *31*(1), 1-4.	Other reasons (e.g., abstract, books).
100	Lunde, L. K., Koch, M., Knardahl, S., & Veiersted, K. B. (2017). Associations of objectively measured sitting and standing with low-back pain intensity: a 6-month follow-up of construction and healthcare workers. *Scand J Work Environ Health*, *43*(3), 269-278.	Outcome criteria not fulfilled.
101	Lunde, L. K., Koch, M., Merkus, S. L., Knardahl, S., Waersted, M., & Veiersted, K. B. (2019). Associations of objectively measured forward bending at work with low-back pain intensity: a 2-year follow-up of construction and healthcare workers. *Occup Environ Med*, *76*(9), 660-667.	Outcome criteria not fulfilled.
102	Luo, Y., Wang, J., Pei, J., Rong, Y., Liu, W., Tang, P., Cai, W., & Yin, G. (2020). Interactions between the MMP-3 gene rs591058 polymorphism and occupational risk factors contribute to the increased risk for lumbar disk herniation: A case-control study. *J Clin Lab Anal*, *34*(7), e23273.	Exposure criteria not fulfilled.
103	Magnusson Hanson, L. L., Madsen, I. E., Rugulies, R., Peristera, P., Westerlund, H., & Descatha, A. (2017). Temporal relationships between job strain and low-back pain. *Scand J Work Environ Health*, *43*(5), 396-404.	Other reasons (e.g., abstract, books).
104	Matsudaira, K., Kawaguchi, M., Isomura, T., Arisaka, M., Fujii, T., Takeshita, K., Kitagawa, T., Miyoshi, K., & Konishi, H. (2013). Identification of risk factors for new-onset sciatica in Japanese workers: findings from the Japan epidemiological research of Occupation-related Back pain study. *Spine (Phila Pa 1976)*, *38*(26), E1691-1700.	Other reasons (e.g., abstract, books).
105	Melloh M, Elfering A, Stanton TR, et al. Who is likely to develop persistent low back pain? A longitudinal analysis of prognostic occupational factors. Work 2013; 46(3): 297-311.	Exposure criteria not fulfilled.
106	Mierswa, T., & Kellmann, M. (2017). Psychological detachment as moderator between psychosocial work conditions and low back pain development. *Int J Occup Med Environ Health*, *30*(2), 313-327.	Outcome criteria not fulfilled.
107	Mierswa, T., Kellmann, M., & Alexopoulos. (2017). Differences in low back pain occurrence over a 6-month period between four recovery-stress groups. *Work*, *58*(2), 193-202.	Study design criteria not fulfilled.
108	Minghelli, B., Ettro, N., Simao, J., & Mauricio, K. (2019). Work-related self-reported musculoskeletal disorders in hypermarket cashiers: a study in south of Portugal. *Med Lav*, *110*(3), 191-201.	Study design criteria not fulfilled.
109	Mishra, S., & Sarkar, K. (2021). Work-related musculoskeletal disorders and associated risk factors among urban metropolitan hairdressers in India. *J Occup Health*, *63*(1), e12200.	Study design criteria not fulfilled.
110	Miwa, S., Yokogawa, A., Kobayashi, T., Nishimura, T., Igarashi, K., Inatani, H., & Tsuchiya, H. (2015). Risk factors of recurrent lumbar disk herniation: a single center study and review of the literature. *J Spinal Disord Tech*, *28*(5), E265-269.	Outcome criteria not fulfilled.
111	Monaco, M. G. L., Uccello, R., Muoio, M., Greco, A., Spada, S., Coggiola, M., Pedata, P., Caputo, F., Chiodini, P., Miraglia, N., & Aublet-Cuvelier. (2019). Work-related upper limb disorders and risk assessment among automobile manufacturing workers: A retrospective cohort analysis. *Work*, *64*(4), 755-761.	Outcome criteria not fulfilled.
112	Monnier, A., Djupsjobacka, M., Larsson, H., Norman, K., & Ang, B. O. (2016). Risk factors for back pain in marines; a prospective cohort study. *BMC Musculoskelet Disord*, *17*, 319.	Outcome criteria not fulfilled.
113	Monnier, A., Larsson, H., Nero, H., Djupsjobacka, M., & Ang, B. O. (2019). A longitudinal observational study of back pain incidence, risk factors and occupational physical activity in Swedish marine trainees. *BMJ Open*, *9*(5), e025150.	Exposure criteria not fulfilled.
114	Monteiro, L. F., dos Santos, J. W., de Alsina, O. L. S., Univ Fed Sergipe, S. C. S. B., Univ Tiradentes, I. T., & Res, A. S. B. (2020). Association Between Manual Handling of Loads and Occupational Low Back Pain: A Case-Control Study with Brazilians Workers. *16th International Symposium on Occupational Safety and Hygiene (SHO)*, *277*, 471-479.	Other reasons (e.g., abstract, books).
115	Moshe, S., Zack, O., Finestone, A. S., Mishal, M., Segal, N., Slodownik, D., & Yagev, Y. (2016). The incidence and worsening of newly diagnosed low back pain in a population of young male military recruits. *BMC Musculoskelet Disord*, *17*, 279.	Study design criteria not fulfilled.
116	Mozafari, A., Vahedian, M., Mohebi, S., & Najafi, M. (2015). Work-related musculoskeletal disorders in truck drivers and official workers. *Acta Med Iran*, *53*(7), 432-438.	Study design criteria not fulfilled.
117	Muslim, K., & Nussbaum, M. A. (2015). Musculoskeletal symptoms associated with posterior load carriage: An assessment of manual material handling workers in Indonesia. *Work*, *51*(2), 205-213.	Study design criteria not fulfilled.
118	Muthukrishnan, R., & Maqbool Ahmad, J. (2020). Ergonomic risk factors and risk exposure level of nursing tasks: association with work-related musculoskeletal disorders in nurses. *European Journal of Physiotherapy*, *23*(4), 248-253.	Study design criteria not fulfilled.
119	Nambiema, A., Bertrais, S., Bodin, J., Fouquet, N., Aublet-Cuvelier, A., Evanoff, B., Descatha, A., & Roquelaure, Y. (2020). Proportion of upper extremity musculoskeletal disorders attribuTable Sto personal and occupational factors: results from the French Pays de la Loire study. *BMC Public Health*, *20*(1), 456.	Outcome criteria not fulfilled.
120	Ncube, F., Kanda, A., & Dhlakama, P. (2019). Postural risk associated with Wooden Steel Chairs and Stackable Arm Chairs in a low-income country. *Work*, *64*(3), 579-586.	Study design criteria not fulfilled.
121	Neupane, S., Nygard, C. H., Prakash, K. C., von Bonsdorff, M. B., von Bonsdorff, M. E., Seitsamo, J., Rantanen, T., Ilmarinen, J., & Leino-Arjas, P. (2018). Multisite musculoskeletal pain trajectories from midlife to old age: a 28-year follow-up of municipal employees. *Occup Environ Med*, *75*(12), 863-870.	Outcome criteria not fulfilled.
122	Ng, Y. M., Voo, P., & Maakip, I. (2019). Psychosocial factors, depression, and musculoskeletal disorders among teachers. *BMC Public Health*, *19*(1), 234.	Study design criteria not fulfilled.
123	Ngabirano, L., Fadel, M., Leclerc, A., Evanoff, B. A., Dale, A. M., Roquelaure, Y., & Descatha, A. (2020). Comparison Between a Job-Exposure Matrix (JEM) Score and Self-Reported Exposures for Carrying Heavy Loads Over the Working Lifetime in the CONSTANCES Cohort. *Ann Work Expo Health*, *64*(4), 455-460.	Study design criteria not fulfilled.
124	Nissen, L. R., Marott, J. L., Gyntelberg, F., & Guldager, B. (2014). Deployment-related risk factors of low back pain: a study among danish soldiers deployed to Iraq. *Mil Med*, *179*(4), 451-458.	Study design criteria not fulfilled.
125	Nordin, M., Bolin, M., & Allvin. (2014). Do Sex Differences in the Association between Work Exposure and Health in the Manufacturing Industry Depend on Work Context? Results from the WOLF-Study. *Psychology*, *05*(08), 896-907.	Outcome criteria not fulfilled.
126	Oakman, J., Neupane, S., & Nygard, C. H. (2016). Does age matter in predicting musculoskeletal disorder risk? An analysis of workplace predictors over 4 years. *Int Arch Occup Environ Health*, *89*(7), 1127-1136.	Outcome criteria not fulfilled.

127	Oakman J, Ketels M, Clays E. Low back and neck pain: objective and subjective measures of workplace psychosocial and physical hazards. Int Arch Occup Environ Health. 2021;94(7):1637-44.	Outcome criteria not fulfilled.
128	Oliveira, V. C., Ferreira, M. L., Refshauge, K. M., Maher, C. G., Griffin, A. R., Hopper, J. L., & Ferreira, P. H. (2015). Risk factors for low back pain: insights from a novel case-control twin study. *Spine J*, *15*(1), 50-57.	Outcome criteria not fulfilled.
129	Palfreyman, S. (2015). The effect of daily walking steps on preventing neck and low back pain in sedentary workers: A 1-year prospective cohort study. *International Journal of Osteopathic Medicine*, *18*(2), 155-156.	Outcome criteria not fulfilled.
130	Pandalai, S. P., Wheeler, M. W., & Lu, M. L. (2017). Non-chemical Risk Assessment for Lifting and Low Back Pain Based on Bayesian Threshold Models. *Saf Health Work*, *8*(2), 206-211.	Outcome criteria not fulfilled.
131	Park, J., Han, B. Y., & Kim, Y. (2017). Gender differences in occupations and complaints of musculoskeletal symptoms: Representative sample of South Korean workers. *Am J Ind Med*, *60*(4), 342-349.	Study design criteria not fulfilled.
132	Park, J., & Kim, Y. (2020). Factors Related to Physical and Mental Health in Workers With Different Categories of Employment. *J Occup Environ Med*, *62*(7), 511-518.	Study design criteria not fulfilled.
133	Piranveyseh, P., Motamedzade, M., Osatuke, K., Mohammadfam, I., Moghimbeigi, A., Soltanzadeh, A., & Mohammadi, H. (2016). Association between psychosocial, organizational and personal factors and prevalence of musculoskeletal disorders in office workers. *Int J Occup Saf Ergon*, *22*(2), 267-273.	Study design criteria not fulfilled.
134	Plouvier, S., Chastang, J. F., Cyr, D., Bonenfant, S., Descatha, A., Goldberg, M., & Leclerc, A. (2015). Occupational biomechanical exposure predicts low back pain in older age among men in the Gazel Cohort. *Int Arch Occup Environ Health*, *88*(4), 501-510.	Outcome criteria not fulfilled.
135	Prakash, K. C., Neupane, S., Leino-Arjas, P., von Bonsdorff, M. B., Rantanen, T., von Bonsdorff, M. E., Seitsamo, J., Ilmarinen, J., & Nygard, C. H. (2017). Work-Related Biomechanical Exposure and Job Strain as Separate and Joint Predictors of Musculoskeletal Diseases: A 28-Year Prospective Follow-up Study. *Am J Epidemiol*, *186*(11), 1256-1267.	Outcome criteria not fulfilled.
136	Puschmann, A. K., Driesslein, D., Beck, H., Arampatzis, A., Moreno Catala, M., Schiltenwolf, M., Mayer, F., & Wippert, P. M. (2020). Stress and Self-Efficacy as Long-Term Predictors for Chronic Low Back Pain: A Prospective Longitudinal Study. *J Pain Res*, *13*, 613-621.	Outcome criteria not fulfilled.
137	Ramond-Roquin, A., Bodin, J., Serazin, C., Parot-Schinkel, E., Ha, C., Richard, I., Petit Le Manach, A., Fouquet, N., & Roquelaure, Y. (2015). Biomechanical constraints remain major risk factors for low back pain. Results from a prospective cohort study in French male employees. *Spine J*, *15*(4), 559-569.	Outcome criteria not fulfilled.
138	Rasmussen, C. D. N., Holtermann, A., & Jorgensen, M. B. (2018). Recall Bias in Low Back Pain Among Workers: Effects of Recall Period and Individual and Work-Related Factors. *Spine (Phila Pa 1976)*, *43*(12), E727-E733.	No measure of an association.
139	Rasmussen-Barr, E., Grooten, W. J. A., Hallqvist, J., Holm, L. W., & Skillgate, E. (2017). Are job strain and sleep disturbances prognostic factors for low-back pain? A cohort study of a general population of working age in Sweden. *J Rehabil Med*, *49*(7), 591-597.	Outcome criteria not fulfilled.
140	Reme, S. E., Shaw, W. S., Boden, L. I., Tveito, T. H., O'Day, E. T., Dennerlein, J. T., & Sorensen, G. (2014). Worker assessments of organizational practices and psychosocial work environment are associated with musculoskeletal injuries in hospital patient care workers. *Am J Ind Med*, *57*(7), 810-818.	Study design criteria not fulfilled.
141	Ricco, M., Pezzetti, F., & Signorelli, C. (2017). Back and neck pain disability and upper limb symptoms of home healthcare workers: A case-control study from Northern Italy. *Int J Occup Med Environ Health*, *30*(2), 291-304.	Outcome criteria not fulfilled.
142	Ropponen, A., Narusyte, J., Silventoinen, K., & Svedberg, P. (2020). Health behaviours and psychosocial working conditions as predictors of disability pension due to different diagnoses: a population-based study. *BMC Public Health*, *20*(1), 1507.	Outcome criteria not fulfilled.
143	Ropponen, A., Samuelsson, A., Alexanderson, K., & Svedberg, P. (2013). Register-based data of psychosocial working conditions and occupational groups as predictors of disability pension due to musculoskeletal diagnoses: a prospective cohort study of 24,543 Swedish twins. *BMC Musculoskelet Disord*, *14*, 268.	Outcome criteria not fulfilled.
144	Roy, T. C., Lopez, H. P., & Adams. (2013). A comparison of deployed occupational tasks performed by different types of military battalions and resulting low back pain. *Mil Med*, *178*(8), e937-943.	Outcome criteria not fulfilled.
145	Roy, T. C., Lopez, H. P., & Piva, S. R. (2013). Loads worn by soldiers predict episodes of low back pain during deployment to Afghanistan. *Spine (Phila Pa 1976)*, *38*(15), 1310-1317.	Outcome criteria not fulfilled.
146	Roy, T. C., Piva, S. R., Christiansen, B. C., Lesher, J. D., Doyle, P. M., Waring, R. M., Irrgang, J. J., Moore, C. G., Brininger, T. L., & Sharp, M. A. (2016). Heavy Loads and Lifting are Risk Factors for Musculoskeletal Injuries in Deployed Female Soldiers. *Mil Med*, *181*(11), e1476-e1483.	Outcome criteria not fulfilled.
147	Rufa'i, A. A., Sa'idu, I. A., Ahmad, R. Y., Elmi, O. S., Aliyu, S. U., Jajere, A. M., & Digil, A. A. (2015). Prevalence and Risk Factors for Low Back Pain Among Professional Drivers in Kano, Nigeria. *Arch Environ Occup Health*, *70*(5), 251-255.	Study design criteria not fulfilled.
148	Runeson-Broberg, R., Lindgren, T., & Norback, D. (2014). Musculoskeletal symptoms and psychosocial work environment, among Swedish commercial pilots. *Int Arch Occup Environ Health*, *87*(7), 685-693.	Study design criteria not fulfilled.
149	Sadeghi-Yarandi M, Ghasemi M, Ghanjal A, Sepandi M, Soltanzadeh A. The Prediction of Chronicity in Patients With Acute and Subacute Nonspecific Low Back Pain and Associated Risk Factors: A Case-Control Study. Pain Manag Nurs. 2022.	No measure of an association.
150	Sadeghian, F., Coggon, D., Ntani, G., & Hosseinzadeh, S. (2015). Predictors of low back pain in a longitudinal study of Iranian nurses and office workers. *Work*, *51*(2), 239-244.	Outcome criteria not fulfilled.
151	Sain, M. K., & Meena, M. (2019). Identifying musculoskeletal issues and associated risk factors among clay brick kiln workers. *Ind Health*, *57*(3), 381-391.	Study design criteria not fulfilled.
152	Sain, M. K., & Meena, M. L. (2018). Exploring the musculoskeletal problems and associated risk-factors among brick kiln workers. *International Journal of Workplace Health Management*, *11*(6), 395-410.	Study design criteria not fulfilled.
153	Salve, U. R. (2015). Prevalence of musculoskeletal discomfort among the workers engaged in jewelry manufacturing. *Indian J Occup Environ Med*, *19*(1), 44-55.	Other reasons (e.g., abstract, books).
154	Salo S, Hurri H, Rikkonen T, Sund R, Kroger H, Sirola J. Association between severe lumbar disc degeneration and self-reported occupational physical loading. J Occup Health. 2022;64(1):14.	Study design criteria not fulfilled.
155	Samaei, S. E., Mostafaee, M., Jafarpoor, H., & Hosseinabadi, M. B. (2017). Effects of patient-handling and individual factors on the prevalence of low back pain among nursing personnel. *Work*, *56*(4), 551-561.	Study design criteria not fulfilled.

156	Sauter M, Barthelme J, Müller C, Liebers F. Manual handling of heavy loads and low back pain among different occupational groups: results of the 2018 BIBB/BAuA employment survey. BMC Musculoskeletal Disorders. 2021;22(1):1-14.	Study design criteria not fulfilled.
157	Schlussel, A. T., & Maykel, J. A. (2019). Ergonomics and Musculoskeletal Health of the Surgeon. *Clin Colon Rectal Surg*, *32*(6), 424-434.	Other reasons (e.g., abstract, books).
158	Serranheira, F., Sousa-Uva, M., Heranz, F., Kovacs, F., & Sousa-Uva, A. (2020). Low Back Pain (LBP), work and absenteeism. *Work*, *65*(2), 463-469.	Study design criteria not fulfilled.
159	Shankar, S., Naveen Kumar, R., Mohankumar, P., & Jayaraman, S. (2017). Prevalence of work-related musculoskeletal injuries among South Indian hand screen-printing workers. *Work*, *58*(2), 163-172.	Study design criteria not fulfilled.
160	Shankar, S., Shanmugam, M., & Srinivasan, J. (2015). Workplace factors and prevalence of low back pain among male commercial kitchen workers. *J Back Musculoskelet Rehabil*, *28*(3), 481-488.	Study design criteria not fulfilled.
161	Shiri, R., Falah-Hassani, K., Heliovaara, M., Solovieva, S., Amiri, S., Lallukka, T., Burdorf, A., Husgafvel-Pursiainen, K., & Viikari-Juntura, E. (2019). Risk Factors for Low Back Pain: A Population-Based Longitudinal Study. *Arthritis Care Res (Hoboken)*, *71*(2), 290-299.	Outcome criteria not fulfilled.
162	Sihawong R, Sitthipornvorakul E, Paksaichol A, Janwantanakul P. Predictors for chronic neck and low back pain in office workers: a 1-year prospective cohort study. J Occup Health 2016; 58(1): 16-24.	Exposure criteria not fulfilled.
163	Silva, C., Barros, C., Cunha, L., Carnide, F., & Santos, M. (2016). Prevalence of back pain problems in relation to occupational group. *International Journal of Industrial Ergonomics*, *52*, 52-58.	Study design criteria not fulfilled.
164	Skillgate E, Isacson Hjortzberg M, Strömwall P, Hallqvist J, Onell C, Holm LW, et al. Non-Preferred Work and the Incidence of Spinal Pain and Psychological Distress-A Prospective Cohort Study. Int J Environ Res Public Health. 2021;18(19).	Outcome criteria not fulfilled.
165	Solecki, L. (2014). Complaints of low back pain among private farmers exposed to whole body vibration. *Med Pr*, *65*(1), 55-64.	Other reasons (e.g., abstract, books).
166	Solovev, A., Watanabe, Y., Kitamura, K., Takahashi, A., Kobayashi, R., Saito, T., Takachi, R., Kabasawa, K., Oshiki, R., Platonova, K., Tsugane, S., Iki, M., Sasaki, A., Yamazaki, O., Watanabe, K., & Nakamura, K. (2020). Total physical activity and risk of chronic low back and knee pain in middle-aged and elderly Japanese people: The Murakami cohort study. *Eur J Pain*, *24*(4), 863-872.	Exposure criteria not fulfilled.
167	Sterud, T., Johannessen, H. A., & Tynes, T. (2016). Do Work-Related Mechanical and Psychosocial Factors Contribute to the Social Gradient in Low Back Pain?: A 3-Year Follow-Up Study of the General Working Population in Norway. *Spine (Phila Pa 1976)*, *41*(13), 1089-1095.	Outcome criteria not fulfilled.
168	Sterud, T., & Tynes, T. (2013). Work-related psychosocial and mechanical risk factors for low back pain: a 3-year follow-up study of the general working population in Norway. *Occup Environ Med*, *70*(5), 296-302.	Outcome criteria not fulfilled.
169	Stevens, M. L., Boyle, E., Hartvigsen, J., Mansell, G., Sogaard, K., Jorgensen, M. B., Holtermann, A., & Rasmussen, C. D. N. (2019). Mechanisms for reducing low back pain: a mediation analysis of a multifaceted intervention in workers in elderly care. *Int Arch Occup Environ Health*, *92*(1), 49-58.	Outcome criteria not fulfilled.
170	Sundstrup, E., & Andersen, L. L. (2017). Hard Physical Work Intensifies the Occupational Consequence of Physician-Diagnosed Back Disorder: Prospective Cohort Study with Register Follow-Up among 10,000 Workers. *Int J Rheumatol*, *2017*, 1037051.	Outcome criteria not fulfilled.
171	Tang, R., Kapellusch, J. M., Hegmann, K. T., Thiese, M. S., Wang, I., & Merryweather, A. S. (2020). Evaluating Different Measures of Low Back Pain Among U.S. Manual Materials Handling Workers: Comparisons of Demographic, Psychosocial, and Job Physical Exposure. *Hum Factors*, 18720820971101.	Outcome criteria not fulfilled.
172	Telaprolu, N., & Anne, S. D. (2014). Physical and psychological work demands as potential risk factors for musculoskeletal disorders among workers in weaving operations. *Indian JOccup Environ Med*, *18*(3), 129-134.	Study design criteria not fulfilled.
173	Thiede, M., Liebers, F., Seidler, A., Gravemeyer, S., & Latza, U. (2014). Gender specific analysis of occupational diseases of the low back caused by carrying, lifting or extreme trunk flexion--use of a prevention index to identify occupations with high prevention needs. *Am J Ind Med*, *57*(2), 233-244.	Study design criteria not fulfilled.
174	Thiese, M. S., Lu, M. L., Merryweather, A., Tang, R., Ferguson, S. A., Malloy, E. J., Marras, W. S., Hegmann, K. T., & Kapellusch, J. (2020). Psychosocial Factors and Low Back Pain Outcomes in a Pooled Analysis of Low Back Pain Studies. *J Occup Environ Med*, *62*(10), 810-815.	Study design criteria not fulfilled.
175	Udom, C., Kanlayanaphotporn, R., & Janwantanakul, P. (2019). Predictors for Nonspecific Low Back Pain in Rubber Farmers: A 1-Year Prospective Cohort Study. *Asia Pac J Public Health*, *31*(1), 7-17.	Outcome criteria not fulfilled.
176	Upadhyay R, Bhattacherjee A, Patra AK, Chau N. Association between Whole-Body Vibration exposure and musculoskeletal disorders among dumper operators: A case-control study in Indian iron ore mines. Work. 2022;71(1):235-47.	Outcome criteria not fulfilled.
177	Urquhart, D. M., Kelsall, H. L., Hoe, V. C., Cicuttini, F. M., Forbes, A. B., Sim, M. R., & Burton. (2013). Are psychosocial factors associated with low back pain and work absence for low back pain in an occupational cohort? *Clin J Pain*, *29*(12), 1015-1020.	Study design criteria not fulfilled.
178	Vargas-Prada, S., Serra, C., Martinez, J. M., Ntani, G., Delclos, G. L., Palmer, K. T., Coggon, D., & Benavides, F. G. (2013). Psychological and culturally-influenced risk factors for the incidence and persistence of low back pain and associated disability in Spanish workers: findings from the CUPID study. *Occup Environ Med*, *70*(1), 57-62.	Outcome criteria not fulfilled.
179	Vinstrup, J., Jakobsen, M. D., & Andersen, L. L. (2020). Perceived Stress and Low-Back Pain Among Healthcare Workers: A Multi-Center Prospective Cohort Study. *Front Public Health*, *8*, 297.	Outcome criteria not fulfilled.
180	Vinstrup, J., Jakobsen, M. D., Madeleine, P., & Andersen, L. L. (2020). Physical exposure during patient transfer and risk of back injury & low-back pain: prospective cohort study. *BMC Musculoskelet Disord*, *21*(1), 715.	Outcome criteria not fulfilled.
181	Wang F, Chen K, Lin QS, Ma YG, Huang H, Wang CAF, et al. Earlier or heavier spinal loading is more likely to lead to recurrent lumbar disc herniation after percutaneous endoscopic lumbar discectomy. J Orthop Surg Res. 2022;17(1):7.	Outcome criteria not fulfilled.
182	Wippert PM, Valencia LP, Driesslein D. Stress and Pain. Predictive (Neuro)Pattern Identification for Chronic Back Pain: A Longitudinal Observational Study. Front Med. 2022;9:14.	Outcome criteria not fulfilled.
183	Wixted, F., & O'Sullivan, L. (2019). Task engagement as a mediator between the cognitive demands of sustained attention and musculoskeletal complaints: A structural equation modelling approach. *Work*, *64*(3), 623-634.	Study design criteria not fulfilled.

184	Wurzelbacher, S. J., Lampl, M. P., Bertke, S. J., & Tseng, C. Y. (2020). The effectiveness of ergonomic interventions in material handling operations. *Appl Ergon*, *87*, 103139.	Outcome criteria not fulfilled.
185	Xiao, H., McCurdy, S. A., Stoecklin-Marois, M. T., Li, C. S., & Schenker, M. B. (2013). Agricultural work and chronic musculoskeletal pain among Latino farm workers: the MICASA study. *Am J Ind Med*, *56*(2), 216-225.	Study design criteria not fulfilled.
186	Yang, S. T., Park, M. H., & Jeong, B. Y. (2020). Types of manual materials handling (MMH) and occupational incidents and musculoskeletal disorders (MSDs) in motor vehicle parts manufacturing (MVPM) industry. *International Journal of Industrial Ergonomics*, *77*, 9.	Study design criteria not fulfilled.
187	Yazgan E, Ozkan NF, Ulutas BH. A questionnaire-based musculoskeletal disorder assessment for aircraft maintenance technicians. Aircr Eng Aerosp Technol. 2022;94(2):240-7.	Outcome criteria not fulfilled.
188	Yovi, E. Y., & Prajawati, W. (2015). High Risk Posture on Motor-Manual Short Wood Logging System in Acacia mangium Plantation. *Jurnal Manajemen Hutan Tropika (Journal of Tropical Forest Management)*, *21*(1), 11-18.	Study design criteria not fulfilled.
189	Yovi, E. Y., & Yamada, Y. (2019). Addressing Occupational Ergonomics Issues in Indonesian Forestry. *Croatian Journal of Forest Engineering*, *40*(2), 351-363.	Study design criteria not fulfilled.
190	Yue, P., Xu, G., Li, L., & Wang, S. (2014). Prevalence of musculoskeletal symptoms in relation to psychosocial factors. *Occup Med (Lond)*, *64*(3), 211-216.	Study design criteria not fulfilled.
191	Zamri, E. N., Hoe, V. C. W., & Moy, F. M. (2020). Predictors of low back pain among secondary school teachers in Malaysia: a longitudinal study. *Ind Health*, *58*(3), 254-264.	Outcome criteria not fulfilled.
192	Zarra, T., & Lambrianidis, T. (2014). Musculoskeletal disorders amongst Greek endodontists: a national questionnaire survey. *Int Endod J*, *47*(8), 791-801.	Study design criteria not fulfilled.
193	Zhang, D., & Huang, H. (2017). Prevalence of work-related musculoskeletal disorders among sonographers in China: results from a national web-based survey. *J Occup Health*, *59*(6), 529-541.	Study design criteria not fulfilled.
194	Zhang, M. Y., Bai, Z. Z., Zhao, X. F., Ieee, Ieee, & Dalian Univ Technol, F. I. E. D. C. M. D. P. R. C. (2017). Real-time Risk Assessment for Construction Workers' Trunk Posture Using Mobile Sensor. *International Conference on Robotics and Automation Sciences (ICRAS)*, 153-157.	Study design criteria not fulfilled.

Appendix F. Summary of characteristics.

Table S12. Summary of characteristics of the 26 included articles.

Author	Design	Population	Outcome		Exposure	
			Definition	**Assessment**	**Definition**	**Assessment**
Aghilinejad 2015	Cohort	The cohort consisted of male workers in one of the biggest metal-industry factories in Iran followed from 2012 to 2013. In total, 218 workers received a questionnaire and 33 were excluded. Therefore, 185 workers were eligible for the analysis (49 chronic participants and 136 cured for acute LBP) with a mean age of 35.96 (SD=7.33).	LBP: Self-reported chronic pain ≥3 months. Participants are followed up until 3 months after onset during monthly phone calls. If the pain had ended before the 3 months, they were categorised in the acute group.	Interview.	*Mechanical exposures*: Physical effort, sustained sitting, whole-body vibration, non-neutral postures, lifting (5-15 kg.), lifting (>15 kg.), and hands above shoulder.	Questionnaire.
Ahsan 2013	Case-control	240 cases with LDH were recruited by their physicians from a spinal surgery unit in Dhaka, Bangladesh (2007-2010). 200 cases (124 males and 76 females) were eligible and 200 controls were matched on age, sex, and area of residence from a non-spinal related orthopaedic department. The overall mean age was 39.42 years (SD=NS).	LDH: With low back pain with or without sciatica collected from radiological and physicians' examinations.<br>Diagnostic criteria:<br>- Dominant leg pain than back pain.<br>- Restricted Straight Leg Raise.<br>- Neurological deficit.<br>- Positive MRI findings.	Physical examination, radiological examination, and MRI.	*Mechanical exposures*: Sitting/standing, bending/twisting, lifting/carrying heavy loads, vibration, and physical effort at work.	Interview.
Alhalabi 2015	Case-control	513 cases (134 males and 379 females) were recruited from an outpatient neurology clinic in Damascus, Syria (2011-2012). 398 controls (135 males and 263 females) were selected from family members and friends. Age ranged from 18 to more than 70 years (SD=NS).	LBP: Chronic low back pain lasting ≥3 months.	Interview.	*Mechanical exposures*: Lifting heavy objects and awkward positions (i.e., bending, standing, and sitting)	Interview.
Bergmann 2017	Case-control	German population-based sample consisting of 915 cases (431 males and 484 females) treated in a hospital or special orthopaedic and neurosurgical practices. 422 controls (220 males and 202 females) were randomly drawn from a 1 % sample of residents aged between 25 to 70 years who stated no LBP in the previous 12 months.	LDH*:* Diagnosed by CT or MRI with radiculopathy related to the herniated segment. Morphological criteria of herniation were used from AJNR "nomenclature ad classification of lumbar disc pathology". Consensus criteria was developed to quantify criteria on the basis of disc displacement metrics.<br><br>LDN*:* Measured in the sagittal plan using MRT/CT images and x-rays from the lateral native. If the lumbar spine was not met orthogonally, the centre of the vertebral body endplates was determined, and the central disc space was measured from this point.	MRI or CT.	*Mechanical exposures*:<br>- Manual material handling of loads: lifting, carrying, pulling, pushing, throwing, shovelling loads weighing at least 5 kg.<br>- Intensive-load working postures: forward trunk inclination, lateral trunk bending, trunk torsion, over-head<br>working, kneeling, squatting, heel-sitting.<br>- Forces: assembly work and lever activities, manual patient handling;<br>- Whole-body vibration (WBV, horizontal and vertical<br>direction considered).	Interview.
Esquirol 2017	Cohort	3,237 male and female employed and retired workers from south of France were followed with a 5-year follow-up period with age ranging from 32 to 52 years. For the analysis, 804 males and 756 females were eligible and divided in:	LBP: Participants were considered as suffering from chronic LBP if they reported LBP or a specific treatment for such pain for at least 6 months at both measurements' points.	Questionnaire.	*Mechanical exposures*: carrying heavy loads and non-neutral postures.	Questionnaire.

		- 231 participants reported "persistent chronic LBP" (they were compared with a non-persistent chronic LBP group containing 199 participants)<br>- 255 participants reported "incidence of chronic LBP" (they were compared with a non-chronic LBP group containing 875 participants).	Participants are divided in three outcome groups:<br>1. Answering "no" to CLBP at both measurements.<br>2. Incidence CLBP – answering "no" at baseline but "yes" at follow-up.<br>3. Persistence CLBP – answering "yes" at both measurements.			
Euro 2019	Cohort	The original study population was selected using a two-stage cluster sample from Finland comprising 8,000 males and females aged 30 to 59 years. A total of 7,217 participated in the screening phase and after exclusions, this cohort comprised 1,900 males and 1,991 females.	Sciatica: Hospitalisations for sciatica were obtained from "Care register for Health Care" covering all Finnish hospitals (public and private) with diagnoses based on ICD-8-10. Sciatica was defined by the codes:<br>- ICD-8 = 353.99, 725.10 or 725.19.<br>- ICD-9 = 7225A, 7227C or 7228C.<br>- ICD-10 = G55.1, M51.1, M51.2, M54.3 or M54.4.	Register information.	*Mechanical exposures*: Physical strenuousness work, lifting, non-neutral postures, prolonged standing, sitting, whole-body vibration, constant movements and paced work.	Questionnaire.
Halonen 2019	Cohort	Participants were selected from the Swedish Longitudinal Occupational Survey of Health Study. Those responding to any two subsequent surveys in 2010 to 2016 were included. Of 17,962 participants, 12,222 participants (55% females) were free of LBP and 5,740 participants (61 % females) had LBP at baseline.<br>Mean age at baseline was 54.1 years (SD=11.3).	LBP: Self-reported pain in the last 3 months defined as either "pain that affects my life a little" or "pain that affects my life a lot".<br><br>LBP was dichotomised into:<br>- No affecting pain (no pain or pain that does not affect life).<br>- Affecting pain (pain affecting life a little or a lot).<br><br>For the main analysis, participants were divided into:<br>- Incident (free from LBP at baseline).<br>- Recurrent (LBP at baseline).	Questionnaire.	*Mechanical exposures*: Twisting and lifting.	Questionnaire.
Herin 2014	Cohort	Representative sample of subjects randomly selected from 7 French regions using exhaustive lists under the supervision of 400 volunteering occupational physicians. A total of 21,378 participants were included at baseline, 18,695 responded at follow-up, and 12,591 was eligible both at baseline and follow-up. For the analyses, 1206 participants (787 males and 419 females) were presented with LBP who came from 4 years of birth (1938, 1943, 1948 and 1953).	LBP: Self-reported musculoskeletal pain in combination with physicians' examination. Chronic musculoskeletal pain was defined as subjects who, on the day of examination, declared low back pain for at least 6 months who also presented with positive clinical signs.	Interview and physical examination.	*Mechanical exposures*: Forceful effort, effort with tools, heavy loads, movements, postures, and vibration.<br>*Psychosocial exposures*: Psychological demands and decision latitude.	Questionnaire.
Heuch 2017	Cohort	The whole population of Nord-Trøndelag, Norway, above the age of 20 years was invited to participate. The study was restricted to the age of 30-69 years.	LBP: Chronic low back pain was defined as LBP persisting for at least 3 months continuously during the past year (yes/no).	Questionnaire.	*Mechanical exposures*: Were measured in four categories asking the participants to indicate the baseline level of physical activity at work:	Questionnaire.

		Follow-up included 24,280 participants, and in the analysis, 14,915 (7,335 males and 7,580 females) were eligible.	Information on LBP were collected from one question: “During the last year, have you suffered from pain and/or stiffness in your muscles and joints that has lasted for at least 3 consecutive months?”<br><br>If yes, a follow-up question was given: “Where did you have these complaints?” Which included pre-specified body regions.		1. Substantially sedentary work.<br>2. work involving walking, but no heavy lifting.<br>3. Work involving both walking and heavy lifting.<br>4. Particularly strenuous physical work.	
Jansen 2004	Cohort	The cohort consisted of workers from 7 Dutch nursing homes and homes for elderly with various professions such as nurses, care givers, kitchen workers, transportation etc.<br>1208 subjects were invited to participate in 1998-1999 and 769 agreed to participate. After 1 year, 523 were observed again with a mean age of 40.7 years of age (SD=9.7).<br>Information on sex was not provided.	LBP: Low back pain with disability was defined by Von Korff’s disability score >50 points which indicated “high disability”.	Questionnaire.	*Mechanical exposures*: Trunk flexion between 20 to 45º, trunk flexion >45º, and lifting and carrying loads >10 kg.	Observations
Jørgensen 2013	Cohort	The cohort consisted of Danish participants employed in 14 private and public companies. 5,249 men took part in the baseline examination. 3,833 were without LBP at baseline and was then included for the analyses aged between 40 and 59 years.	LDH: Hospitalisation due to herniated lumbar disc disease was identified in the National Hospital Register between 1977 and 2003 using the ICD-8 code 725.11 and ICD-10 code M51.1.	Register information.	*Mechanical exposures*: strenuous work and ergonomic load to the back.	Questionnaire
Krause 2004	Cohort	The cohort consisted of 1974 transit vehicle operators from a railway in California, USA. After exclusion, the eligible study population comprised 1,841 workers. Of these, 1,503 participants responded to an additional questionnaire with the final sample being 1,233 (1055 males and 178 females) study participants for the analyses with a mean age of 46.7 years (SD=7.8).	Low back injury: First incidence of a compensated non-traumatic low back injury to the lumbar or sacral region of the spine. LBI was divided into "more severe" (post-laminectomy syndrome, spinal stenosis, herniated lumbar disc, sciatica, or spinal instability) and less severe (degenerative changes of the lumbar spine or non-specific low back pain).<br><br>Outcome was measured by linking the participant’s social security number to their worker’s compensation file including all claims. These claims were then linked to the medical bill review file obtaining the ICD-9 code. Only cases with a “definite” diagnostic ICD-9 code on any physician bill record during the course of the claim were included.	Register information.	*Mechanical exposures:* Driving.	Questionnaire
Latza 2002	Cohort	The cohort consisted of 571 male construction workers (age 17-59 years) from Hamburg, Germany. After 3 years, all workers were approached for a follow-up survey and 488 were	Chronic LBP: $\geq$90 days of low back pain during the last 12 months.	Interview.	*Mechanical exposures:* Work tasks including laying 6 different kinds of bricks or stones during shifts in the preceding 12 months and stone load.	Interview

		willing to participate with a mean age of 33.1 years (SD=10.0).				
Matsudaira(42)(42)(42)(42)2014	Cohort	Employees were recruited from 16 local offices (e.g., office workers, nurses, salesmen and manufacturing engineers) in/near Tokyo, Japan. Baseline questionnaire was distributed to 6,140 participants and 5,310 responded. After 1 year, 3,811 participants completed the follow-up questionnaire.<br>Among the 3,811 participants, 1,675 reported mild LBP during the past year at baseline with a mean age of 43.1 years (SD=10.1) and 78.6% males. Of these, 43 participants reported persistent LBP within the 1-year follow-up period.	Persistent LBP: LBP interfering with work (grade 2 or 3) with disability lasting longer than 3 months during a 1-year follow-up period.<br><br>Grades:<br>- Grade: No LBP.<br>- Grade 1: LBP that does not interfere with work.<br>- Grade 2: LBP that interferes with work but no<br>absence from work.<br>- Grade 3: LBP that interferes with work, leading to sick-leave.	Questionnaire.	*Mechanical exposures*: Manual handling at work, twisting, hours of desk work, and physical workload.	Questionnaire
Matsudaira 2015	Cohort	Employees were recruited from 16 local offices (e.g., office workers, nurses, salesmen and manufacturing engineers) in/near Tokyo, Japan. Baseline questionnaire was distributed to 6140 participants and 5,310 responded. After 1 year, 3,811 participants completed the follow-up questionnaire.<br>Among the 3,811 employees, 171 reported LBP and experiencing work interferences with or without sick-leave during a month prior to baseline with a mean age of 42.9 years (SD=10.1) (71.4% males). Of these, 29 developed chronic disabling LBP during a year prior to the follow-up period.	Chronic disabling LBP: LBP that interfered with work for >3 months, regardless of sick leave (grade 2 and 3) during a 1-year follow-up period.<br><br>Grades:<br>- Grade: No LBP.<br>- Grade 1: LBP that does not interfere with work.<br>- Grade 2: LBP that interferes with work but no<br>absence from work.<br>- Grade 3: LBP that interferes with work, leading to sick-leave.	Questionnaire.	*Mechanical exposures*: Manual handling at work, twisting, hours of desk work, and physical workload.	Questionnaire
Matsudaira 2019	Cohort	Participants were recruited from different occupational groups in/near Tokyo. Occupational groups varied from nurses, office workers, sales/marketing to transportation. Baseline questionnaire was distributed to 3,187 employees and 2651 returned the questionnaire. Of these, 1809 participants returned the completed follow-up questionnaire.<br>Among the 1809 participant, only 198 participants with disabling LBP during the month before baseline were included. The mean age was 36.0 (SD=9.1) where 69% were males. Of these 198, 35 had chronic disabling LBP during the 1-year follow-up period.	Chronic disabling LBP: LBP that interfered with work for >3 months regardless of sick leave (grade 2 and 3) during the 1-year follow-up period.<br><br>Grades:<br>- Grade 0: No LBP.<br>- Grade 1: LBP that does not interfere with work.<br>- Grade 2: LBP that interferes with work but no absence from work.<br>- Grade 3: LBP that interferes with work, leading to sick-leave.	Questionnaire.	*Mechanical exposures*: Use a keyboard, move wrist/finger, bend elbow, hands above shoulder height, lift weights of 25 kg. by hand, kneel/squat 1 hour, stand, twist back/stoop for 4 hours, and drive for 4 hours.	Questionnaire
Picavet 2016	Cohort	An age and sex stratified random sample of 12,405 inhabitants of Doetinchem, The Netherlands (20-60 years of age) was invited at baseline (1987-1992).	LBP: Information on LBP was measured using a single question: "have you had any trouble, discomfort or pain in the lower back during	Questionnaire.	*Mechanical exposures*: STable Soccupational sitting. STable Ssitters were defined as workers who had indicated to have a sedentary job in at least 3 out 4	Questionnaire

		At the first out of five follow-ups (1993-2012), a random sample of 7,769 was invited.<br>In this study, data from round 2 to 5 was used. Only working participants were selected resulting in 3,597 participants at baseline. Finally, 1,694 were eligible for all survey-rounds. For the analyses, 1,509 participants were included with a mean age of 54 years (SD=6) for sTable Ssitters and 53 years (SD=6) for sTable Snon-sitters.	the last 12 months?" (yes/no) and chronic low back pain was measured by a subsequent question on duration defined as pain $\geq$3 months.		measurements, whereas sTable Snon-sitters were defined as those having indicated in at least 3 out of 4 measurements to not have a sedentary job.	
Prado-Leon 2014	Case-control	77 cases (57% males) were enrolled from a Family Medicine Units comprising industrial workers from a diverse range of manufacturing plants in Mexico. Cases were selected from a list of disability rulings and between the age of 18 and 55 years.<br>154 controls (68.8% males) were identified at the same Family Medicine Units as cases from records or files for insured workers.<br>They were randomly selected and within 2-years of age of the cases – but not outside the age of 18 to 55.	Spondylarthrosis: was confirmed through reviewing cases files, and the following parameters were used to confirm the diagnosis:<br>- Clinical exam.<br>- Imaging scan (radiography, computerized axial tomography, and magnetic resonance imaging).<br>- Diagnostic review by a committee of the Mexican Social Security Institute experts.	Physical examination and imaging scan.	*Mechanical exposures*: Pushing/pulling, weight of load when pushing/pulling, hours spent pushing/pulling, job frequency, and daily job frequency.	Questionnaire
Seidler 2003	Case-control	437 male patients recruited at neurological clinics in Germany aged between 25 and 65 years. After exclusion, 225 cases were eligible and divided into:<br>- LDH with osteochondrosis/spondylosis = 131, mean age 43.7<br>- "Pure" LDH = 94, mean age of 40.0.<br><br>107 controls (males) were from a random population group (mean age of 43) and 90 patients (males) admitted to hospital for urolithiasis who had no radiographically confirmed osteochondrosis or spondylosis (mean age of 40).	LBP: Herniation of the lumbar discs or osteochondrosis/spondylosis of the lumbar spine associated with chronic complaints (low back pain, sciatica). The diagnosis of lumbar disc herniation had to have been confirmed by computed tomography or magnetic resonance imaging.<br><br>The radiographs were reassessed by reference radiologist.	MRI and CT.	*Mechanical exposures:* Manual handling, forward bending, whole-body vibration, and sedentary work.	Interview
Seidler 2009	Case-control	915 patients with lumbar disc herniation or lumbar disc narrowing were recruited at hospitals or orthopaedic practices in Germany aged between 25 and 70 years. 901 controls were randomly selected from a 1 % random sample of residents aged between 25 and 70 years, drawn by the local population registration offices.<br>Females with LDH = 278, mean age of 47.1 (SD=11.8).<br>Females with LDN = 206, mean age of 56.0 (SD=9.8).<br>Controls = 448, mean age of 46.4 (SD=11.8).<br>Males with LDH = 286, mean age of 48.7 (SD=11.1).<br>Males with LDN = 145, mean age of 55.0 (SD=10.7).	LDH: Confirmed by a reference radiologist by either computerised tomography or by magnetic resonance imaging.<br><br>LDN: Primarily based on X-ray.<br><br>To qualify as cases, MRI, CT and X-rays of the lumbar spine were re-assessed by one reference radiologist separately for each disc and vertebral body. Furthermore, the clinical diagnosis had to be verified by one experienced reference orthopaedist	MRI, CT, and X-rays.	*Mechanical exposures*<br>- Manual material handling of loads (e.g., lifting, carrying,<br>pulling, pushing, throwing, shovelling loads weighing at least 5 kg)<br>- Trunk inclination and twisting and whole-body vibration.	Interview

		Controls = 453, mean age of 47.3 (SD=12.6).				
Seidler 2011	Case-control	915 patients with lumbar disc herniation or lumbar disc narrowing were recruited at hospitals or orthopaedic practices in Germany aged between 25 and 70 years. 901 controls were randomly selected from a 1 % random sample of residents aged between 25 and 70 years, drawn by the local population registration offices.<br>Females with LDH = 278, mean age of 47.1 (SD=11.8).<br>Females with LDN = 206, mean age of 56.0 (SD=9.8).<br>Controls = 448, mean age of 46.4 (SD=11.8).<br>Males with LDH = 286, mean age of 48.7 (SD=11.1).<br>Males with LDN = 145, mean age of 55.0 (SD=10.7).<br>Controls = 453, mean age of 47.3 (SD=12.6).	LDH: Confirmed by a reference radiologist by either computerised tomography or by magnetic resonance imaging.<br><br>LDN: Primarily based on X-ray.<br><br>To qualify as cases, MRI, CT and X-rays of the lumbar spine were re-assessed by one reference radiologist separately for each disc and vertebral body. Furthermore, the clinical diagnosis had to be verified by one experienced reference orthopaedist	MRI, CT, and X-rays.	*Mechanical exposures*<br>- Manual material handling of loads (e.g., lifting, carrying,<br>pulling, pushing, throwing, shovelling loads weighing at least 5 kg)<br>- Trunk inclination and twisting and whole-body vibration.	Interview
Seyedmehdi 2016	Cohort	All industrial workers from a large Iranian rubber factory with acute non-specific LBP in the past 2 weeks were included (2011-2912). Diagnosis of acute non-specific LBP at baseline was made by two occupational medicine specialists. The cohort consisted of 542 participants and 511 completed the 1-year follow-up (500 males and 11 females) with a mean age of 37.6 years (SD=5.8).	LBP was assessed 3, 6, 9 months and at 1 year after baseline asking the question: "Have you recovered from your LBP". If the answer was "yes"<br>The question would follow: "How long did it last?"<br><br>Participants with LBP were divided in 2 groups:<br>1. LBP lasting <3 months.<br>2. LBP lasting $\geq$3 months.	Face-to-face or telephone interview.	*Mechanical exposures*: Standing position in shift work and carrying heavy loads.	Questionnaire
Sørensen 2011	Cohort	Participants were employed at 14 different private and public companies in Copenhagen, Denmark, including railway, insurance, fire brigade etc., aged between 40 to 59 years. 5249 males were invited at baseline and 3833 without LBP at baseline were entered in the study.	LDH: Hospitalisation due to herniated lumbar disc disease was identified in the National Hospital Register between 1977 and 2003 using the ICD-8 code 725.11 and ICD-10 code M51.1.	Register information.	*Mechanical exposures:* Strenuous work and ergonomic load to the back.<br><br>*Psychosocial exposures:* Mental stress at work	Questionnaire
Tubach 2004	Cohort	At baseline, the cohort included 20,624 subjects who were employees at a French electricity and gas company. Of these, a random sample of 4,018 subjects received a questionnaire about LBP. 3,240 completed the questionnaire whereas 475 were included in the analyses (405 males and 70 females) aged between 35 to 50 years.	LBP: Assessed by the question regarding suffering from sciatica the last year and if the subjects had visited a physician regarding their symptoms.	Questionnaire.	*Mechanical exposures:* Carrying loads more than 10 kg., and driving more than 2 hours a day.	Questionnaire
Vieira 2018	Case-control	119 Brazilian patients (57 males and 62 females) with chronic LBP were recruited from an outpatient hospital clinic of spinal surgery. 112 controls (23 males and 89 females) were recruited from a clinical	Disc degeneration: Was measured by MRI scans of all patients and performed by two experienced radiologists. The images were evaluated by an orthopaedic spine surgeon.	Imaging scan.	*Mechanical exposures*: Postures at work and load weight at work.	Interview-administered questionnaire

		laboratory. Median age of cases were 40 years and 32 years for controls.	The degree of disc degeneration was graded from T2-weighted images according to Pfirrmann classification, and only patients with grades 3, 4, or 5 were included in the case group.			
Wahlström 2018	Cohort	389,132 construction workers from Sweden were identified through a national register using a personal ID number assigned all inhabitants. Between 1968 and 1993, due to a nation-wide program, all workers were invited to participate in health examines ever 2-5 year.<br>In total, 288,926 men was included in the analysis and restricted to weight between 50 and 129 kg, height between 150 and 199 cm and BMI between 18.5 and 34.9.	LDH: Occurrence of hospitalisation due to lumbar disc herniation was collected from registers and defined as the ICD-9 code 722.1 or the ICD-10 code M51.1.	Register information.	*Mechanical exposures*: Whole-body vibration.	Job-exposure-matrix

Abbreviations: CT = computed tomography; ICD = International Classification of Diseases; ID = Identification; kg = kilogram; LBP = low back pain; LDH = lumbar disc herniation; LDN = lumbar disc narrowing; MRI = magnetic resonance imaging; MS = milliseconds; NS = not specified; SD = standard deviation.

Appendix G. Measure of association.

Table S13. Measure of association between occupational mechanical exposures and chronic LBP.

Author	Exposure	Outcome	Confounders	Categories of exposures	Results					
					Men		Women		Total	
					Measure of association	95% CI	Measure of association	95% CI	Measure of association	95% CI
**Lifting/carrying loads**										
Aghilinejad 2015	*Lifting*: Duration of lifting >15 kg. during a work day measured by a five-point scale and dichotomised (N=185, 49 cases and 136 controls).	Self-reported chronic pain for ≥3 months.	Age, BMI, smoking, LBP history, family LBP history, education, shift working, job type, other physical and psychosocial factors.	- Low<br>- High	1.0 OR<br>2.9 OR	-<br>1.2 – 6.9	-<br>-	-<br>-	-<br>-	-<br>-
Ahsan 2013	*Lifting*: Lifting or carrying heavy objects (daily labourer, loader, construction worker) (N=28) compared to controls (N=56).	Lumbar disc herniation.	Matched on age, sex, and area of residence.	- No<br>- Yes	-<br>-	-<br>-	-<br>-	-<br>-	1.00 OR<br>3.48 OR	-<br>1.84 – 6.59
Alhalabi 2015	*Lifting*: Heavy objects (N=346).	Chronic low back pain lasting ≥3 months.	None.	- None<br>- Non-daily<br>- Daily	-<br>-<br>-	-<br>-<br>-	-<br>-<br>-	-<br>-<br>-	1.00 OR<br>0.92 OR<br>1.44 OR	-<br>0.44 – 1.93<br>0.68 – 3.05
Bergmann 2017	*Lifting*: 10 dose models were applied comprising various thresholds for the lumbosacral compressive force, trunk inclination, or shift-related minimum threshold. Models consider loads 5 kg or more and trunk inclination from 20 to 90 degrees. The calculation includes frequency and duration of all handlings. A cumulative lifetime dose for the compressive force on the disc L5/S1 in kNh was computed (N=564 for LDH and N=531 for SDN, whereas N=422 for controls).	Lumbar disc herniation (LDH) and severe disc narrowing (SDN).	Age, unemployment, work stress, trunk inclination, and study region.	LDH:<br>- 0 to < 2.34*10^6 Nh<br>- 2.34 to < 8.98*10^6Nh<br>- ≥8.98*10^6Nh<br>SDN:<br>- 0 to < 2.34*10^6 Nh<br>- 2.34 to < 8.98*10^6Nh<br>- ≥8.98*10^6Nh<br><br>LDH:<br>- 0<br>- 0 to <1.58*10^6 Nh<br>- 1.58 to <9.06 *10^6Nh<br>- ≥9.06*10^6Nh<br>SDN:<br>- 0<br>- 0 to <1.58*10^6 Nh<br>- 1.58 to <9.06 *10^6Nh<br>- ≥9.06*10^6Nh	 1.0 OR<br>1.4 OR<br>2.2 OR<br><br>1.0 OR<br>1.7 OR<br>2.7 OR<br><br><br>-<br>-<br>-<br>-<br><br>-<br>-<br>-<br>-	 -<br>0.8 – 2.6<br>1.2 – 4.1<br><br>-<br>0.8 – 3.6<br>1.3 – 5.8<br><br><br>-<br>-<br>-<br>-<br><br>-<br>-<br>-<br>-	 -<br>-<br>-<br><br>-<br>-<br>-<br><br><br>1.0 OR<br>0.8 OR<br>1.1 OR<br>1.1 OR<br><br>1.0 OR<br>1.2 OR<br>3.0 OR<br>2.0 OR	 -<br>-<br>-<br><br>-<br>-<br>-<br><br><br>-<br>0.4 – 1.8<br>0.5 – 2.5<br>0.5 – 2.5<br><br>-<br>0.4 – 3.5<br>1.1 – 8.1<br>0.7 – 5.9	 -<br>-<br>-<br><br>-<br>-<br>-<br><br><br>-<br>-<br>-<br>-<br><br>-<br>-<br>-<br>-	 -<br>-<br>-<br><br>-<br>-<br>-<br><br><br>-<br>-<br>-<br>-<br><br>-<br>-<br>-<br>-

Esquirol<br>2017	*Lifting*: Carrying heavy loads (N=1,130 for incidence).	Self-reported chronic LBP for >6 months.<br>Incidence compared to non-chronic LBP group.	Sex, age, history of rheumatologically events., BMI, number of different jobs held, job changes, productivity-related income, and work recognition.	Incidence chronic LBP:<br>- Never<br>- Former<br>- Current	 -<br>-<br>-	 -<br>-<br>-	 -<br>-<br>-	 -<br>-<br>-	 1.00 OR<br>1.12 OR<br>1.54 OR	 -<br>0.71 – 1.77<br>1.09 – 2.18
	Lifting: Carrying heavy loads (N= 430 for persistence).	Self-reported chronic LBP for >6 months.<br>Persistence compared with participants with chronic LBP at baseline but no LBP at follow-up.	None.	Persistence chronic LBP:<br>- Never<br>- Former<br>- Current	 -<br>-<br>-	 -<br>-<br>-	 -<br>-<br>-	 -<br>-<br>-	 1.00 OR<br>0.99 OR<br>1.11 OR	 -<br>0.61 – 1.62<br>0.72 – 1.73
Euro<br>2019	*Lifting*: Lifting or carrying heavy objects – measured by typicality of working time (N=NS).	Hospitalisation of Sciatica classified by ICD-8/9/10 codes.	Age, sex, BMI, educational level, smoking, physical sedentary work, heavy work, awkward trunk postures, prolonged sitting, and whole-body vibration.	- No<br>- Yes	-<br>-	-<br>-	-<br>-	-<br>-	1.00 HR<br>2.10 HR	-<br>1.35 – 3.26
Halonen<br>2019	*Lifting*: Lifting at least 15 kg several times a day (N=12,222 for incident LBP and N=5,740 for recurrent LBP).	Self-reported LBP in the last 3 months divided into:<br>- Incident LBP (free from LBP at baseline).<br>- Recurrent LBP (LBP at baseline).	Age, sex, study survey, education, BMI, smoking, physical activity, depressive symptoms, and sleep problems.	Incident LBP:<br>- None or < 1/4 of work time<br>- 1/4 to 1/2 of work time<br>- 3/4 of work time<br>Recurrent LBP:<br>- None or < 1/4 of work time<br>- 1/4 to 1/2 of work time<br>- 3/4 of work time	 -<br>-<br>-<br><br>-<br>-<br>-	 -<br>-<br>-<br><br>-<br>-<br>-	 -<br>-<br>-<br><br>-<br>-<br>-	 -<br>-<br>-<br><br>-<br>-<br>-	 1.00 RR<br>1.31 RR<br>1.52 RR<br><br>1.00 RR<br>1.08 RR<br>1.13 RR	 -<br>1.17 – 1.46<br>1.32 – 1.74<br><br>-<br>1.03 – 1.14<br>1.07 – 1.20
Herin<br>2014	*Lifting*: exposure to carrying heavy loads (N=1,206, 787 men and 419 women).	Self-reported LBP for 6 months + clinical symptoms.	Age, sports participation, BMI, and social class.	- Low<br>- High	1.00 OR<br>1.06 OR	-<br>0.88 – 1.28	1.00 OR<br>1.02 OR	-<br>0.77 – 1.35	-<br>-	-<br>-
Jansen<br>2004	*Lifting*: Lifting and carrying loads over 10 kg. was taken as the average load for the entire occupational group. It was measured using observations on 212 workers that were randomly selected at baseline with at least 10 workers representing each	Self-reported LBP with disability.	Trunk flexion between 20 to 45º, trunk flexion >45º, decision authority, skill discretion, and work demands.	- 1 min/week<br>- 5 min/week<br>- 15 min/week<br>- 30 min/week<br>- 45 min/week	-<br>-<br>-<br>-<br>-	-<br>-<br>-<br>-<br>-	-<br>-<br>-<br>-<br>-	-<br>-<br>-<br>-<br>-	1.00 RR<br>1.05 RR<br>1.18 RR<br>1.33 RR<br>1.26 RR	-<br>0.94 – 1.17<br>0.79 – 1.77<br>0.60 – 2.95<br>0.38 – 4.20

	occupational group included in the study. Observations were made on selected workers every 20 seconds during four periods of 30 minutes each in one working day. For each occupation, the average exposure to each type of physical load was calculated as the mean percentage of time devoted to that activity (N=523).									
Latza 2002	*Manual material handling*: Duration of laying 3DF lime stones weighing about 6-10 kg. was used as proxy measure for manual material handling (N=404 without CLBP at baseline and all workers N= 488).	Self-reported chronic LBP.	Age.	Without chronic LBP at baseline:<br>- 0 h/shift<br>- >0 – <2.0 h/shift<br>- 2.0 – 8.5 h/shift<br><br>All workers:<br>- 0 h/shift<br>- >0 – <2.0 h/shift<br>- 2.0 – 8.5 h/shift	 1.00 PR<br>0.50 PR<br>2.89 PR<br><br><br>1.00 PR<br>1.13 PR<br>1.80 PR	 -<br>0.12 – 2.14<br>1.32 – 6.35<br><br><br>-<br>0.59 – 2.16<br>1.04 – 3.14	 -<br>-<br>-<br><br><br>-<br>-<br>-	 -<br>-<br>-<br><br><br>-<br>-<br>-	 -<br>-<br>-<br><br><br>-<br>-<br>-	 -<br>-<br>-<br><br><br>-<br>-<br>-
	Manual material handling: Duration of laying 2DF lime stones weighing about 4 - 6.5 kg. was used as proxy measure for manual material handling (N=404 without CLBP at baseline and all workers N= 488).	Self-reported chronic LBP.	Age.	Without chronic LBP at baseline:<br>- 0 h/shift<br>- >0 – <2.0 h/shift<br>- 2.0 – 8.5 h/shift<br><br>All workers:<br>- 0 h/shift<br>- >0 – <2.0 h/shift<br>- 2.0 – 8.5 h/shift	 1.00 PR<br>0.87 PR<br>1.98 PR<br><br><br>1.00 PR<br>0.78 PR<br>1.45 PR	 -<br>0.29 – 2.57<br>0.80 – 4.89<br><br><br>-<br>0.39 – 1.54<br>0.80 – 2.62	 -<br>-<br>-<br><br><br>-<br>-<br>-	 -<br>-<br>-<br><br><br>-<br>-<br>-	 -<br>-<br>-<br><br><br>-<br>-<br>-	 -<br>-<br>-<br><br><br>-<br>-<br>-
	Stone load: Assessed by the sum of average stone mass for each type of brick or stone multiplied by hours per day working with that stone type (N=404 without CLBP at baseline and all workers N= 488).	Self-reported chronic LBP.	Age.	Without chronic LBP at baseline:<br>- Low<br>- Medium<br>- High<br><br>All workers:<br>- Low<br>- Medium<br>- High	 1.00 PR<br>0.57 PR<br>2.10 PR<br><br><br>1.00 PR<br>0.63 PR<br>1.44 PR	 -<br>0.17 – 1.96<br>0.95 –4.65<br><br><br>-<br>0.31 – 1.30<br>0.85 – 2.46	 -<br>-<br>-<br><br><br>-<br>-<br>-	 -<br>-<br>-<br><br><br>-<br>-<br>-	 -<br>-<br>-<br><br><br>-<br>-<br>-	 -<br>-<br>-<br><br><br>-<br>-<br>-
Matsudaira 2015	*Manual materials handling*: Measured by manual handling of 20 kg objects (N=171).	Self-reported LBP interfering with work for ≥3 months.	None.	- No manual handling<br>- Manual handling of <20 kg objects<br>- Manual handling of ≥20 kg objects	-<br>-<br>-	-<br>-<br>-	-<br>-<br>-	-<br>-<br>-	1.00 OR<br>1.40 OR<br>1.84 OR	-<br>0.43 – 4.50<br>0.72 – 4.72
Matsudaira 2014	*Lifting*: Measured by frequency dichotomized by half the day (N=1,675).	Self-reported LBP interfering with work for ≥3 months.	Age, sex, obesity, smoking, and education	- Infrequent<br>- Frequent	-<br>-	-<br>-	-<br>-	-<br>-	1.00 OR<br>2.81 OR	-<br>1.18 – 6.66

	Manual materials handling: Measured by manual handling of materials at work defined as 20 kg. objects or working as a caregiver (N=1,675).	Self-reported LBP interfering with work for ≥3 months.	Age, sex, obesity, smoking, and education	- Manual handling of <20 kg. objects including desk work<br>- Manual handling of ≥20 kg. objects or working as a caregiver	-<br>-	-<br>-	-<br>-	-<br>-	1.00 OR<br>2.70 OR	-<br>1.98 – 8.67
Matsudaira 2019	*Lifting*: Lifting weights of more than 25 kg by hand (N=196).	Self-reported LBP interfering with work for ≥3 months.	None.	- No<br>- Yes	-<br>-	-<br>-	-<br>-	-<br>-	1.00 OR<br>0.89 OR	-<br>0.39 – 2.07
Seidler 2003	*Cumulated lifting/carrying*: Was measured by the squares of the weights lifted or carried at work, multiplied by the corresponding durations and summed (N=152 controls and 129 cases).	Lumbar disc herniation combined with osteochondrosis/spondylosis.	Age, region, nationality, and other disease of the lumbar spine.	- 0 kg^2 * hours<br>- >0–10 000 kg^2 * hours<br>- >10 000–150 000 kg^2 * hours<br>- >150 000 kg^2 * hours	1.0 OR<br>2.3 OR<br>5.4 OR<br>8.5 OR	-<br>0.9 – 5.6<br>2.3 – 12.6<br>3.8 – 19.1	-<br>-<br>-<br>-	-<br>-<br>-<br>-	-<br>-<br>-<br>-	-<br>-<br>-<br>-
Seidler 2009	*Manual materials handling*: Assessed by a two-step procedure. 1) A standardised computer-assisted interview identifying subjects that exceeded a certain minimum workload, 2) Comprehensive semi-standardised interview by ergonomic expert with those exceeding minimum workloads. Quantification of compressive force on the lumbosacral disc assessed with a biomechanical tool (N=453 for men with LDH, and N= 145 for men with LDN. N=448 for women with LDH and N=206 for LDN).	Lumbar disc herniation and lumbar disc narrowing.	Men, LDH: Adjusted for age, region, unemployment and Intensive-load postures.<br>Men, LDN: Adjusted for age, region, and intensive-load postures.<br>Women: Adjusted for age, region psychosocial workload and intensive-load postures.	LDH:<br>- 0-<5.0*10^6 Nh<br>- 5.0–<21.51*10^6 Nh<br>- ≥21.51*10^6 Nh<br>LDN:<br>- 0-<5.0*10^6 Nh<br>- 5.0–<21.51*10^6 Nh<br>- ≥21.51*10^6 Nh<br>LDH:<br>- 0 Nh<br>- 0 – <1.58*10^6 Nh<br>- 1.58 – <9.06*10^6 Nh<br>- >9.06*10^6 Nh<br>LDN:<br>- 0 Nh<br>- 0 – <1.58*10^6 Nh<br>- 1.58 – <9.06*10^6 Nh<br>- >9.06*10^6 Nh	 1.0 OR<br>1.2 OR<br>2.0 OR<br><br>1.0 OR<br>1.3 OR<br>2.4 OR<br><br>-<br>-<br>-<br>-<br><br>-<br>-<br>-<br>-	 -<br>0.7 – 2.0<br>1.2 – 3.5<br><br>-<br>0.7 – 2.6<br>1.2 – 4.6<br><br>-<br>-<br>-<br>-<br><br>-<br>-<br>-<br>-	 -<br>-<br>-<br><br>-<br>-<br>-<br><br>1.0 OR<br>0.8 OR<br>1.0 OR<br>0.8 OR<br><br>1.0 OR<br>1.3 OR<br>3.0 OR<br>1.9 OR	 -<br>-<br>-<br><br>-<br>-<br>-<br><br>-<br>0.4 – 1.6<br>0.5 – 1.9<br>0.4 – 1.6<br><br>-<br>0.5 – 3.3<br>1.3 – 6.8<br>0.8 – 4.4	 -<br>-<br>-<br><br>-<br>-<br>-<br><br>-<br>-<br>-<br>-<br><br>-<br>-<br>-<br>-	 -<br>-<br>-<br><br>-<br>-<br>-<br><br>-<br>-<br>-<br>-<br><br>-<br>-<br>-<br>-
Seyedmehdi 2016	*Heavy Physical load*: Was measured by the question: "Do you frequently carry heavy stuff?" (N=511).	Self-reported LBP lasting >3 months.	None.	- No<br>- Yes	-<br>-	-<br>-	-<br>-	-<br>-	1.00 OR<br>2.35 OR	-<br>1.51 – 3.64
Tubach 2004	*Carrying loads*: Was measured by carrying loads of >10 kg. (N=409).	Sciatica	Sex, driving, home repair, visit to a medical practitioner, sick leave, pain intensity, psychosomatic score, depression score, job satisfaction, long	- Never<br>- <Once a week<br>- >Once a week<br>- Everyday	-<br>-<br>-<br>-	-<br>-<br>-<br>-	-<br>-<br>-<br>-	-<br>-<br>-<br>-	1.00 OR<br>1.74 OR<br>1.22 OR<br>1.49 OR	-<br>1.02 – 2.96<br>0.69 – 2.19<br>0.79 – 2.83

			lasting LBP in 1991, sciatica in 1990.							
**Non-neutral postures**										
Aghilinejad<br>2015	*Non-neutral postures*: Duration of awkward back posture during a work day measured by five-point scale and dichotomised (N=185, 49 cases and 136 controls).	Self-reported chronic pain for >3 months.	None	- Low<br>- High	1.00 OR<br>1.35 OR	-<br>0.68 – 2.68	-<br>-	-<br>-	-<br>-	-<br>-
Ahsan<br>2013	*Bending/twisting*: Occupations involving bending and twisting postural work load isolated or combined with other factors (N=98) compared with controls (N=60).	Lumbar disc herniation.	Matched on age, sex, and area of residence.	- No<br>- Yes	-<br>-	-<br>-	-<br>-	-<br>-	1.00 OR<br>1.77 OR	-<br>0.83 – 3.78
Alhalabi<br>2015	*Awkward positions*: Bending (N=137).	Chronic low back pain lasting >3 months.	None	- None<br>- Bending	-<br>-	-<br>-	-<br>-	-<br>-	1.0 OR<br>2.3 OR	-<br>0.73 – 7.63
Bergmann<br>2017	*Trunk inclination*: 10 dose models were applied comprising various thresholds for the lumbosacral compressive force, trunk inclination, or shift-related minimum threshold. Models consider loads 5 kg or more and trunk inclination from 20 to 90 degrees. The calculation includes frequency and duration of all handlings. A cumulative lifetime dose for the compressive force on the disc L5/S1 in kNh was computed (N=564 for LDH and N= 531 for SDN, whereas N=422 for controls).	Lumbar disc herniation (LDH) and severe disc narrowing (SDN).	Age, unemployment, work stress, manual materials handling, and study region.	LDH:<br>- 0 Nh<br>- >0 to <4.85*10^6Nh<br>- 4.85 to 14.62*10^6Nh<br>- >14.62*10^6Nh<br><br>SDN:<br>- 0 Nh<br>- >0 to <4.85*10^6Nh<br>- 4.85 to 14.62*10^6Nh<br>- >14.62*10^6Nh<br><br>LDH:<br>- 0 Nh<br>- >0 to <2.77*10^6Nh<br>- 2.77 to 8.83*10^6Nh<br>- >8.83*10^6Nh<br><br>SDN:<br>- 0 Nh<br>- >0 to <2.77*10^6Nh<br>- 2.77 to 8.83*10^6Nh<br>- >8.83*10^6Nh	 1.0 OR<br>1.1 OR<br>1.7 OR<br>2.4 OR<br><br><br>1.0 OR<br>1.6 OR<br>1.6 OR<br>2.1 OR<br><br><br>-<br>-<br>-<br>-<br><br><br>-<br>-<br>-<br>-	 -<br>0.6 – 2.1<br>0.8 – 3.4<br>1.2 – 5.0<br><br><br>-<br>0.7 – 3.6<br>0.7 – 3.6<br>0.9 – 4.9<br><br><br>-<br>-<br>-<br>-<br><br><br>-<br>-<br>-<br>-	 -<br>-<br>-<br>-<br><br><br>-<br>-<br>-<br>-<br><br><br>1.0 OR<br>2.7 OR<br>2.6 OR<br>3.7 OR<br><br><br>1.0 OR<br>1.2 OR<br>1.2 OR<br>1.6 OR	 -<br>-<br>-<br>-<br><br><br>-<br>-<br>-<br>-<br><br><br>-<br>1.2 – 6.3<br>1.2 – 6.0<br>1.6 – 8.6<br><br><br>-<br>0.4 – 3.7<br>0.4 – 3.2<br>0.6 – 4.5	 -<br>-<br>-<br>-<br><br><br>-<br>-<br>-<br>-<br><br><br>-<br>-<br>-<br>-<br><br><br>-<br>-<br>-<br>-	 -<br>-<br>-<br>-<br><br><br>-<br>-<br>-<br>-<br><br><br>-<br>-<br>-<br>-<br><br><br>-<br>-<br>-<br>-
Esquirol<br>2017	*Non-neutral postures*: Classified into three modalities (N= 1,130 for incidence and N=430 for persistence).	Self-reported chronic pain for >6 months.<br>Incidence compared to non-chronic LBP	None.	Incidence chronic LBP:<br>- Never<br>- Former<br>- Current<br>Persistence chronic LBP:	 -<br>-<br>-	 -<br>-<br>-	 -<br>-<br>-	 -<br>-<br>-	 1.00 OR<br>1.20 OR<br>1.30 OR	 -<br>0.76 – 1.90<br>0.96 – 1.76

		and persistence compared to non-persistence group.		- Never<br>- Former<br>- Current	-<br>-<br>-	-<br>-<br>-	-<br>-<br>-	-<br>-<br>-	1.00 OR<br>1.41 OR<br>1.11 OR	-<br>0.81 – 2.44<br>0.73 – 1.68
Euro<br>2019	*Non-neutral postures*: Measured by typicality of awkward trunk postures at work (N=NS).	Hospitalisation of Sciatica classified by ICD-8/9/10 codes.	Age, sex, BMI, educational level, smoking, physical sedentary work, heavy work, lifting, prolonged sitting, and whole-body vibration.	- No<br>- Yes	-<br>-	-<br>-	-<br>-	-<br>-	1.00 HR<br>0.68 HR	-<br>0.43 – 1.03
Halonen<br>2019	*Twisting*: Working in twisted, bent, or otherwise unsuiTable Spositions (N= 12,222 for incident LBP and N=5,740 for recurrent LBP).	Self-reported LBP during the last 3 months divided into:<br>- Incident LBP (free from LBP at baseline).<br>- Recurrent LBP (LBP at baseline).	Age, sex, study survey, education, BMI, smoking, physical activity, depressive symptoms, and sleep problems.	Incident LBP:<br>- None or < 1/4 of working time<br>- 1/4 to 1/2 of working time<br>- 3/4 of working time<br>Recurrent LBP:<br>- None or < 1/4 of working time<br>- 1/4 to 1/2 of working time<br>- 3/4 of working time	 -<br>-<br>-<br><br>-<br>-<br>-	 -<br>-<br>-<br><br>-<br>-<br>-	 -<br>-<br>-<br><br>-<br>-<br>-	 -<br>-<br>-<br><br>-<br>-<br>-	 1.00 RR<br>1.22 RR<br>1.52 RR<br><br>1.00 RR<br>1.10 RR<br>1.19 RR	 -<br>1.11 – 1.34<br>1.37 – 1.70<br><br>-<br>1.05 – 1.16<br>1.07 – 1.20
Herin<br>2014	*Postures*: exposed to long, difficult working positions and/or non-neutral postures (N=1,206, 787 men and 419 women).	Self-reported LBP for ≥6 months.	Age, sports participation, BMI, and social class.	- Low<br>- High	1.00 OR<br>1.19 OR	-<br>1.01 – 1.39	1.00 OR<br>1.33 OR	-<br>1.07 – 1.64	-<br>-	-<br>-
Jansen<br>2004	*Postures*: Trunk flexion (20-45º) was taken as the average for the entire occupational group. Observations were made on selected workers every 20 seconds during four periods of 30 minutes each in one working day. For each occupation, the average exposure to each type of physical load was calculated as the mean percentage of time devoted to that activity (N=523).	Self-reported LBP with disability.	Trunk flexion between >45º, and lifting/carrying loads >10 kg, decision authority, skill discretion, and work demands.	- 2 h/week<br>- 3 h/week<br>- 4 h/week<br>- 5 h/week<br>- 6 h/week	-<br>-<br>-<br>-<br>-	-<br>-<br>-<br>-<br>-	-<br>-<br>-<br>-<br>-	-<br>-<br>-<br>-<br>-	1.00 RR<br>0.95 RR<br>0.90 RR<br>0.83 RR<br>0.80 RR	-<br>0.53 – 1.72<br>0.28 – 2.87<br>0.22 – 3.18<br>0.19 – 3.32
	Postures: Trunk flexion (>45º) was taken as the average for the entire occupational group. Observations were made on selected workers every 20 seconds during four periods of 30 minutes each in one working day. For each occupation, the average exposure to each type of physical load was calculated as the mean percentage of time devoted to that activity (N=523).	Self-reported LBP with disability.	Trunk flexion between 20 to 45º, trunk flexion >45º, and lifting/carrying loads >10 kg, decision authority, skill discretion, and work demands.	- 30 min./week<br>- 45 min./week<br>- 1 h/week<br>- 1 h and 30 min/week<br>- 1 h and 45 min./week	-<br>-<br>-<br>-<br>-	-<br>-<br>-<br>-<br>-	-<br>-<br>-<br>-<br>-	-<br>-<br>-<br>-<br>-	1.00 RR<br>1.31 RR<br>1.71 RR<br>2.82 RR<br>3.18 RR	-<br>1.03 – 1.65<br>1.08 – 2.72<br>1.16 – 6.86<br>1.13 – 9.00

Matsudaira 2015	*Bending:* Was measured by bending more than half of the day (N=169).	LBP interfering with work for >3 months.	None.	- Not frequent<br>- Frequent	-<br>-	-<br>-	-<br>-	-<br>-	1.00 OR<br>1.40 OR	-<br>0.58 – 3.40
	Twisting: Was measured by twisting more than half of the day (N=168).	LBP interfering with work for >3 months.	None.	- Not frequent<br>- Frequent	-<br>-	-<br>-	-<br>-	-<br>-	1.00 OR<br>1.24 OR	-<br>0.42 – 3.65
Matsudaira 2014	*Bending*: >half of the day was considered frequent (N=1,675).	LBP interfering with work for >3 months.	Age, sex, obesity, smoking, and education.	- Infrequent<br>- Frequent	-<br>-	-<br>-	-<br>-	-<br>-	1.00 OR<br>3.45 OR	-<br>1.54 – 7.72
	Twisting: >half of the day was considered frequent (N=1,675).	LBP interfering with work for >3 months.	Age, sex, obesity, smoking, and education.	- Infrequent<br>- Frequent	-<br>-	-<br>-	-<br>-	-<br>-	1.00 OR<br>4.35 OR	-<br>1.80 – 10.52
Matsudaira 2019	*Twist back/stoop*: Ergonomic work demands in an average working day (N=197).	Self-reported LBP interfering with work for >3 months<br>.	None.	- <4 hours/day<br>- >4 hours/day	-<br>-	-<br>-	-<br>-	-<br>-	1.00 OR<br>1.25 OR	-<br>0.58 – 2.69
Seidler 2003	*>90 degrees' trunk flexion forward bending*: working postures with extreme forward bending was calculated by the force on the lumbar spine at L5/S1 as Force=1,700 Newton. It was assessed with cumulated hours spent in working postures with extreme forward bending calculated up to the year of diagnosis (N=183 controls and 128 cases).	Lumbar disc herniation combined with osteochondrosis/spondylosis.	Age, region, nationality, and other disease of the lumbar spine.	- 0 hours:<br>- >0 – 1500 hours<br>- >1500 hours	1.0 OR<br>2.7 OR<br>4.5 OR	-<br>1.5 – 5.1<br>2.2 – 9.3	-<br>-<br>-	-<br>-<br>-	-<br>-<br>-	-<br>-<br>-
Seidler 2009	*Intensive-load postures*: Postures without object handling. Assessed by a two-step procedure. 1) A standardised computer-assisted interview identifying subjects that exceeded a certain minimum workload, 2) Comprehensive semi-standardised interview by ergonomic expert with those exceeding minimum workloads. Quantification of compressive force on the lumbosacral disc assessed with a biomechanical tool (N=453 for men with LDH, and N= 145 for men with LDN. N=448 for women with LDH and N=206 for LN).	Lumbar disc herniation and lumbar disc narrowing.	Men: Adjusted for age, region, and unemployment. Women: Adjusted for age, region psychosocial workload and intensive-load postures.	Men, LDH:<br>- 0 Nh<br>- >0 – <4.85*10^6Nh<br>- 4.85 – 14.62 *10^6Nh<br>- >14.62*10^6Nh<br>Men, LDN:<br>- 0 Nh<br>- >0 – <4.85*10^6Nh<br>- 4.85 – 14.62 *10^6Nh<br>- >14.62*10^6Nh<br>Women, LDH:<br>- 0 Nh<br>- >0 – <2.77*10^6Nh<br>- 2.77 – 8.83 *10^6Nh<br>- >8.83*10^6Nh<br>Women, LDN:<br>- 0 Nh<br>- >0 – <2.77*10^6Nh<br>- 2.77 – 8.83 *10^6Nh<br>- > 8.83*10^6Nh	 1.0 OR<br>1.1 OR<br>1.7 OR<br>1.9 OR<br><br>1.0 OR<br>1.3 OR<br>1.4 OR<br>1.4 OR<br><br>-<br>-<br>-<br>-<br><br>-<br>-<br>-<br>-	 -<br>0.6 – 2.0<br>0.9 – 3.2<br>1.0 – 3.5<br><br>-<br>0.6 – 2.6<br>0.7 – 2.9<br>0.7 – 2.9<br><br>-<br>-<br>-<br>-<br><br>-<br>-<br>-<br>-	 -<br>-<br>-<br>-<br><br>-<br>-<br>-<br>-<br><br>1.0 OR<br>1.9 OR<br>2.4 OR<br>3.2 OR<br><br>1.0 OR<br>0.7 OR<br>0.8 OR<br>1.1 OR	 -<br>-<br>-<br>-<br><br>-<br>-<br>-<br>-<br><br>-<br>1.0 – 3.7<br>1.2 – 4.6<br>1.6 – 6.3<br><br>-<br>0.3 – 1.7<br>0.3 – 1.9<br>0.5 – 2.7	 -<br>-<br>-<br>-<br><br>-<br>-<br>-<br>-<br><br>-<br>-<br>-<br>-<br><br>-<br>-<br>-<br>-	 -<br>-<br>-<br>-<br><br>-<br>-<br>-<br>-<br><br>-<br>-<br>-<br>-<br><br>-<br>-<br>-<br>-

**Whole-body vibrations**										
Aghilinejad<br>2015	Whole-body vibrations: Duration of whole-body vibration in a work day measured on a five-point scale and dichotomised (N=185, 49 cases and 136 controls).	Self-reported chronic pain for ≥3 months.	None.	- Low<br>- High	1.00 OR<br>1.69 OR	-<br>0.79 – 3.59	-<br>-	-<br>-	-<br>-	-<br>-
Ahsan<br>2013	*Vibrations*: Driver and machine operator involving causal exposure to vibration (N=20) compared to controls (N=46).	Lumbar disc herniation.	Matched on age, sex, and area of residence.	- No<br>- Yes	-<br>-	-<br>-	-<br>-	-<br>-	1.00 OR<br>1.58 OR	-<br>0.67 – 3.72
Bergmann<br>2017	Whole-body vibrations: 10 dose models were applied comprising various thresholds for the lumbosacral compressive force, trunk inclination, or shift-related minimum threshold. The calculation includes frequency and duration of all handlings. A cumulative lifetime dose for the compressive force on the disc L5/S1 in kNh was computed. For WBV, horizontal and vertical direction was considered. (N=564 for LDH and N= 531 for SDN, whereas N=422 for controls).	Lumbar disc herniation (LDH) and severe disc narrowing (SDN).	Age, unemployment, work stress, manual materials handling, and study region.	LDH:<br>- 0, (m/s^2)^2<br>- >0 to <364 (m/s^2)^2<br>- 364 to <1190(m/s^2)^2<br>- ≥1190(m/s^2)^2<br><br>SDN:<br>- 0, (m/s^2)^2<br>- >0 to <364 (m/s^2)^2<br>- 364 to <1190(m/s^2)^2<br>- ≥1190(m/s^2)^2	 1.0 OR<br>1.6 OR<br>0.7 OR<br>1.8 OR<br><br><br>1.0 OR<br>1.1 OR<br>1.0 OR<br>6.3 OR	 -<br>0.5 – 4.9<br>0.3 – 1.6<br>0.4 – 9.0<br><br><br>-<br>0.3 – 4.3<br>0.4 – 2.6<br>1.3 – 30.8	 -<br>-<br>-<br>-<br><br><br>-<br>-<br>-<br>-	 -<br>-<br>-<br>-<br><br><br>-<br>-<br>-<br>-	 -<br>-<br>-<br>-<br><br><br>-<br>-<br>-<br>-	 -<br>-<br>-<br>-<br><br><br>-<br>-<br>-<br>-
Euro<br>2019	*Vibrations*: Shaking of the whole-body or use of vibrating equipment – measured by typicality of working time (N=NS).	Hospitalisation of Sciatica classified by ICD-8/9/10 codes.	Age, sex, BMI, educational level, smoking, physical sedentary work, heavy work, awkward trunk postures, lifting, and prolonged sitting.	- No<br>- Yes	-<br>-	-<br>-	-<br>-	-<br>-	1.00 HR<br>1.61 HR	-<br>0.95 – 2.72
Herin<br>2014	Vibrations: Exposure to considerable vibrations and/or exposure to jolts (N=1,206, 787 males and 419 female).	Self-reported LBP for ≥6 months.	Age, sports participation, BMI, and social class.	- Low<br>- High	1.00 OR<br>1.00 OR	-<br>0.85 – 1.18	1.00 OR<br>1.73 OR	-<br>1.01 – 3.01	-<br>-	-<br>-
Seidler<br>2003	*Whole-body vibration*: Was measured by showing illustrated Table Ss of possible vehicles and classified into smooth asphalt (factor 0), damaged asphalt (factor 1), cobbled streets (2), and rough	Lumbar disc herniation combined with osteochondrosis/spondylosis.	Age, region, nationality, other disease of the lumbar spine, and sum lumbar spine force through lifting/carrying	- 0 hours<br>- >0 – 1500 hours<br>- >1500 hours<br>Weighted factor for type of terrain:<br>- 0 hours<br>- >0 – 1800 hours * weighing factor<br>- >1800 hours * weighing factor	1.0 OR<br>0.9 OR<br>1.2 OR<br><br>1.0 OR<br>1.3 OR<br>1.2 OR	-<br>0.5 – 1.9<br>0.5 – 2.7<br><br>-<br>0.6 – 2.9<br>0.5 – 2.7	-<br>-<br>-<br><br>-<br>-<br>-	-<br>-<br>-<br><br>-<br>-<br>-	-<br>-<br>-<br><br>-<br>-<br>-	-<br>-<br>-<br><br>-<br>-<br>-

	terrain (3) (N=193 controls and 129 cases).		and/or extreme forward bending.							
Wahlström<br>2018	*Whole-body vibrations*: Using a Job-exposure-matrix, whole-body vibration was graded on a 0-5 scale assessed as mean daily exposure. Referent group included foremen and white-collar workers (N=288,926).	Lumbar disc herniation.	Age, height, weight, and smoking.	- Ref.<br>- None/low exposure<br>- Medium/high exposure	1.00 RR<br>1.23 RR<br>1.35 RR	-<br>1.08-1.39<br>1.12-1.63	-<br>-<br>-	-<br>-<br>-	-<br>-<br>-	-<br>-<br>-
**Standing/walking**										
Alhalabi<br>2015	*Standing*: Prolonged standing was measured by if it is the most of the working time compared to “no awkward position” (N=911).	Chronic low back pain lasting ≥3 months.	None.	- None<br>- Prolonged	-<br>-	-<br>-	-<br>-	-<br>-	1.00 OR<br>1.69 OR	-<br>0.98 – 2.92
Euro<br>2019	*Standing*: Prolonged standing was measured by continuous or almost continuous standing typical for one’s work (N=3,891).	Hospitalisation of sciatica classified by ICD-8/9/10 codes.	Age and sex.	- No<br>- Yes	-<br>-	-<br>-	-<br>-	-<br>-	1.00 HR<br>1.01 HR	-<br>0.70 – 1.45
Heuch<br>2017	*Physical activity*: Occupational exposures was measured by physical activity at work divided into four categories (N=14,915).	Self-reported LBP in ≥3 months.	Age, leisure time activity, BMI, smoking, education, and occupational category.	- Sedentary work<br>- Walking at work and no heavy lifting	1.00 RR<br>0.96 RR	-<br>0.82 – 1.13	1.00 RR<br>1.11 RR	-<br>0.97 – 1.26	-<br>-	-<br>-
Matsudaira<br>2019	*Standing*: Standing was measured by hours standing in an average working day (N=196).	LBP interfering with work for ≥3 months.	None.	- <4 hours/day<br>- ≥4 hours/day	-<br>-	-<br>-	-<br>-	-<br>-	1.00 OR<br>1.06 OR	-<br>0.47 – 2.38
Seyedmehdi<br>2016	*Standing position in shift work*: Assessed by asking “what is your working postures most of the time?” with answers 1) always standing, 2) sometimes seated sometimes standing and 3) always standing (N=511).	Self-reported LBP lasting ≥3 months.	None.	- Sometimes<br>-Always					1.00 OR<br>1.25 OR	-<br>0.88 – 1.78
Vieira<br>2018	*Seated vs. standing*: Measured by if the participant worked seated or standing (N=184, 88 cases and 96 controls).	Disc degeneration.	Age and sex.	- Seated<br>- Standing	-<br>-	-<br>-	-<br>-	-<br>-	1.00 OR<br>0.38 OR	-<br>0.17 – 0.84
**Sitting**										

Aghilinejad 2015	*Sitting*: Duration of sustained sitting in a work day measured on a five-point scale and dichotomised (N=185, 49 cases and 136 controls).	Self-reported chronic pain for >3 months.	None.	- Low<br>- High	1.000 OR<br>1.237 OR	-<br>0.64 – 2.39	-<br>-	-<br>-	-<br>-	-<br>-
Ahsan 2013	*Sitting or standing*: Jobs that are performed in sitting or standing postures (employee in public, private, professionals, and students) cases (N=46) compared to controls (N=68).	Lumbar disc herniation.	Matched on age, sex, and area of residence.	- No<br>- Yes	-<br>-	-<br>-	-<br>-	-<br>-	1.00 OR<br>0.78 OR	-<br>NS
Alhalabi 2015	*Sitting*: Prolonged sitting was measured by if it is the most of the working time compared to "no awkward position" (N=911).	Chronic low back pain lasting >3 months.	Age.	- None<br>- Prolonged	-<br>-	-<br>-	-<br>-	-<br>-	1.00 OR<br>1.99 OR	-<br>1.18 – 3.36
Euro 2019	*Sitting*: Prolonged sitting was measured by continuous or almost continuous sitting typical for one's work (N=NS).	Hospitalisation of sciatica classified by ICD-8/9/10 codes.	Age, sex, BMI, educational level, smoking, physical sedentary work, heavy work, awkward trunk postures, lifting, and whole-body vibration.	- No<br>- Yes	-<br>-	-<br>-	-<br>-	-<br>-	1.00 HR<br>1.14 HR	-<br>0.76 – 1.71
Matsudaira 2015	*Desk work*: Sitting was measured by hours of desk work (≥half of the day as frequent) (N=167).	LBP interfering with work for >3 months.	None.	- Not frequent<br>- Frequent	-<br>-	-<br>-	-<br>-	-<br>-	1.00 OR<br>0.74 OR	-<br>0.30 – 1.81
Matsudaira 2014	*Desk work*: Was measured by hours of desk work longer than 6 hours per day (N=1,675).	LBP interfering with work for >3 months.	Age, sex, obesity, smoking habits, education, and manual handling of materials at work.	- <6 hours/day<br>- >6 hours/day	-<br>-	-<br>-	-<br>-	-<br>-	1.00 OR<br>0.66 OR	-<br>0.31 – 1.40
Picavet 2016	*Sitting*: workers were divided into sTable Ssitters or sTable Snon sitters based on the characterization of their job (N=1,509).	Self-reported chronic pain for >3 months.	Age, sex, education, working hours, smoking, complying with physical activity guideline, leisure time sitting, mental health, BMI, hypertension, and hypercholesterolemi a.	- Non-sTable Ssitters<br>- STable Ssitters	-<br>-	-<br>-	-<br>-	-<br>-	1.00 OR<br>1.17 OR	-<br>0.83 – 1.65
Seidler 2003	*Cumulative sedentary work*: Referring to the Nordic Questionnaire, sedentary work was measured in hours (N=182 controls and 129 cases).	Lumbar disc herniation combined with osteochondrosis/spo ndylosis.	Age, region, nationality, other disease of the lumbar spine, and sum lumbar spine	- <10,000 hours<br>- >10,000-30.000 hours<br>- >30,000 hours	1.0 OR<br>0.6 OR<br>1.0 OR	-<br>0.2 – 1.0<br>0.4 – 2.5	-<br>-<br>-	-<br>-<br>-	-<br>-<br>-	-<br>-<br>-

			force through lifting/carrying and/or extreme forward bending.							
**Combined exposures**										
Ahsan<br>2013	*Effort*: The physical effort exerted at work (N=200 cases and 200 controls).	Lumbar disc herniation.	Matched on age, sex, and area of residence.	- Sedentary or minimal<br>- Moderate<br>- Hard	-<br>-<br>-	-<br>-<br>-	-<br>-<br>-	-<br>-<br>-	0.43 OR<br>1.68 OR<br>3.14 OR	NS<br>NS<br>NS
Bergmann<br>2017	*Lumbar load and/or trunk inclination*:<br>10 dose models were applied comprising various thresholds for the lumbosacral compressive force, trunk inclination, or shift-related minimum threshold. Models consider loads 5 kg or more and trunk inclination from 20 to 90 degrees. The calculation includes frequency and duration of all handlings. A cumulative lifetime dose for the compressive force on the disc L5/S1 in kNh was computed (N=564 for LDH and N= 531 for SDN, whereas N=422 for controls).	Lumbar disc herniation (LDH) and severe disc narrowing (SDN).	Age, unemployment, work stress, manual materials handling, and study region.	LDH:<br>- 0 to <5.0*10^6Nh<br>- 5.0 to <21.51*10^6 Nh<br>- >21.51*10^6Nh<br>SDN:<br>- 0 to <5.0*10^6Nh<br>- 5.0 to <21.51*10^6 Nh<br>- >21.51*10^6Nh<br>LDH:<br>- 0 Nh<br>- >0 to <4.04*10^6Nh<br>- 4.04 to <14.47*10^6 Nh<br>- >14.47*10^6Nh<br>SDN:<br>- 0 Nh<br>- >0 to <4.04*10^6Nh<br>- 4.04 to <14.47*10^6 Nh<br>- >14.47*10^6Nh	 1.0 OR<br>1.9 OR<br>3.7 OR<br><br>1.0 OR<br>1.9 OR<br>4.1 OR<br><br>-<br>-<br>-<br>-<br><br>-<br>-<br>-<br>-	 -<br>1.1 – 3.0<br>2.3 – 6.0<br><br>-<br>1.0 – 3.7<br>2.2 – 7.6<br><br>-<br>-<br>-<br>-<br><br>-<br>-<br>-<br>-	 -<br>-<br>-<br><br>-<br>-<br>-<br><br>1.0 OR<br>2.2 OR<br>3.6 OR<br>3.5 OR<br><br>1.0 OR<br>1.5 OR<br>3.6 OR<br>3.3 OR	 -<br>-<br>-<br><br>-<br>-<br>-<br><br>-<br>1.3 – 3.8<br>2.1 – 6.1<br>2.0 – 5.9<br><br>-<br>0.8 – 3.0<br>1.9 – 6.8<br>1.8 – 5.9	 -<br>-<br>-<br><br>-<br>-<br>-<br><br>-<br>-<br>-<br>-<br><br>-<br>-<br>-<br>-	 -<br>-<br>-<br><br>-<br>-<br>-<br><br>-<br>-<br>-<br>-<br><br>-<br>-<br>-<br>-
Euro<br>2019	*Physical strenuousness of work*: Classified in six categories by the physical strenuousness of the participants' work (N=3,891).	Hospitalisation of sciatica classified by ICD-8/9/10 codes.	Age and sex.	- Light sedentary<br>- Other sedentary<br>- Physically light<br>- Fairly light/medium heavy<br>- Heavy<br>- Very heavy	-<br>-<br>-<br>-<br>-<br>-	-<br>-<br>-<br>-<br>-<br>-	-<br>-<br>-<br>-<br>-<br>-	-<br>-<br>-<br>-<br>-<br>-	1.00 HR<br>2.72 HR<br>1.78 HR<br>1.46 HR<br>0.92 HR<br>0.57 HR	-<br>1.36 – 5.44<br>1.06 – 3.02<br>0.86 – 2.50<br>0.49 – 1.75<br>0.17 – 1.90
	Physical strenuousness of work: Classified in six categories by the physical strenuousness of the participants work and then dichotomised by collapsing "Other sedentary work", "physically light work" and "Fairly light/medium heavy work" vs. all other categories (N=NS).	Hospitalisation of sciatica classified by ICD-8/9/10 codes.	Age, sex, BMI, educational level, smoking, heavy work, awkward trunk postures, prolonged sitting, and whole-body vibration.	- All other categories<br>- Other sedentary work, physically light work and fairly light/medium heavy work	-<br>-	-<br>-	-<br>-	-<br>-	1.00 HR<br>1.57 HR	-<br>1.05 – 2.34
	Physical strenuousness of work: Classified in six categories by the physical strenuousness of the	Hospitalisation of sciatica classified by ICD-8/9/10 codes.	Age, sex, BMI, educational level, smoking, awkward	- All other categories<br>- Heavy work and very heavy work					1.00 HR<br>0.48 HR	-<br>0.26 – 0.89

	participants work and then dichotomised by collapsing “Heavy” and “Very heavy” work vs. all other categories (N=NS).		trunk postures, prolonged sitting, and whole-body vibration.							
Heuch 2017	*Physical activity*: Occupational exposures was measured by physical activity at work divided into four categories (N=14,915).	Self-reported LBP in ≥3 months.	Age, leisure time activity, BMI, smoking, education, and occupational category.	- Sedentary work<br>- Walking at work and heavy lifting<br>- Particularly strenuous physical work	1.00 RR<br>1.08 RR<br>1.22 RR	-<br>0.90 – 1.29<br>1.01 – 1.49	1.00 RR<br>1.21 RR<br>1.24 RR	-<br>1.06 – 1.38<br>0.92 – 1.67	-<br>-<br>-	-<br>-<br>-
Jørgensen 2013	*Workload*: Was measured by from one question addressing ergonomic load to the back “is your work a) sedentary, b) slightly physical without lifting, c) physical with some lifting, or d) hard physical with heavy lifting, shovelling, or the like?” with the two first categories condensed (N=3,734).	Hospitalisation for lumbar disc disease.	Age.	- Low<br>- Medium<br>- High	1.00 HR<br>1.54 HR<br>2.80 HR	-<br>0.83 – 2.84<br>0.87 – 9.00	-<br>-<br>-	-<br>-<br>-	-<br>-<br>-	-<br>-<br>-
	Strenuous work: was measured by the question: “do you perform strenuous work (regularly resulting in sweating)?” (N=3,761).	Hospitalisation for lumbar disc disease.	Age, height, weight, and physical fitness.	- Seldom/never<br>- Occasionally<br>- Often	1.00 HR<br>2.37 HR<br>3.91 HR	-<br>1.36 – 4.13<br>1.82 – 8.38	-<br>-<br>-	-<br>-<br>-	-<br>-<br>-	-<br>-<br>-
Sørensen 2011	*Physical workload*: Was measured by one question addressing ergonomic load to the back “is your work a) sedentary, b) slightly physical without lifting, c) physical with some lifting, or d) hard physical with heavy lifting, shovelling, or the like?” with the two first categories condensed (N=3,724).	Hospitalisation for lumbar disc disease.	Age.	- Low<br>- Medium<br>- High	1.00 HR<br>1.54 HR<br>2.80 HR	-<br>0.83 – 2.84<br>0.87 – 9.00	-<br>-<br>-	-<br>-<br>-	-<br>-<br>-	-<br>-<br>-
	Strenuous workload: Was measured by the question “Do you perform strenuous work (regularly resulting in sweating)?” (N=3,761).	Hospitalisation for lumbar disc disease.	Age, height, and weight.	- Seldom/never<br>- Occasionally<br>- Often	1.00 HR<br>2.37 HR<br>3.90 HR	-<br>1.36 – 4.12<br>1.82 – 8.38	-<br>-<br>-	-<br>-<br>-	-<br>-<br>-	-<br>-<br>-
Seidler 2011	*Manual materials handling and/or intensive-load postures*: Assessed by a two-step procedure. 1) A standardised computer-assisted interview identifying subjects that exceeded a certain minimum workload, 2) Comprehensive semi-standardised interview by ergonomic expert with those exceeding minimum workloads.	Lumbar disc narrowing.	Men adjusted for region<br><br>Females adjusted for region and psychosocial workload	Men<br>- 0-<5.0*10^6 Nh<br>- 5.0–<21.51*10^6 Nh<br>- ≥21.51*10^6 Nh<br>Women<br>- 0 Nh<br>- >0–<4.04*10^6 Nh<br>- 4.04–<14.47*10^6 Nh<br>- ≥14.47*10^6 Nh	 1.0 OR<br>1.5 OR<br>3.1 OR<br><br>-<br>-<br>-<br>-	 -<br>0.9 – 2.8<br>1.8 – 5.2<br><br>-<br>-<br>-<br>-	 -<br>-<br>-<br><br>1.0 OR<br>1.2 OR<br>2.4 OR<br>2.0 OR	 -<br>-<br>-<br><br>-<br>0.7 – 2.2<br>1.4 – 4.1<br>1.2 – 3.3	 -<br>-<br>-<br><br>-<br>-<br>-<br>-	 -<br>-<br>-<br><br>-<br>-<br>-<br>-

	Quantification of compressive force on the lumbosacral disc assessed with a biomechanical tool (N=598 men and N=654 women).									
Seidler<br>2009	*Manual materials handling and/or intensive-load postures*: Assessed by a two-step procedure. 1) A standardised computer-assisted interview identifying subjects that exceeded a certain minimum workload, 2) Comprehensive semi-standardised interview by ergonomic expert with those exceeding minimum workloads. Quantification of compressive force on the lumbosacral disc assessed with a biomechanical tool (N=884 men and N=932 women).		Men, LDH: adjusted for age, region, and unemployment as severe life events<br><br>Men, LDN: adjusted for age and region<br><br>Women, LDH<br><br><br><br>Women, LDN:	LDH:<br>- 0 to <5.0*10^6Nh<br>- 5.0 to <21.51*10^6 Nh<br>- >21.51*10^6Nh<br>LDN:<br>- 0 to <5.0*10^6Nh<br>- 5.0 to <21.51*10^6 Nh<br>- >21.51*10^6Nh<br>LDH:<br>- 0 Nh<br>- >0 to <4.04*10^6Nh<br>- 4.04 to <14.47*10^6 Nh<br>- >14.47*10^6Nh<br>LDN:<br>- 0 Nh<br>- >0 to <4.04*10^6Nh<br>- 4.04 to <14.47*10^6 Nh<br>- >14.47*10^6Nh	 1.0 OR<br>1.7 OR<br>3.2 OR<br><br>1.0 OR<br>1.6 OR<br>3.2 OR<br><br>-<br>-<br>-<br>-<br><br>-<br>-<br>-<br>-	 -<br>1.1 – 2.7<br>2.2 – 5.0<br><br>-<br>0.9 – 2.8<br>1.9 – 5.5<br><br>-<br>-<br>-<br>-<br><br>-<br>-<br>-<br>-	 <br><br><br><br><br><br><br><br>1.0 OR<br>1.6 OR<br>2.4 OR<br>2.3 OR<br><br>1.0 OR<br>1.2 OR<br>2.3 OR<br>2.0 OR	 -<br>-<br>-<br><br>-<br>-<br>-<br><br>-<br>1.1 – 2.7<br>1.6 – 3.8<br>1.5 – 3.6<br><br>-<br>0.6 – 2.1<br>1.3 – 3.9<br>1.2 – 3.2	 -<br>-<br>-<br><br>-<br>-<br>-<br><br>-<br>-<br>-<br>-<br><br>-<br>-<br>-<br>-	 -<br>-<br>-<br><br>-<br>-<br>-<br><br>-<br>-<br>-<br>-<br><br>-<br>-<br>-<br>-
Seidler<br>2003	*Lifting/carrying combined with forward bending*:<br>to create a combined value for lumbar spine exposure to lifting or carrying and for trunk flexion, the Mainz-Dortmund dose model, which is based on over-proportional weighting of the lumbar disc compression force relative to the respective duration of lifting, was applied with modifications (N=187 controls and 129 cases).	Lumbar disc herniation combined with osteochondrosis/spondylosis.	Age, region, nationality, and other disease of the lumbar spine.	- No lifting/carrying; no extreme forward bending<br>- Lifting/carrying >0–150,000 kg^2*hours and/or extreme forward bending >0–1500 hours<br>- Lifting/carrying >150,000 kg^2*hours; extreme forward bending ≤1500 hours<br>- Lifting/carrying ≤150,000 kg^2*hours; extreme forward bending ≤1500 hours<br>- Lifting/carrying >150,00 kg^2*hours; extreme forward bending >1500 hours.	1.0 OR<br>4.3 OR<br>8.2 OR<br>8.2 OR<br>15.5 OR	-<br>1.8 – 10.2<br>3.2 – 20.9<br>2.4 – 28.7<br>5.2 – 46.9	-<br>-<br>-<br>-<br>-	-<br>-<br>-<br>-<br>-	-<br>-<br>-<br>-<br>-	-<br>-<br>-<br>-<br>-
**Other mechanical exposures**										
Aghilinejad<br>2015	*Hand above shoulder*: Duration of working with hands above shoulder in a working day measured on a five-point scale and dichotomised (N=185, 49 cases and 136 controls).	Self-reported chronic pain for ≥3 months.	None.	- Low<br>- High	1.00 OR<br>2.08 OR	-<br>0.98 – 4.12	-<br>-	-<br>-	-<br>-	-<br>-
Euro<br>2019	*Constant movements*: Was assessed in the domain “work postures and working methods” by the measurement: a constantly repeated series of movements (N=3,891).	Hospitalisation of Sciatica classified by ICD-8/9/10 codes.	Age and sex.	- No<br>- Yes	-<br>-	-<br>-	-<br>-	-<br>-	1.00 OR<br>0.94 OR	-<br>0.59 – 1.48

Herin<br>2014	*Movements*: Was measured by exposure to precise movements and/or repetitive work (N=1,206, 787 males and 419 female).	Self-reported LBP for ≥6 months.	Age, sports participation, BMI, and social class.	- Low<br>- High	1.00 OR<br>0.97 OR	-<br>0.84 – 1.12	1.00 OR<br>1.10 OR	-<br>0.86 – 1.41	-<br>-	-<br>-
	Forceful effort: Exposure to considerable physical effort (N= 787 males and 419 females)	Self-reported LBP for 6 months + clinical symptoms.	Age, sports participation, BMI, and social class.	- Low<br>- High	1.00 OR<br>1.20 OR	-<br>1.01 – 1.44	1.00 OR<br>1.21 OR	-<br>0.91 – 1.60	-<br>-	-<br>-
	Effort with tools: Exposure to physical effort with tools (N= 787 males and 419 females)	Self-reported LBP for 6 months + clinical symptoms.	Age, sports participation, BMI, and social class.	- Low<br>- High	1.00 OR<br>1.07 OR	-<br>0.88 – 1.32	1.00 OR<br>0.80 OR	-<br>0.44 – 1.46	-<br>-	-<br>-
Krause<br>2004	*Driving*: Years of professional driving was used as a proxy measure of past physical workload (N=1,233).	First incidence of low back injury.	Age, sex, ethnicity, personal factors and psychosocial job factors (psychological demands, decision latitude, supervisor support, co-worker support).	- ≤ 5 years<br>- 6-15 years<br>- >15 years<br><br>Less severe injuries:<br>- ≤ 5 years<br>- 6-15 years<br>- >15 years<br><br>More severe injuries:<br>- ≤ 5 years<br>- 6-15 years<br>- >15 years	-<br>-<br>-<br><br><br>-<br>-<br>-<br><br><br>-<br>-<br>-	-<br>-<br>-<br><br><br>-<br>-<br>-<br><br><br>-<br>-<br>-	-<br>-<br>-<br><br><br>-<br>-<br>-<br><br><br>-<br>-<br>-	-<br>-<br>-<br><br><br>-<br>-<br>-<br><br><br>-<br>-<br>-	1.36 HR<br>1.00 HR<br>0.86 HR<br><br><br>1.55 HR<br>1.00 HR<br>0.91 HR<br><br><br>1.05 HR<br>1.00 HR<br>0.69 HR	1.01 – 1.83<br>-<br>0.63 1.18<br><br><br>1.08 – 2.21<br>-<br>0.62 – 1.34<br><br><br>0.59 – 1.87<br>-<br>0.40 – 1.20
Matsudaira<br>2019	*Use of keyboard*: Measured by using a keyboard at work for more than four hours on an average working day (N=197).	LBP interfering with work for ≥3 months.	None.	- <4 hours/day<br>- ≥4 hours/day	-<br>-	-<br>-	-<br>-	-<br>-	1.00 OR<br>1.55 OR	-<br>0.63 – 3.79
	Move wrist/finger: Measured by other tasks involving repeated movements of the wrist or fingers for more than four hours on an average working day (N=198).	LBP interfering with work for ≥3 months.	None.	- <4 hours<br>- ≥4 hours	-<br>-	-<br>-	-<br>-	-<br>-	1.00 OR<br>0.90 OR	-<br>0.41 – 1.97
	Bend elbow: Measured by repeated bending and straightening of elbow for longer than one hour on an average working day (N=196).	LBP interfering with work for ≥3 months.	None.	- <1 hour<br>- ≥1 hour	-<br>-	-<br>-	-<br>-	-<br>-	1.00 OR<br>1.72 OR	-<br>0.62 – 4.75
	Hands above shoulder: Measured by working for longer than one hour with hands above shoulder height on an average working day (N=197).	LBP interfering with work for ≥3 months.	None.	- No<br>- Yes	-<br>-	-<br>-	-<br>-	-<br>-	1.00 OR<br>1.44 OR	-<br>0.66 – 3.15

	Driving: Measured by driving four or more hours on an average working day (N=197).	LBP interfering with work for ≥3 months.	None.	- <4 hours/day<br>- ≥4 hours/day	-<br>-	-<br>-	-<br>-	-<br>-	1.00 OR<br>0.72 OR	-<br>0.34 – 1.53
	Kneel/squat: Measured by kneel/squat during an average work day (N=197).	LBP interfering with work for ≥3 months.	None.	- <1 hour<br>- ≥1 hour	-<br>-	-<br>-	-<br>-	-<br>-	1.00 OR<br>0.76 OR	-<br>0.36 – 1.57
Matsudaira<br>2014	*Pushing*: Was measured by frequency of a working day considering half of the day as frequent (N=1,675).	LBP interfering with work for ≥3 months.	Age, sex, obesity, smoking habits, and education.	- Infrequent<br>- Frequent	-<br>-	-<br>-	-<br>-	-<br>-	1.00 OR<br>3.48 OR	-<br>1.24 – 9.76
Prado-Leon<br>2014	*Pushing/pulling*: Measured by jobs that subjects had performed in the past involving pushing/pulling (N=231, 77 cases and 154 controls).	Spondyloarthrosis.	Lifting, carrying, and driving.	- No<br>- Yes	-<br>-	-<br>-	-<br>-	-<br>-	1.0 OR<br>2.4 OR	-<br>1.1 – 4.7
	Pushing/pulling: Former jobs working with pushing/pulling measured by weight of load (kg.) when pushing/pulling (N=231, 77 cases and 154 controls).	Spondyloarthrosis.	Lifting, carrying, and driving.	- 0<br>- 20-60 kg.<br>- >60-500 kg.	-<br>-<br>-	-<br>-<br>-	-<br>-<br>-	-<br>-<br>-	1.0 OR<br>1.7 OR<br>2.9 OR	-<br>0.6 – 4.8<br>1.2 – 7.3
	Pushing/pulling: Hours spent daily on work with pushing/pulling in formers jobs (N=231, 77 cases and 154 controls).	Spondyloarthrosis.	Lifting, carrying, and driving.	- 0<br>- 1-7 hours<br>- >7-10 hours	-<br>-<br>-	-<br>-<br>-	-<br>-<br>-	-<br>-<br>-	1.0 OR<br>2.0 OR<br>2.6 OR	-<br>0.7 – 6.0<br>1.0 – 7.4
	Pushing/pulling: Time spent pushing/pulling in former jobs measured by months and years (N=231, 77 cases and 154 controls).	Spondyloarthrosis.	Lifting, carrying, and driving.	- 0-9 months<br>- 1 year and 8 m. to 9 years<br>- 10-32 years	-<br>-<br>-	-<br>-<br>-	-<br>-<br>-	-<br>-<br>-	1.0 OR<br>2.3 OR<br>1.9 OR	-<br>0.8 – 6.5<br>0.7 – 5.2
	Pushing/pulling: Frequency of pushing/pulling was measured by 25 or more times per day (N=231, 77 cases and 154 controls).	Spondyloarthrosis.	Lifting, carrying, and driving.	- No<br>- Yes	-<br>-	-<br>-	-<br>-	-<br>-	1.0 OR<br>2.7 OR	-<br>0.8 – 8.8
	Pushing/pulling: Daily job frequency performing tasks of pushing/pulling was measured by yes or no (N=231, 77 cases and 154 controls).	Spondyloarthrosis.	Lifting, carrying, and driving.	- No<br>- Yes	-<br>-	-<br>-	-<br>-	-<br>-	1.0 OR<br>3.7 OR	-<br>1.4 – 9.5
Tubach<br>2004	*Driving*: Was measured by driving >2 hours a day (N=317).	Sciatica	Gender.	- Never<br>- <Once a week<br>- >Once a week<br>- Everyday	-<br>-<br>-<br>-	-<br>-<br>-<br>-	-<br>-<br>-<br>-	-<br>-<br>-<br>-	1.00 OR<br>2.37 OR<br>1.79 OR<br>1.01 OR	-<br>1.16 – 4.87<br>0.97 – 3.32<br>0.62 – 1.63

Appendix H. Funnel plots

Figure 8. Funnel plots for each occupational mechanical exposure.

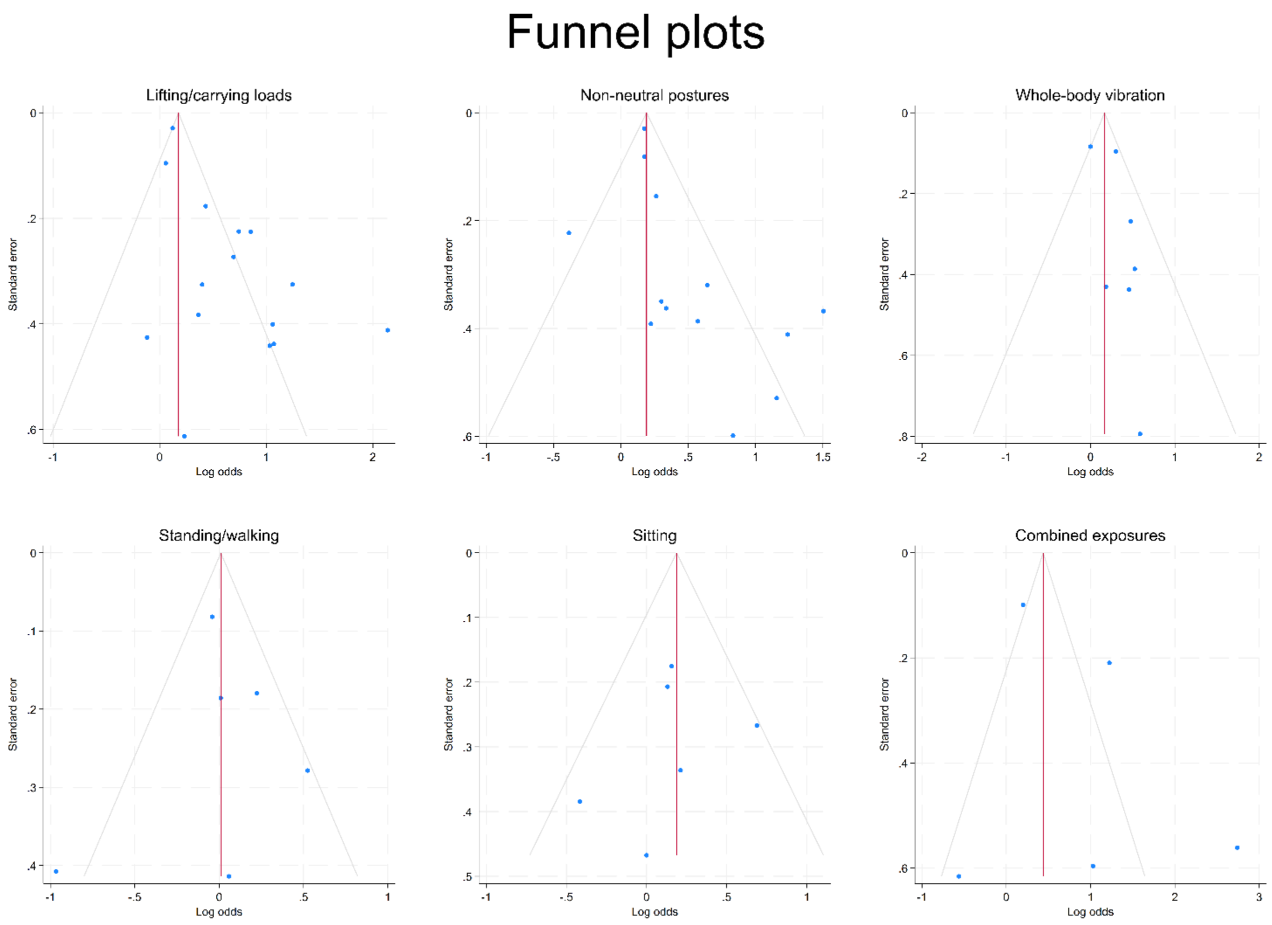

Appendix I. Exposure-response relations.

We assessed only exposures graded as “Moderate certainty of evidence” for an exposure-response relation. First, we examined whether trend tests were conducted. If trend test were not conducted, we graphically visualised potential exposure-response relation using QQ-plot, if studies provided $\geq$3 levels of an exposure assessment.

Lifting/carrying loads
Three articles from the meta-analysis evaluated exposure-response relation. Using hierarchical regression analyses, Jansen et al. presents exposure-response relation between lifting and carrying loads >10 kg and risk of LBP with disability resulting in “more linear than the conventional model” changing from non-monotonic or inconsistent. Latza et al. examined a linear trend test between laying large lime sandstone (6 to 10 kilograms) and chronic LBP using Wald test and showing a p-value under 5% across 3 levels the exposure. Seidler et al. from 2003 calculated a test for trend using exposure categories as interval scaled variables in a logistic regression model showing a significant exposure-response gradient on cumulated lifting/carrying.

Figure 9. Eight studies of lifting/carrying loads provided measure of association for ≥3 exposure groups.

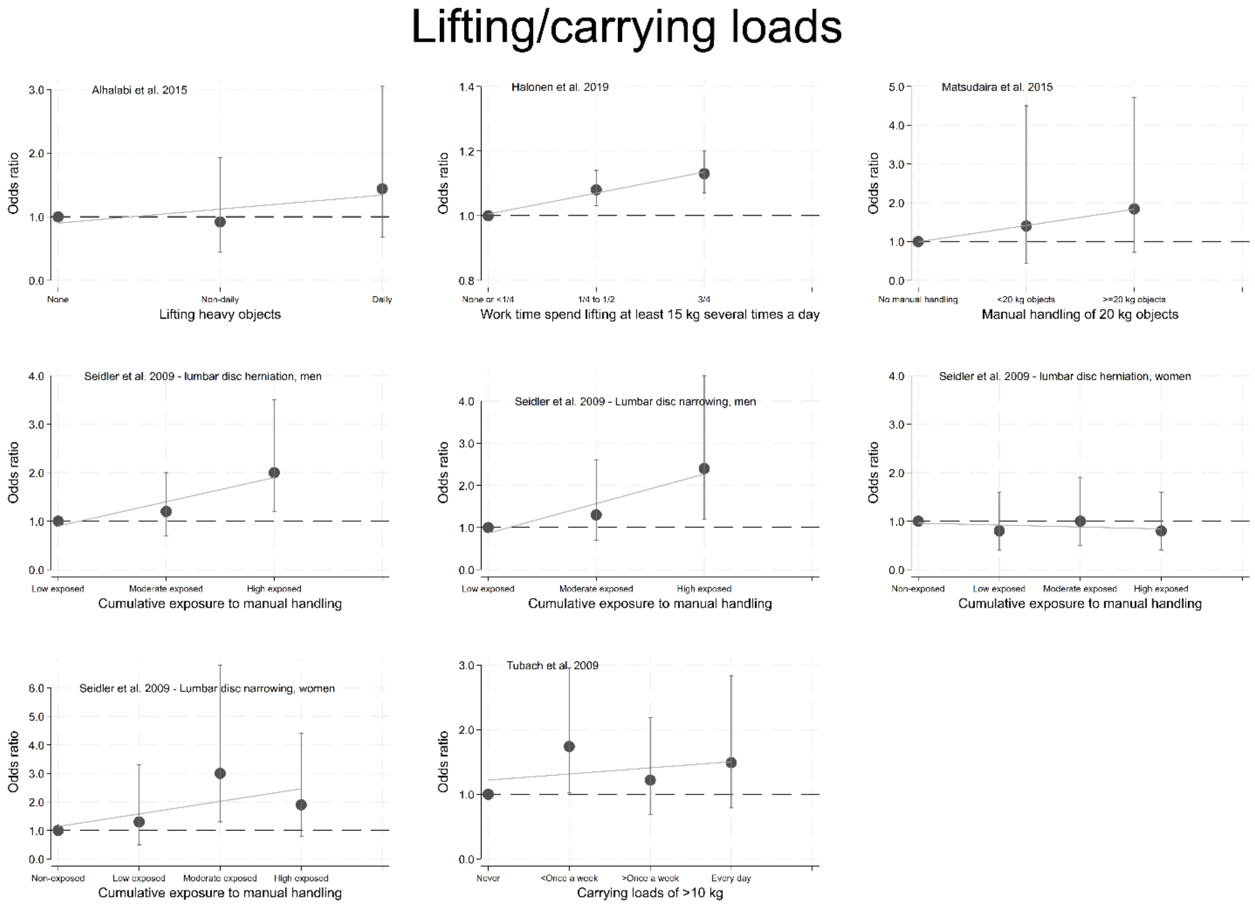

Non-neutral postures
Jansen et al. found both in the conventional and hierarchical model increasing risk estimates that increased with time spend in trunk flexion over 45 degrees per week. Seidler et al., 2009, found a significant exposure-response gradient for extreme forward bending (>90°) and risk of lumbar disc herniation with osteochondrosis/spondylosis.

Figure 10. Five studies of non-neutral postures presented measure of association for ≥3 exposure groups.

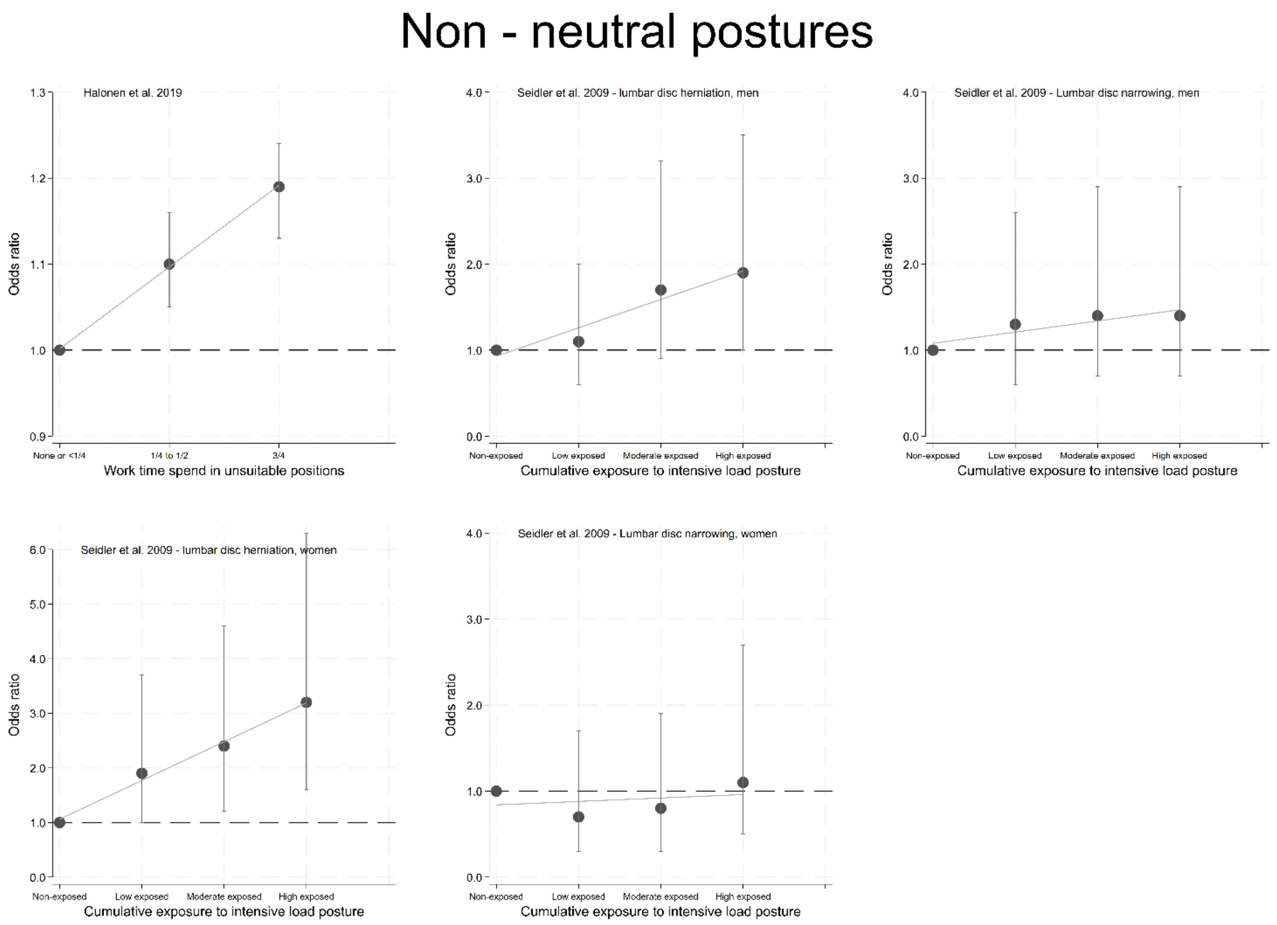

Combined mechanical exposures
Seidler et al. from 2009 found a positive exposure-response relation between cumulative lumbar load (manual handling of objects of about 5 kilograms or more and/or intensive load postures) and lumbar disc disease (herniation and disc narrowing) for both men and women.

Figure 11. Four studies of combined mechanical exposures presented measure of association for ≥3 exposure groups.

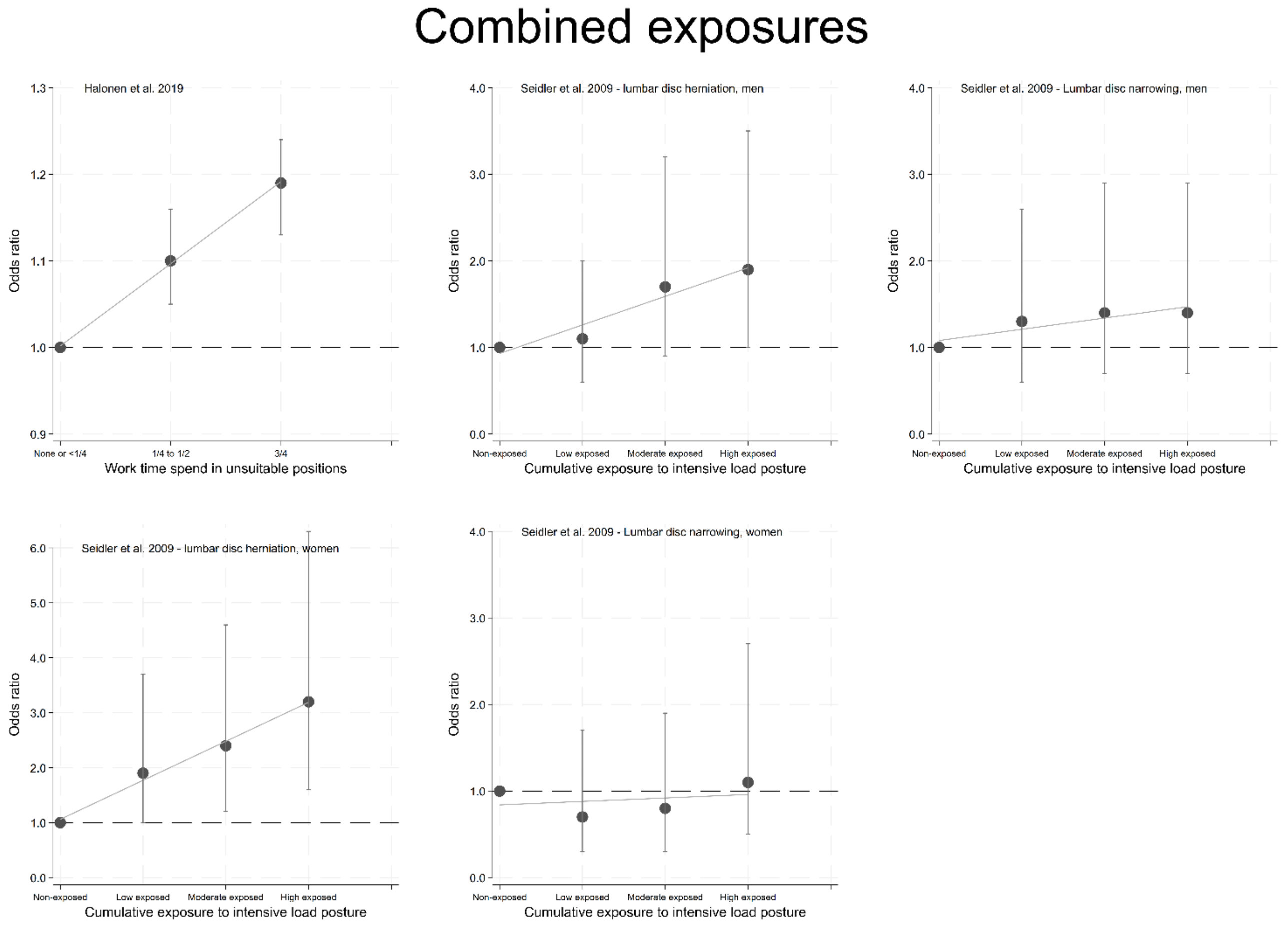

Supplement: Supplementary material [file SJWEH-49-453-S001.pdf]
